# **α**-Ketoglutarate protects against cartilage damage via epigenetically driven metabolic reprogramming in osteoarthritis models

**DOI:** 10.1172/JCI172380

**Published:** 2026-03-02

**Authors:** Shuaijun Li, Jiefeng Huang, Ting Shang, Laiya Lu, Orion R. Fan, Peisheng Jin, Xin Zou, Zixin Cai, Wuyan Lu, Shuangmeng Jia, Linxiao Li, Ke Fang, Fengting Niu, Jiaojiao Li, Cheng Zhao, Qian Wang, Ruizhu Sun, Si Shi, Feng Yin, Yun Zhang, Yi Eve Sun, Lei Cui

**Affiliations:** 1 Department of Plastic Surgery, Shanghai Tenth People’s Hospital and; 2 Stem Cell Translational Research Center, School of Medicine, Tongji University, Shanghai, China.; 3 Department of Plastic Surgery, Beijing Shijitan Hospital affiliated with Capital Medical University, Beijing, China.; 4 Department of Joint Surgery and; 5 Institute for Regenerative Medicine, Shanghai East Hospital, Tongji University School of Medicine, Shanghai, China.; 6 Department of Plastic Surgery, Affiliated Hospital of Xuzhou Medical University, Xuzhou, China.; 7 Jinshan Hospital Center for Tumor Diagnosis & Therapy, Jinshan Hospital, Fudan University, Shanghai, China.; 8 Faculty of Life and Health Sciences, Shenzhen University of Advanced Technology, Shenzhen, Guangdong, China.; 9 Department of Plastic Surgery, First Affiliated Hospital of Xinjiang Medical University, Urumqi, China.

**Keywords:** Inflammation, Metabolism, Cartilage

## Abstract

The link between glutaminolysis and osteoarthritis (OA) has only recently begun to be elucidated. Here, we report the association of obesity- and injury-induced cartilage damage with impaired glutaminolysis in chondrocytes. Defective glutaminolysis triggered the onset and progression of OA, with enhanced catabolism and decreased anabolism. Supplementation of α-ketoglutarate (αKG), a key component in glutaminolysis and an epigenetic factor, effectively protected cartilage against degradation in vivo via a TCA cycle– and HIF-1α–independent manner. Mechanistically, OA pathogenic factors increased H3K27me3 deposition on promoters of key glutaminolysis genes, including *Slc1a5* and *Gls1*, leading to impaired glutaminolysis. Conversely, αKG facilitated Kdm6b-dependent H3K27me3 demethylation of not only glutaminolysis genes to rescue Gln metabolism but also *Ube2o* to reverse OA. Elevated *Ube2o* expression led to TRAF6 ubiquitination and subsequent inhibition of NF-κB signaling, thereby reversing the pathological reprogramming of glycolysis and oxidative phosphorylation and protecting against cartilage destruction. Collectively, these results demonstrated that OA pathogenic factors impair glutaminolysis through epigenetic regulation, which further exacerbate OA. Moreover, αKG restores metabolic homeostasis and alleviates OA through H3K27me3 demethylation.

## Abstract 2

α-KG protects cartilage in osteoarthritis through epigenetic mechanisms, offering a new therapeutic strategy.

## Graphical abstract

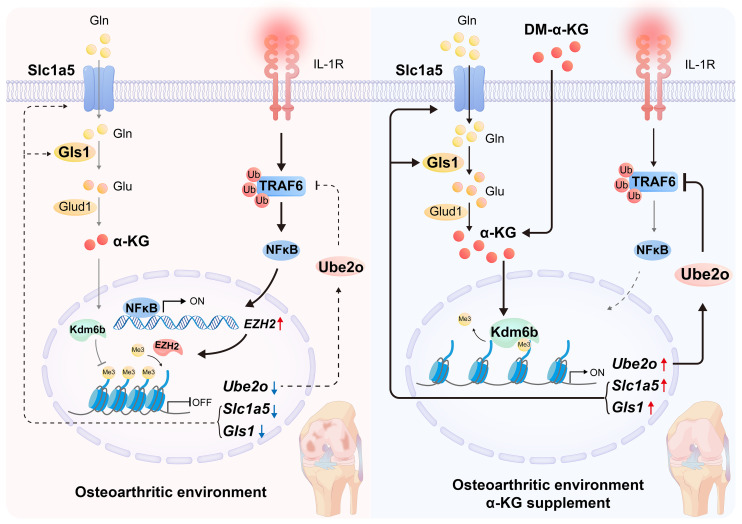

## Introduction

Osteoarthritis (OA) is the most common joint cartilage disease worldwide and the leading cause of disability in elderly populations (1, 2). Currently, therapies for OA are typically limited to symptomatic relief, due to a lack of effective disease-modifying treatments; joint arthroplasty often is required at the late stage (3). Homeostasis in healthy cartilage is maintained by the balance between physiological anabolism and catabolism in chondrocytes (4). During the pathogenesis of OA, proinflammatory cytokines are triggered by aberrant mechanical and biochemical stimuli, with subsequent activation of the NF-κB signaling pathway in chondrocytes (5). Once activated, NF-κB signaling drives the expression of pathological catabolic factors, including MMP3 and MMP13, aggrecanase-1/2 (ADAMTS4/5), and inducible nitric oxide synthase 2 (NOS2), leading to progressive destruction of articular cartilage (6).

Increasing evidence has demonstrated that certain subtypes of OA are caused by metabolic abnormalities (7, 8). Alterations in systemic metabolism, such as metabolic syndrome, diabetes mellitus (9), and cardiovascular disease (10), have all been comorbid with worsening of OA pathology. The role of altered glycolysis and lipid metabolism has been recently well defined in triggering OA pathogenesis (7). However, though alterations in amino acid metabolism, such as glutaminolysis, have been implicated in a wide range of diseases (11, 12), their potential roles have not been fully elucidated in OA. Glutamine (Gln) is essential for cancer cell survival and proliferation because it is a significant source of carbon and nitrogen. Gln is actively transported across the cell membrane by amino acid transporters, such as solute carrier family 1 member 5 (*Slc1a5)* (13). Gln can then be enzymatically converted by glutaminase (*Gls1*) into glutamate (Glu), which can then be further metabolized into α-ketoglutarate (αKG) to participate in the TCA cycle (14). A recent study reported that Gln controls chondrogenic gene expression epigenetically through Glu dehydrogenase–dependent acetyl-CoA synthesis (15). However, whether alterations in Gln metabolism are linked to osteoarthritis remains largely unknown.

Recent evidence has demonstrated the role of epigenetic dysregulation of multiple genes in the pathogenesis of OA, with many of these genes undergoing DNA demethylation and histone modification (16, 17). For example, Kdm6b has been identified as an H3K27 demethylase that can catalyze the demethylation of H3K27me2/3 (18). Knockdown of *Kdm6b* in chondrocytes leads to abnormal cartilage development and accelerated OA progression via inhibition of anabolic metabolism (19). Jumonji-domain–containing histone demethylases (JHDMs), a subset of histone demethylases (HDMs), use αKG, oxygen, and Fe (II) as cofactors, leading to release of succinate and formaldehyde as byproducts (20). The requirement of αKG for HDM activity suggests a potential connection between metabolism and epigenetic modifications. Recent research has shown that Gln deprivation in embryonic stem cells can lead to enhanced methylation on H3K27me3 (21). However, it remains unknown whether Gln metabolism plays any role in cartilage homeostasis or epigenetic modifications.

In this study, the impact of altered Gln metabolism on OA pathology was investigated through a series of gain- and loss-of-function analyses in both surgically induced and obesity-related OA mouse models. The reciprocal modulation between glutaminolysis and OA pathogenic factors was examined both in vitro and in vivo, revealing an epigenetic link.

## Results

### Gln metabolism is impaired in OA chondrocytes.

Obesity is a well-established risk factor for OA (22). Our 2-sample Mendelian randomization analysis provided genetic evidence that both grade 1 obesity and OA are causally associated with reduced blood Gln levels. The substantial negative causal effect of genetically predicted grade 1 obesity was corroborated by sensitivity analyses showing no evidence of heterogeneity or horizontal pleiotropy (Supplemental Figure 1, A–D; supplemental material available online with this article; https://doi.org/10.1172/JCI172380DS1). Similarly, a marked negative causal relationship was identified for OA, which remained robust after outlier exclusion and across various sensitivity analyses (Supplemental Figure 1, E–H). These findings collectively establish both conditions as causal factors in lowering blood Gln levels.

**Figure 1:**
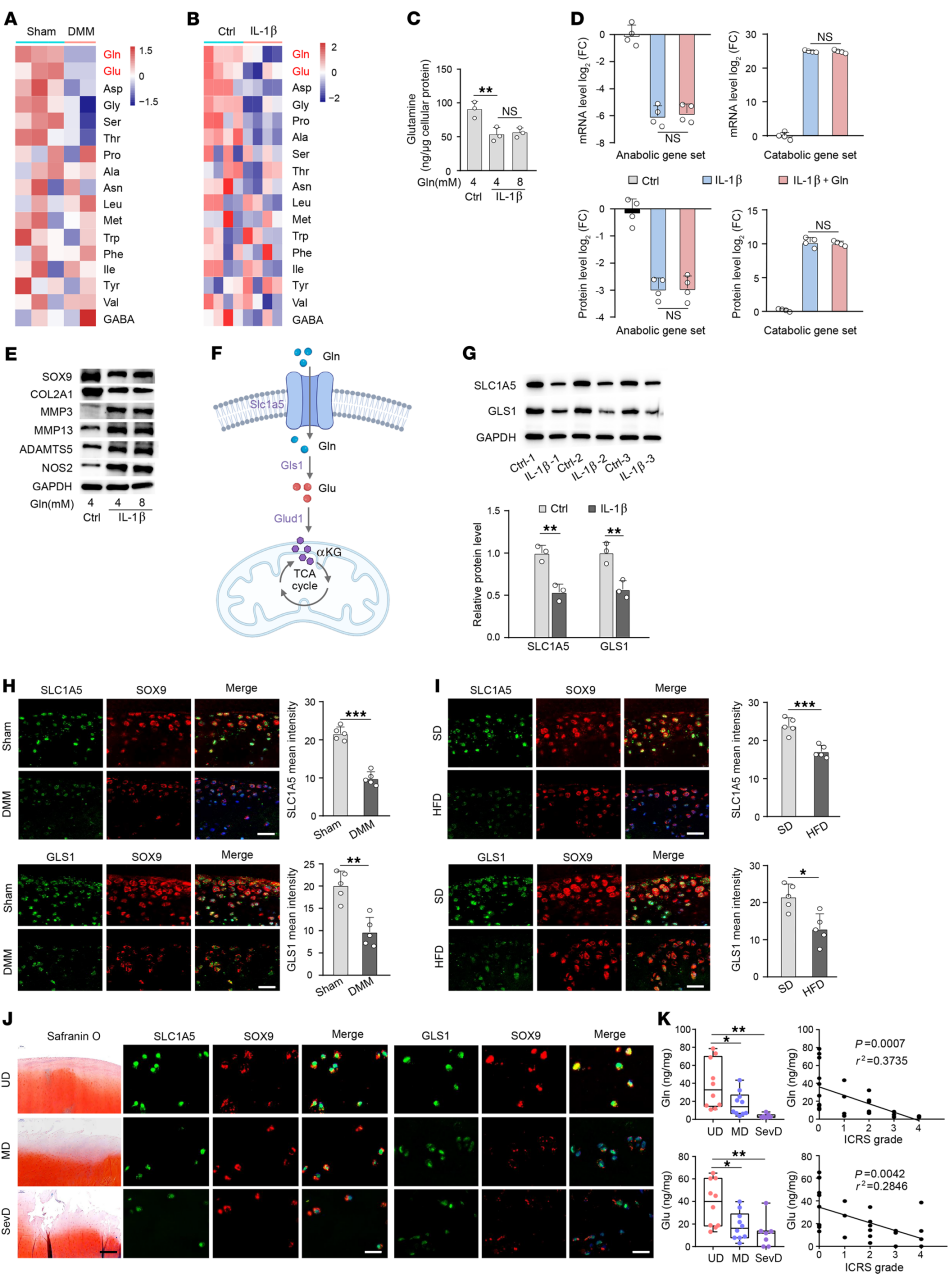
OA cartilage had lower Gln levels and reduced glutaminolysis. (**A**) Heatmap of amino acid content analyzed by LC-MS in sham- and DMM-operated knee cartilage (*n* = 3 and 2, respectively) of mice at 8 weeks after surgery. (**B**) Heatmap of amino acid levels analyzed by LC-MS in chondrocytes treated with or without IL-1β (5 ng/mL) for 36 hours (*n* = 4 per group). Ctrl, control. (**C**) Intracellular Gln levels in IL-1β–treated chondrocytes supplemented with Gln (4 or 8 mM) for 24 hours (*n* = 3 per group). (**D** and **E**) Quantitative analysis of qRT-PCR (*n* = 4 per group) (**D**) and Western blot (**E**) analysis for the indicated anabolic and catabolic factors in IL-1β–treated chondrocytes supplemented with Gln (4 or 8 mM) for 24 hours. (**F**) Schematic of Gln metabolism. (**G**) Western blot analysis for SLC1A5 and GLS1 in chondrocytes treated with or without IL-1β for 24 hours (*n* = 3 per group). (**H**) Representative images of SLC1A5 and GLS1 immunofluorescence and mean intensity in joint cartilage of sham- or DMM-operated mice at 8 weeks after surgery (*n* = 5 mice per group). Scale bars: 20 μm. (**I**) Representative images of SLC1A5 and GLS1 immunofluorescence and mean intensity in mice fed a standard diet (SD) and those fed an HFD (*n* = 5 mice per group). Scale bars: 20 μm. (**J**) Representative images from safranin O staining and SLC1A5 and GLS1 immunofluorescence in undamaged (*n* = 10), mildly damaged (*n* = 10), and severely damaged (SevD) (*n* = 7) human OA cartilage. Scale bars: 20 μm. (**K**) Gln and Glu levels in undamaged (UD) (*n* = 10), mildly damaged (MD) (*n* = 10) and SevD (*n* = 7) human OA cartilage. The correlation between Gln levels and ICRS grading scores is shown in the right column. Scale bars: 200 μm. The data are presented as box plots or as the mean ± SEM, and the dots represent biological replicates. *P < 0.05, **P < 0.01, ***P < 0.001.

We next sought to characterize the role of Gln metabolism in OA pathogenesis. Through liquid chromatography–mass spectrometry (LC-MS) analysis, Gln and Glu were found to be the most downregulated amino acids in cartilage tissues of mice after destabilization of the medial meniscus (DMM) as well as IL-1β–treated chondrocytes (Figure 1, A and B, and Supplemental Figure 2A). Subsequent experiments on mouse articular chondrocytes treated with IL-1β demonstrated a time-dependent decrease in Gln levels (Supplemental Figure 2B). However, supplementation of Gln (8 mM) did not augment intracellular Gln levels, nor did it influence the expression of anabolic and catabolic factors in IL-1β–treated chondrocytes (Figure 1, C–E, and Supplemental Figure 2, C and D). The amino acid transporter *Slc1a5* is the principal mediator of Gln uptake in mammalian cells (Figure 1F). Microarray analyses showed that upon IL-1β stimulation, multiple key factors of glutaminolysis, including Gln transporter *Slc1a5*, *Gls1*, and Glu dehydrogenase (*Glud1*), were all downregulated (Supplemental Figure 2E), The downregulation of Slc1a5 and Gls1 was validated at both mRNA and protein levels (Figure 1G and Supplemental Figure 2F). Additionally, reduced expression of SLC1A5 and GLS1 as well as decreased Gln levels were observed in the knee cartilage of DMM mice (Figure 1H and Supplemental Figure 2, A and G).

To explore the link with obesity, we compared mice fed a high-fat diet (HFD) with those fed a standard diet. HFD-fed mice exhibited more severe cartilage destruction and a near-complete loss of the cartilage layer 8 weeks after DMM surgery (Supplemental Figure 2, H, J, and K). In obese individuals, the levels of circulating inflammatory factors were elevated with concomitant decrease in Gln levels (23). Furthermore, obese mice demonstrated decreased levels of SLC1A5 and GLS1 mRNA and protein expression, as well as lower Gln levels in cartilage, compared with lean control mice (Figure 1I and Supplemental Figure 2, A and I), indicative of Gln transporter impairment in obese mice.

To support the human relevance of these findings, the levels of amino acids were compared between undamaged and damaged regions in OA cartilage from knee joints of individuals who had undergone arthroplasty. LC-MS analyses revealed that levels of Gln and Glu were also decreased in damaged human OA cartilage (Supplemental Figure 2L). In addition, undamaged cartilage from humans with OA exhibited increased SLC1A5 and GLS1 mRNA and protein levels compared with moderately and severely damaged counterparts (Figure 1J and Supplemental Figure 2, M and N), indicative of conservation among species. The levels of Gln and Glu decreased with severity of OA and inversely correlated with International Cartilage Repair Society (ICRS) grading scores (Figure 1K). Together, these results demonstrate that glutaminolysis was impaired in OA and could not be rescued by exogenous Gln supplementation due to reduced expression of Gln transporter SLC1A5.

### Defective glutaminolysis induced the onset and progression of OA.

**Figure 2:**
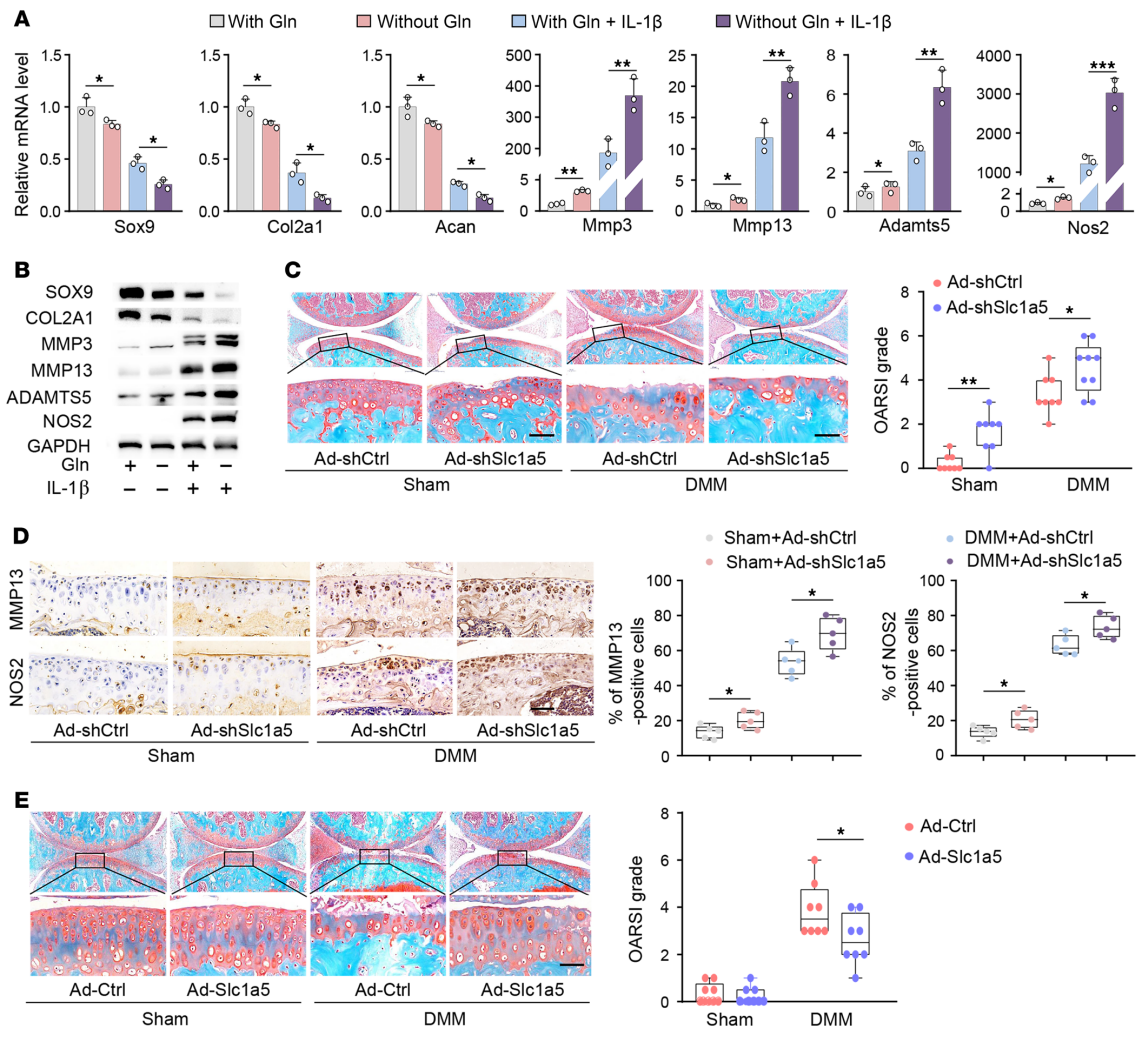
SLC1A5 mediates the pathogenesis of OA. (**A**) qRT-PCR (*n* = 3 per group) and (**B**) Western blot of anabolic and catabolic factors in IL-1β–treated chondrocytes with (w) or without (w/o) Gln conditions for 24 hours. Blots are representative of 3 independent experiments. (**C**) Representative images of safranin O/fast green staining, OARSI scores in mice at 8 weeks after sham and DMM surgery. Ad-shCtrl or Ad-shSlc1a5 was intra-articularly injected once per week for 3 weeks beginning at 10 days after surgery. Sham+Ad-shCtrl (*n* = 8), Sham+Ad-sh*Slc1a5* (*n* = 8), DMM+Ad-shCtrl (*n* = 8), DMM+Ad-sh*Slc1a5* (*n* = 9). (**D**) Representative images of IHC staining and quantification of MMP13- and NOS2-positive cells in joint sections of mice at 8 weeks after sham and DMM surgery, Ad-shCtrl or Ad-sh*Slc1a5* was intra-articularly injected once per week for 3 weeks beginning at 10 days after surgery. *n* = 5 mice per group. (**E**) Representative safranin O/fast green staining images and OARSI scores (*n* = 8–9 mice per group) in Ad-Ctrl or Ad-*Slc1a5* mice at 8 weeks after sham and DMM surgery. The data are presented as box plots or as the mean ± SEM, and the dots represent biological replicates. One-way ANOVA with Tukey’s multiple comparisons tests were used for statistical analyses in **A** and **D**; Mann-Whitney *U* tests for OARSI grades were used in **C** and **E**. *P < 0.05, **P < 0.01, ***P < 0.001.

To address how Gln deficiency is associated with OA, catabolic and anabolic factors in chondrocytes were examined upon Gln deprivation, a condition of which resulted in slight but substantial increases in all 4 catabolic factors (MMP3, MMP13, ADAMTS5, and NOS2) and decreases in 3 anabolic factors (SOX9, COL2A1, and ACAN) (Figure 2, A and B, and Supplemental Figure 3A). Upon IL-1β treatment, the imbalance between anabolic and catabolic factors was further exacerbated in chondrocytes deprived of Gln (Figure 2, A and B, and Supplemental Figure 3A). *Slc1a5* knockdown in primary-culture chondrocytes markedly reduced Gln levels, decreased anabolic factors, and elevated catabolic factors (Supplemental Figure 3, B–E). A similar phenotype was observed in chondrocytes treated with a pharmacological inhibitor of *Slc1a5* (γ-l-glutamyl-*p*-nitroanilide [GPNA]) (Supplemental Figure 3F). Furthermore, the imbalance between anabolic and catabolic factors was further exacerbated in the presence of IL-1β (Supplemental Figure 3, D and E), whereas overexpression of Ad-*Slc1a5* ameliorated the highly imbalanced anabolism and catabolism in IL-1β–treated chondrocytes (Supplemental Figure 3, G–I).

The in vivo functions of *Slc1a5* during the pathogenesis of OA were investigated by gene overexpression as well as gene-knockdown approaches in mouse joint tissues. *Slc1a5* knockdown by intra-articular injection of Ad-sh*Slc1a5* induced mild cartilage degradation in sham-operated cartilage, and exacerbated cartilage destruction in DMM-treated cartilage with higher Osteoarthritis Research Society International (OARSI) scores, as well as increased numbers of MMP13- and NOS2-positive cells at 8 weeks after surgery (Figure 2, C and D, and Supplemental Figure 3J). The role of *Slc1a5* was further validated in whole-joint tissues via intra-articular injection of an adenoviral vector carrying the *Slc1a5* overexpression cassette (Supplemental Figure 3K). Intra-articular administration of Ad-*Slc1a5* rescued matrix destruction in DMM-operated cartilage and reduced numbers of MMP13- and NOS2-positive cells (Figure 2E and Supplemental Figure 3L). Collectively, these results suggest Gln deficiency mediates the pathogenesis of OA.

**Figure 3:**
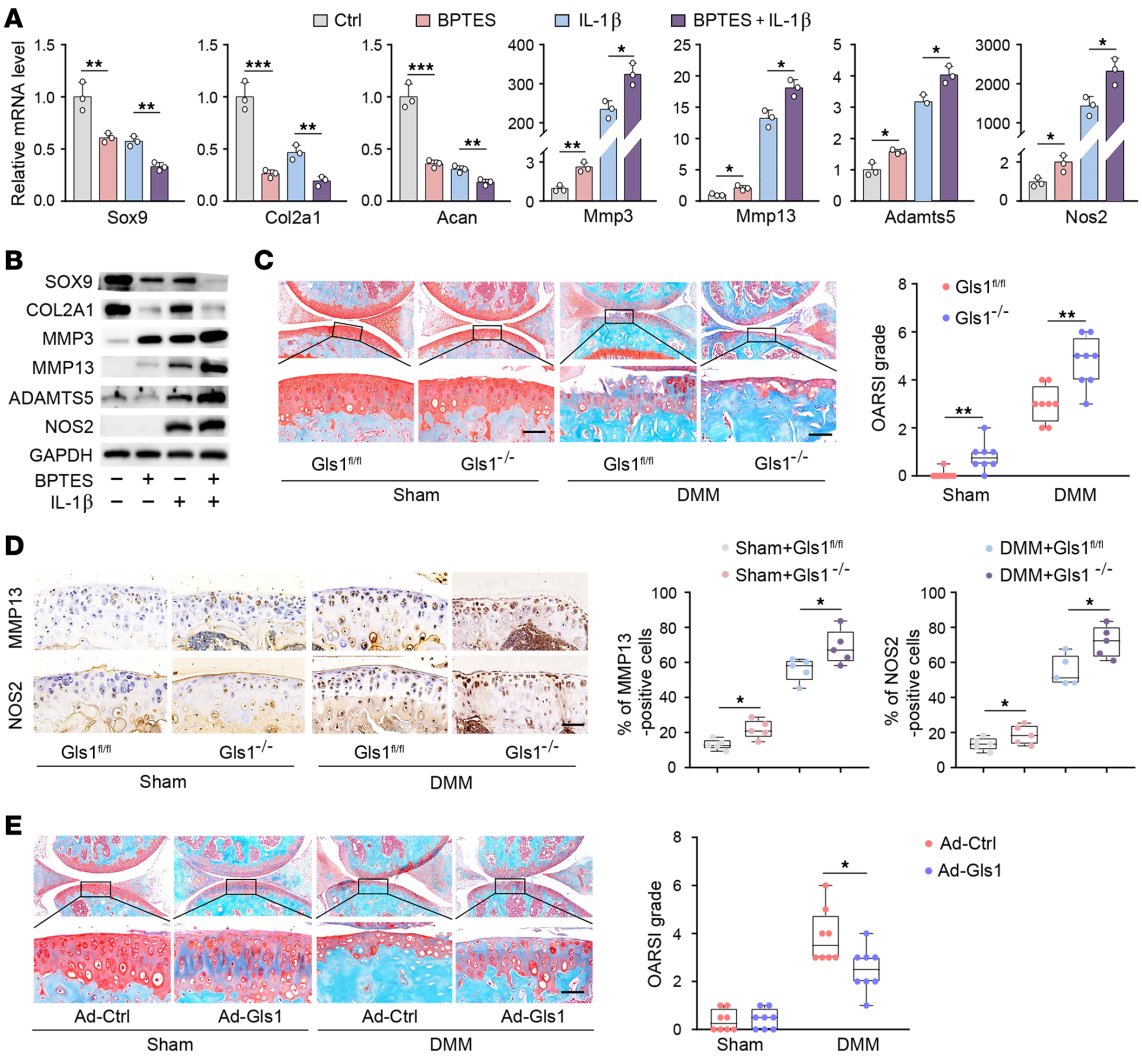
GLS1 plays a key mechanistic role in the development of OA. (**A** and **B**) mRNA (**A**) and Western blot (**B**) analysis of the indicated anabolic and catabolic factors in chondrocytes treated with BPTES or IL-1β alone or with combined BPTES and IL-1β for 24 hours. The culture medium contained 4 mM Gln. Blots are representative of 3 independent experiments. Ctrl, control. (**C**) Representative safranin O/fast green staining images and OARSI scores (*n* = 8 mice per group) in *Gls1*^−/−^ mice and their *Gls1*^fl/fl^ control littermates at 8 weeks after sham and DMM surgery. (**D**) Representative MMP13 and NOS2 IHC staining images and quantification of cells positive for MMP13 and NOS2 (*n* = 5 mice per group) in *Gls1*^−/−^ mice and their *Gls1*^fl/fl^ control littermates at 8 weeks after sham and DMM surgery. (**E**) Safranin O/fast green staining and OARSI scores in joint sections of mice at 8 weeks after DMM surgery. Ad-Ctrl or Ad-*Gls1* was intra-articularly injected once per week for 3 weeks beginning at 10 days after surgery (*n* = 8 mice per group). Scale bars: 20 μm. The data are presented as box plots or as the mean ± SEM, and the dots represent biological replicates. *P < 0.05, **P < 0.01, ***P < 0.001.

*Gls1* acts as the rate-limiting enzyme catalyzing hydrolysis of Gln into Glu during glutaminolysis. Pharmacological inhibition of *Gls1* upregulated expression of the catabolic gene set and downregulated the anabolic gene set, with or without IL-1β treatment (Figure 3, A and B, and Supplemental Figure 4, A and B). Overexpression of *Gls1* in primary chondrocyte cultures attenuated IL-1β–induced increases in catabolic genes and restored the expression of anabolic genes (Supplemental Figure 4, C–E). The in vivo function of *Gls1* during OA pathogenesis was analyzed using genetic ablation of *Gls1* and adenoviral vector–mediated *Gls1* overexpression (Ad-*Gls1*) in mice. By generating chondrocyte-specific *Gls1*–conditional KO (cKO) mice, spontaneous development of mild OA in knee joint could be observed even without DMM surgery at 8 weeks after tamoxifen injection (Figure 3C and Supplemental Figure 4F). Four weeks later, after DMM surgery, cKO mice had already developed OA with remarkable cartilage destruction and higher OARSI scores, whereas control DMM mice had only mild cartilage degradation (Supplemental Figure 4G). By 8 weeks after surgery, cKO mice had extensive loss of cartilage across the entire surface of the knee joint, with substantially increased OARSI scores and percentages of MMP13- and NOS2-positive cells in joint cartilage (Figure 3, C and D). The role of GLS1 was further validated in whole-joint tissues through adenoviral vector–mediated overexpression of *Gls1* after intra-articular viral injection. Cartilage destruction and percentages of MMP13- and NOS2-positive cells were partly inhibited by *Gls1* overexpression in mice (Figure 3E and Supplemental Figure 4, H and I). Together, these results demonstrated that defective glutaminolysis in chondrocytes resulted in an imbalance of anabolism and catabolism in chondrocytes, which accelerated the onset and progression of OA.

### Supplementation of αKG rescued the anabolic/catabolic imbalance caused by defective glutaminolysis.

**Figure 4:**
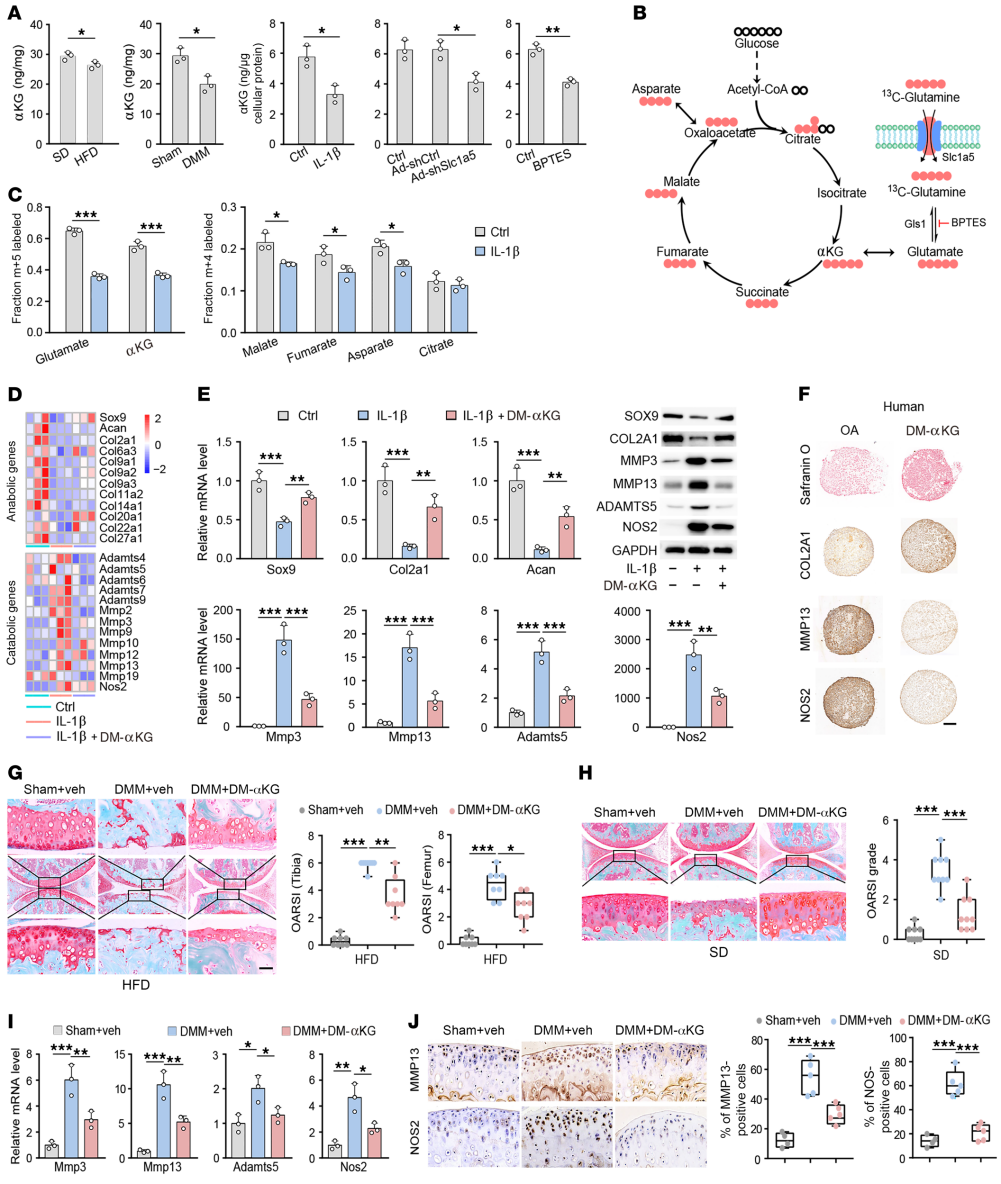
αKG protected against cartilage destruction by restoration of anabolic and catabolic factors. (**A**) αKG level in chondrocytes stimulated with IL-1β for 36 hours and infected with Ad-shCtrl or Ad-sh*Slc1a5* or treated with BPTES (20 μM) for 72 hours. Knee cartilage of mice fed a standard diet (SD) or an HFD, as well as in sham- or DMM-operated knee cartilage. (**B**) Metabolic map showing uniformly labeled Gln (U-^13C^) generating metabolites associated with the TCA cycle. (**C**) The fractional enrichment of the ^13^C isotopologs in each metabolite as determined by LC-MS analysis in chondrocytes treated with IL-1β for 6 hours. (**D**) Heatmap showing differentially expressed anabolic and catabolic genes regulated by DM-αKG supplementation (7 mM) in chondrocytes treated with IL-1β for 24 hours by RNA-seq. (**E**) Quantitative analysis of qRT-PCR (*n* = 3 per group) and Western blot analysis for the indicated anabolic and catabolic factors regulated by αKG supplementation in IL-1β–treated chondrocytes. (**F**) Safranin O staining and IHC staining for COL2A1, MMP13, and NOS2 of the indicated anabolic and catabolic factors in pellet cultures of human OA chondrocytes treated with or without DM-αKG for 21 days. Scale bars: 200 μm. (**G**) Safranin O/fast green staining and OARSI scores of knee joints at 6 weeks after sham or DMM surgery in HFD mice. (**H**) Representative images of safranin O/fast green staining and OARSI scores in mouse knee joints at 8 weeks after sham or DMM surgery. (**I**) qRT-PCR of Mmp3, Mmp13, Adamts5, and Nos2 in cartilage injected with vehicle (veh) or DM-αKG. (**J**) IHC staining for MMP13 and NOS2 and quantification of MMP13- and NOS2-positive cells in mouse knee joints injected with veh or DM-αKG. The data are presented as box plots or as the mean ± SEM, and the dots represent biological replicates. *P < 0.05, **P < 0.01, ***P < 0.001. Ctrl, control.

The metabolite αKG represents the major downstream product of glutaminolysis, which could enter the TCA cycle (24). Levels of αKG were markedly reduced in cartilage of obese and DMM mice, as well as in IL-1β–treated chondrocytes, after SLC1A5 blockade or GLS1 inhibition (Figure 4A). Using gas chromatography–mass spectrometry to trace the fate of uniformly ^13^C-labeled Gln, we found that approximately 55% of αKG was derived from ^13^C-Gln. Labeled carbons in Glu (m+5) and αKG (m+5) were decreased by IL-1β, along with reductions in fumarate (m+4), malate (m+4), and aspartate (m+4), which were partly derived from ^13^C-Gln. These reductions indicated decreased generation of αKG from glutaminolysis in OA chondrocytes (Figure 4, B and C, and Supplemental Figure 5A).

Because key upstream modulators of glutaminolysis were suppressed under the inflammatory milieu, the potential effect of supplementation of dimethyl-αKG (DM-αKG), a cell-permeable analog of αKG, to rescue the anabolic/catabolic imbalance caused by impaired glutaminolysis was investigated. Consistent with expectations, supplementation of DM-αKG substantially reversed the expression of catabolic factors and anabolic factors in GPNA- or BPTES-treated chondrocytes (Supplemental Figure 6, A and B). Moreover, RNA-seq analysis, quantitative PCR (qPCR), and Western blotting confirmed the reversal of IL-1β–induced alterations in the anabolism/catabolism balance by DM-αKG (Figure 4, D and E, and Supplemental Figure 6, C–E). Furthermore, onetime intra-articular injection of DM-αKG resulted in marked and sustained increases in αKG levels in articular cartilage for 7 days (Supplemental Figure 7A). Therefore, weekly intra-articular injections of DM-αKG (0.7 M) were applied starting at 3 weeks after DMM surgery and were continued for 5 consecutive weeks. This approach markedly attenuated cartilage destruction, substantially improved OARSI scores in both obese and lean mice (Figure 4, G and H), and decreased percentages of MMP13- and NOS2-positive cells as well as corresponding mRNA levels (Figure 4, I and J). The protective effect of DM-αKG was dose dependent (Supplemental Figure 7B) and was sustained for up to 12 weeks after surgery (Supplemental Figure 7C). In pellet cultures generated from human OA chondrocytes, DM-αKG also markedly upregulated COL2A1 and reduced MMP13 and NOS2 signatures in IHC assays (Figure 4F and Supplemental Figure 7D). Together, these results demonstrated that DM-αKG supplementation rescued the anabolic/catabolic imbalance in OA chondrocytes and exerted a protective effect against the cartilage destruction.

To determine whether the rescuing effect of αKG on anabolism/catabolism imbalance is mediated by replenishing the TCA cycle, succinyl phosphonate trisodium salt (200 μM) was used to inhibit αKG dehydrogenase, which catalyzes conversion of αKG into succinate in the TCA cycle. This approach elicited limited, if any, effects on the expression of anabolic and catabolic factors examined in normal chondrocytes (Supplemental Figure 8, A and B). In the presence of IL-1β, TCA blockade did lead to a mild increase in anabolic/catabolic imbalance (Supplemental Figure 8, A and B). However, treatment of chondrocytes with diethyl succinate, a cell-permeable analog of succinate, failed to rescue IL-1β–induced metabolic imbalance (Supplemental Figure 8, C and D), suggesting that αKG does not exert its rescue effects via entering the TCA cycle. In addition, αKG also has been reported to serve as an essential cofactor for prolyl hydroxylases, enzymes that regulate HIF-1α stability (25). In fact, DM-αKG supplementation induced dose-dependent HIF-1α accumulation in both cytosolic and nuclear fractions in normal chondrocytes (Supplemental Figure 9, A and B). However, co-treatment with PX-478 (40 μM), an inhibitor of HIF-1α, did not abrogate the chondroprotective effects of DM-αKG (Supplemental Figure 9, C and D). These data collectively demonstrate that αKG-mediated chondroprotection operates in a TCA cycle– and HIF-1α–independent manner.

### DM-αKG restored glycolysis and oxidative phosphorylation reprogramming in OA chondrocytes.

**Figure 5:**
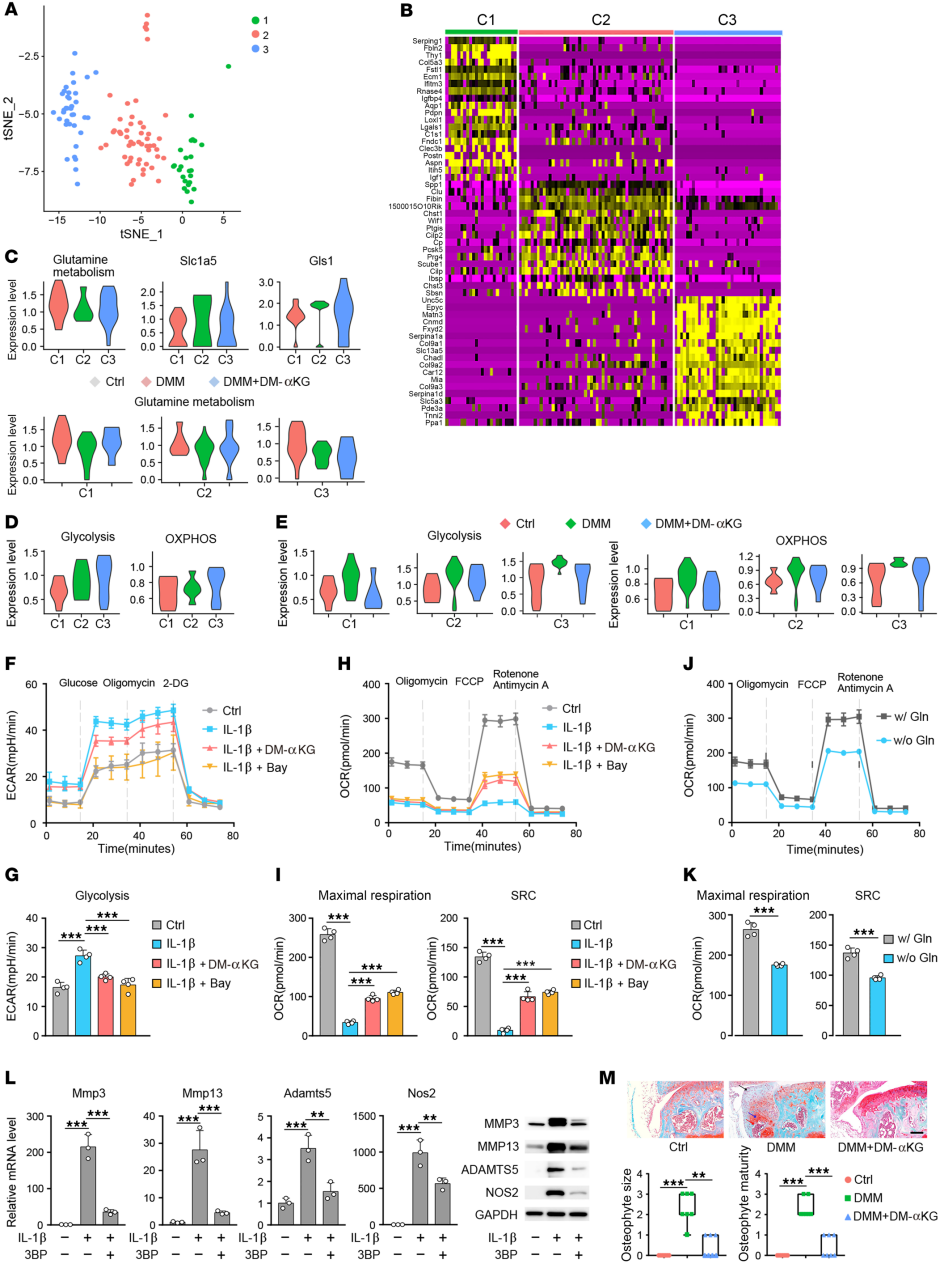
Supplementation of DM-αKG restored metabolic reprogramming in OA chondrocytes. (**A**) Three clusters of chondrocytes sourced from the medial tibial condyle were identified with a t-distributed stochastic neighbor embedding (t-SNE) plot. (**B**) Heatmap revealing the top 20 substantially differentially expressed genes in each cluster. (**C**) The expression levels of genes associated with Gln metabolism in the C1, C2, and C3 clusters; control; DMM; and DMM+DM-αKG groups of each cluster. (**D**) The expression levels of genes associated with glycolysis and OXPHOS in the C1, C2, and C3 clusters. (**E**) Expression levels of genes associated with glycolysis and OXPHOS in the control, DMM, and DMM+DM-αKG groups of each cluster. (**F**) ECAR of chondrocytes treated with IL-1β alone, IL-1β+ DM-αKG, and combined IL-1β and Bay-11-7085, as measured by the Seahorse Analyzer. (**G**) Quantification of glycolysis from 1 time point in the glycolysis stress test. (**H**) OCR of chondrocytes treated with IL-1β, IL-1β, and DM-αKG in combination, combined IL-1β and Bay-11-7085, as measured by the Seahorse Analyzer. (**I**) Quantification of maximal respiration and spare respiratory capacity (SRC) for 1 time point each from Mito Stress test. (**J**) Chondrocytes were cultured for 24 hours with or without Gln, and the OCR was measured using a Seahorse XF96 analyzer. (**K**) Quantification of maximal respiration and SRC for 1 time point each from Mito Stress test. (**L**) RT-PCR and Western blotting of the indicated anabolic and catabolic factors regulated by 3-bromopyruvate (3BP) supplementation in IL-1β–treated chondrocytes for 24 hours. Blots are representative of 3 independent experiments. (**M**) Safranin O staining and scoring of osteophyte size and osteophyte maturity at 8 weeks after DMM surgery. Black arrow indicates fibrocartilage, blue arrow indicates neocartilage formation, and red arrow indicates ossification. Scale bars: 50 μm. The data are presented as the mean ± SEM, and the dots represent biological replicates. **P < 0.01, ***P < 0.001. Ctrl, control; FCCP, carbonyl cyanide *p*-trifluoromethoxyphenylhydrazone; w/, with; w/o, without.

Although the heterogeneity of articular chondrocytes was well documented in our previous study as well as by others (26–28), alterations in Gln metabolism within specific subsets of OA chondrocytes remain poorly understood. Furthermore, the mechanism by which αKG restores the anabolism/catabolism balance has not been fully elucidated. To this end, we performed single-cell RNA-seq (scRNA-seq) on cells isolated from the medial tibial condyles of normal, DMM, and DM-αKG–rescued mice. Among the 7 clusters identified (Supplemental Figure 10, A and B), the one with high expression of Sox9, Acan, Comp, and Fn was characterized as a chondrocyte population (Supplemental Figure 10C) that could be subsequently subdivided into superficial-zone (C1), middle-zone (C2), and deep-zone (C3) subsets (Figure 5, A and B) and was validated by immunofluorescence staining (Supplemental Figure 10D). Gene Ontology (GO) analysis revealed that each cluster was enriched with featured gene profiles (Supplemental Figure 10E). We further performed GO analysis to elucidate the transcriptomic changes in anabolism and catabolism of each chondrocyte subset from control, DMM, and DM-αKG–supplemented DMM mice. The genes that were upregulated by DMM were related to ossification, growth factor binding, and extracellular matrix organization, which were generally decreased by DM-αKG in all 3 subsets (Supplemental Figure 11A). Data from scRNA-seq clearly displayed Gln metabolism with comparable expression of Slc1a5 and Gls1 within each subset of chondrocytes (Figure 5C). Consistent with in vitro and in vivo results, scRNA-seq showed that the overall Gln metabolism was reduced in all 3 DMM subsets (Figure 5C). These results suggested that chondrocytes undergo a degree of Gln metabolism in heathy cartilage, and all 3 subsets displayed impairment in Gln metabolism in OA cartilage.

Given that alterations in glycolysis have been linked to enhanced catabolic activity in OA chondrocytes, it is interesting to find by scRNA-seq that glycolysis was increased in all 3 DMM clusters (Figure 5, D and E, and Supplemental Figure 11B), and this was evidenced by enhanced extracellular acidification rate (ECAR) and glycolysis in IL-1β–treated chondrocytes (Figure 5, F and G). Indeed, we found that inhibiting glycolysis by 3-bromopyruvate, a potent hexokinase II inhibitor, led to marked reduction in catabolic factors (Figure 5L). In contrast with scRNA-seq data, which showed an overall increase of oxidative phosphorylation (OXPHOS), in vitro studies revealed that OXPHOS was decreased upon IL-1β stimulation (Figure 5, H–K). This discrepancy could be due to the cartilage at late-stage OA being harvested for scRNA-seq, where OXPHOS was required for supporting fibrosis and ossification (29), and this was evidenced by elevated enrichment for angiogenesis and ossification and by histology showing osteophyte formation (Figure 5M and Supplemental Figure 11, C–F) in the DMM joint. Yet, DM-αKG supplementation could restore, in part, the reprogrammed glycolysis and the maximal and spare respiratory capacity in OXPHOS in OA chondrocytes, respectively (Figure 5, E–I). Together, these results revealed that dysregulation of Gln metabolism occurs in all 3 chondrocyte subsets in OA cartilage, with accompanied glycolysis and OXPHOS reprogramming.

### Epigenetic inhibition of glutaminolysis genes via H3K27me3 methylation is regulated by αKG availability.

After treatment with DM-αKG, a partial recovery of Gln metabolism was observed in DMM mice (Supplemental Figure 12E). Given that JHDMs depend on αKG as a cofactor for H3K27me3 demethylation, and considering the documented association between H3K27me3 and OA progression (19, 30), we explored whether histone modifications contribute to OA pathogenesis by repressing key glutaminolysis genes. Treatment with EPZ005687, an inhibitor of the H3K27 methyltransferase *Ezh2*, reduced H3K27me3 levels and markedly increased the expression of *Slc1a5* and *Gls1* in chondrocytes at both mRNA and protein levels (Supplemental Figure 12, A and B). Furthermore, EPZ005687 counteracted the IL-1β–induced reduction in intracellular Gln and αKG levels, as well as the elevation in H3K27me3 modification (Supplemental Figure 12C). ChIP assays further confirmed enhanced H3K27me3 enrichment at the promoter regions of *Slc1a5* and *Gls1* after IL-1β stimulation (Supplemental Figure 12D), supporting epigenetic repression of these genes.

We next examined the role of αKG in restoring glutaminolysis. Supplementation with DM-αKG attenuated H3K27me3 accumulation and restored the expression of *Slc1a5* and *Gls1* (Supplemental Figure 12, E–G). Importantly, the demethylase inhibitor GSK-J4 abrogated the rescue effects mediated by DM-αKG (Supplemental Figure 12, F and G), confirming the dependence on JHDM activity. Collectively, these data demonstrate that inflammatory cytokine–induced epigenetic reprogramming through H3K27me3 hypermethylation directly suppresses *Slc1a5* and *Gls1* expression and that this repression is dynamically regulated by αKG-dependent histone demethylation.

### UBE2O induced by αKG is mediated by histone H3K27me3 demethylation.

**Figure 6:**
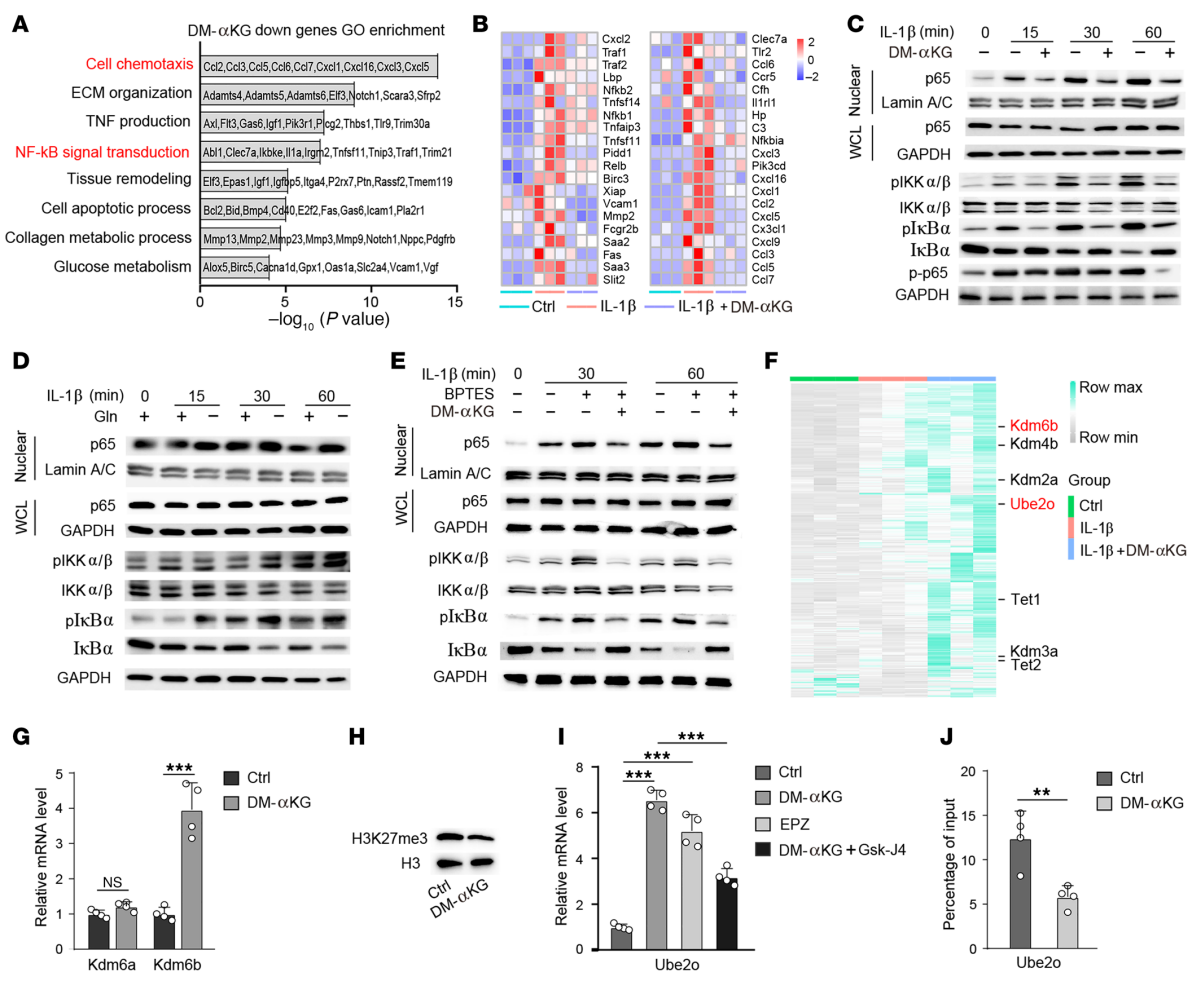
αKG-induced Ube2o is mediated by histone methylation on H3K27me3. (**A**) GO enrichment analysis of the categories of downregulated genes regulated by αKG supplementation in IL-1β–treated chondrocytes. (**B**) Heatmap of NF-κB pathway genes downregulated by DM-αKG that were upregulated by IL-1β in chondrocytes. *n* = 3 per group. Western blot analysis of p65, IKKα/β, p-IKKα/β, IκBα, and p-IκBα in IL-1β–treated chondrocytes (**C**) supplemented with DM-αKG and (**D**) deprived of Gln, respectively. Blots are representative of 3 independent experiments. WCL, whole-cell lysate. (**E**) Western blot analysis of p65, IKKα/β, p-IKKα/β, IκBα, and p-IκBα in chondrocytes stimulated with IL-1β in medium with or without BPTES or supplemented with vehicle (control [ctrl]) or DM-αKG (7 mM). Blots are representative of 3 independent experiments. (**F**) Heatmap of differentially expressed genes in control, IL-1β–, and DM-αKG–supplemented (7 mM) chondrocytes for 24 hours by RNA-seq (*n* = 3 per group). (**G**) qRT-PCR analyses of Kdm6a and Kdm6b in chondrocytes after supplementation of αKG for 6H (*n* = 4 per group). (**H**) Western blot analyses of H3K27me3 in chondrocytes after supplementation of αKG for 6 hours. Blots are representative of 3 independent experiments. (**I**) qRT-PCR (*n* = 4 per group) of UBE2O in DM-αKG– and Gsk-J4–treated chondrocytes for 6 hours. (**J**) ChIP-qPCR showed that αKG decreased the occupancy of H3K27me3 in the promoter regions of UBE2O (*n* = 4 per group). The data are presented as the mean ± SEM, and the dots represent biological replicates. **P < 0.01, ***P < 0.001. ECM, extracellular matrix; max, maximum; min, minimum.

Notably, inhibition of the NF-κB pathway using Bay11-7085, an inhibitor of IκBα phosphorylation, restored both glycolysis and OXPHOS to an extent comparable to that of αKG supplementation (Figure 5, E–I), prompting us to investigate whether αKG ameliorates metabolic reprogramming via suppression of NF-κB signaling. Using RNA-seq analysis and Western blot, we discovered that NF-κB activation induced by IL-1β, including nuclear translocation of p65, as well as phosphorylation of IκB kinase-α and IκB kinase-β (IKKα/β), IκBα, and p65, were markedly inhibited by DM-αKG (Figure 6, A–E, and Supplemental Figure 13A).

Among the genes that were upregulated by αKG, a gene encoding the E2 ubiquitin-conjugating enzyme protein, *Ube2o*, was noteworthy because *Ube2o* is a putative negative regulator of NF-κB signaling (Figure 6F). The epigenetic role of αKG is well characterized; it acts as a cofactor for JHDMs in modulating gene expression. The expression levels and activities of the HDM Kdm6b, an H3K27me3 demethylase, were also found to be modified by αKG (Figure 6, F and G). As expected, αKG-treated chondrocytes had reduced levels of H3K27me3 methylation (Figure 6H).

The induction of *Ube2o* was hypothesized to result from *Kdm6b*-mediated epigenetic modification. To test this, chondrocytes were treated with Gsk-J4, an inhibitor that targets Kdm6b, along with αKG treatment. Indeed, *Ube2o* expression was induced by αKG and was reduced in Gsk-J4-treated chondrocytes (Figure 6I), further supporting the mechanistic role of H3K27me3 demethylation in the chondroprotective effects of αKG.

### UBE2O expression induced by αKG inhibited TNF receptor associated factor 6 ubiquitination and downstream NF-κB signaling in OA chondrocytes.

**Figure 7:**
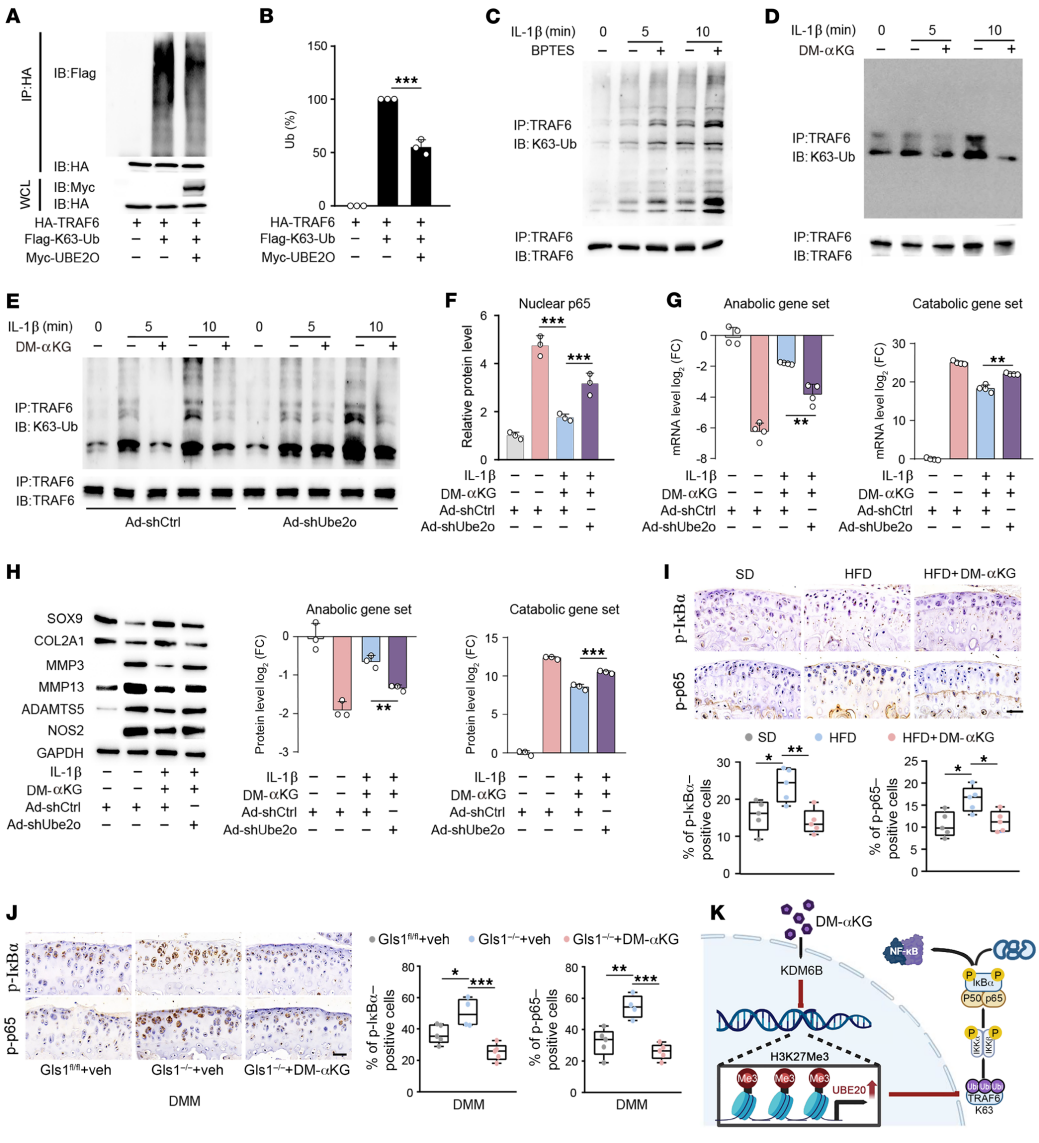
αKG inhibited the TRAF6 ubiquitination via inducing the expression of UBE2O in OA chondrocytes. (**A**) IB analysis of K63-linked ubiquitination of TRAF6 in HEK293T cells transfected to express HA-TRAF6 with or without Flag-tagged K63-linked ubiquitin (Flag-K63-Ub) and MYC-tagged UBE2O (MYC-UBE2O). (**B**) Densitometry of the bands in **A**, showing the ubiquitination (Ub) of TRAF6, presented relative to results obtained with cells transfected to express empty vector, set as 100%. (**C**) Western blot analysis of K63-linked ubiquitination of endogenous TRAF6 in IL-1β–stimulated chondrocytes in the presence of BPTES. Blots are representative of 3 independent experiments. (**D**) Western blot analysis of K63-linked ubiquitination of endogenous TRAF6 that was immunoprecipitated and TRAF6 from IL-1β– and DM-αKG–stimulated chondrocytes. (**E**) Western blot analysis of K63-linked ubiquitination of endogenous TRAF6 immunoprecipitated from Ad-shCtrl and Ad-shUbe2o chondrocytes stimulated with IL-1β and DM-αKG. (**F**) Protein quantitative analysis of the nuclear p65 from Ad-shCtrl and Ad-shUbe2o chondrocytes stimulated with IL-1β and DM-αKG for 60 minutes. (**G** and **H**) Quantitative analysis of qRT-PCR (**G**) and Western blot (**H**) analysis for the indicated anabolic and catabolic factors from Ad-shCtrl and Ad-shUbe2o chondrocytes stimulated with IL-1β and DM-αKG. (**I**) IHC staining for pIκBα, p-p65, and quantification of pIκBα- and p-p65–positive cells at 25 weeks in mice fed a standard diet (SD) or HFD. (**J**) IHC staining for pIκBα, p-p65, and quantification of pIκBα- and p-p65–positive cells in Gls1^−/−^ mice and their Gls1^fl/fl^ control littermates after DMM surgery. veh, vehicle. (**K**) Schematic depicting the described findings: αKG inhibited the TRAF6 ubiquitination via inducing the expression of UBE2O in OA chondrocytes. The data are presented as box plots or as the mean ± SEM, and the dots represent biological replicates. *P < 0.05, **P < 0.01, ***P < 0.001.

The results here revealed a marked decrease in H3K27me3 levels in chondrocytes upon DM-αKG treatment. Furthermore, ChIP analysis using an antibody against H3K27me3 demonstrated a decrease in H3K27me3 at the promoter region of *Ube2o* after αKG treatment (Figure 6J). The E2 ubiquitin–conjugating enzyme *Ube2o* functions as a negative regulator of TNF receptor associated factor 6–mediated (TRAF6-mediated) NF-κB activation by impeding TRAF6 polyubiquitination (31). Transfection of Myc-tagged *Ube2o* in HEK293T cells with or without HA-tagged TRAF6 and Flag-tagged K63-Ub demonstrated that *Ube2o* overexpression resulted in a substantial decrease in TRAF6 ubiquitin levels (Figure 7, A and B). Gln metabolic deficiency generated by treating the chondrocytes with BPTES (20 μM) (a *Gls1* inhibitor), l-methionine-dl-sulfoximine (60 μM; a Gln synthetase [GS] inhibitor), or R162 (20 μM; a *Glud1* inhibitor) further enhanced IL-1β–induced TRAF6 ubiquitination (Figure 7C and Supplemental Figure 13B). By coimmunoprecipitation assays, DM-αKG supplementation reduced IL-1β–induced K63-linked ubiquitination of TRAF6 in chondrocytes (Figure 7D).

To further address the role of αKG on TRAF6 ubiquitylation, we transiently transfected HEK293T cells with plasmids encoding HA-tagged TRAF6 and Flag-tagged K63-Ub. These transfected cells demonstrated a decrease in the abundance of Flag-K63-Ub upon DM-αKG treatment (Supplemental Figure 13C). Knockdown of UBE2O by shRNA reversed the suppression of TRAF6 ubiquitination by DM-αKG in IL-1β–treated chondrocytes (Figure 7E), as well as TRAF6 downstream nuclear p65 (Figure 7F and Supplemental Figure 13D), indicating that *Ube2o* deficiency resulted in insensitivity to DM-αKG supplementation. As expected, *Ube2o* knockdown partly reverted the beneficial effects of DM-αKG on the anabolic/catabolic balance (Figure 7, G and H; Supplemental Figure 13, G and H; and Supplemental Figure 14, A and B), suggesting *Ube2o* is a major downstream mediator for DM-αKG.

In vivo, the percentages of phospho-IκBα–positive (p-IκBα–positive) and p-p65–positive cells in obese mice (Gln deficient) were higher than in lean mice (Figure 7I). More importantly, intra-articular administration of DM-αKG substantially decreased the percentages of cells positive for phosphorylation of IκBα and p65 in obese mice and DMM cartilage (Figure 7I and Supplemental Figure 13F). Concurring with previous results, *Gls1*-cKO mice exhibited worsened pathology in DMM joint cartilage compared with *Gls1*^fl/fl^ control mice. Accordingly, the percentages of cells positive for phosphorylated IκBα and p65 in DMM cartilage were also elevated substantially in *Gls1*-cKO mice, and DM-αKG administration markedly reduced cells with activated NF-κB signaling (Figure 7J). Cumulatively, these results demonstrated that αKG inhibited NF-κB signaling by suppressing TRAF6 ubiquitination via induction of *Ube2o* expression (Figure 7K).

## Discussion

In this study, impaired glutaminolysis was a consistent feature in OA cartilage under obesity-related and injury-induced conditions in vivo as well as in chondrocytes treated with IL-1β in vitro, resulting in NF-κB activation and an imbalance between anabolism and catabolism. OA pathogenic factors lead to impairment in Gln metabolism through increased H3K27me3 epigenetic modification of key glutaminolysis genes. Due to downregulation of a Gln transporter, *Slc1a5*, extracellular supplementation of Gln failed to rescue cartilage degradation, whereas supplementation of DM-αKG, which could directly enter chondrocytes, protected against cartilage destruction by epigenetically inducing not only key glutaminolysis genes but also *Ube2o* to suppress K63-linked ubiquitination of TRAF6, leading to inhibition of the NF-κB pathway and OA relief.

Under physiological circumstances, Gln is an amide-group donor for the biosynthesis of glycosaminoglycan in articular cartilage (32). During endochondral bone morphogenesis, Gln acts as a crucial metabolic regulator supporting growth and biosynthesis of chondrocytes (15). Gln metabolism was impaired in both human and mouse obesity-related or injury-induced OA cartilage. Notably, Gln deficiency resulting from inflammatory stimulation has been linked with several pathological conditions, such as inflammatory bowel disease and white adipose tissue inflammation (23). Here, key modulators that control Gln metabolism, including *Slc1a5* and *Gls1*, were substantially inhibited by the inflammatory cytokine IL-1β through increased promoter deposition of H3K27me3, attenuating the transportation of extracellular Gln as well as generation of Glu from Gln. Results from this study also demonstrated that supplementation of exogenous Gln, which has been proposed as a potential treatment to alleviate OA pathology (33), elicited minimal effects on rescuing impaired glutaminolysis or mitigating catabolic activities in IL-1β–treated chondrocytes, due to reduced Gln transporter expression.

Although our data show that Gln levels are reduced within OA cartilage, it is important to note that exogenous Gln supplementation did not rescue the impaired anabolic/catabolic balance in IL-1β–treated chondrocytes. This suggests the defect lies not in the general availability of Gln but more critically in the chondrocyte’s capacity to import and metabolize it under inflammatory conditions. In line with this, we and others have observed that synovial-fluid Gln levels can be elevated in OA (34). This apparent paradox can be explained by our finding that key modulators of Gln uptake and metabolism *Slc1a5* and *Gls1* are downregulated in chondrocytes by IL-1β. Mechanistically, we discovered that this downregulation is associated with an increase in the repressive histone mark H3K27me3 at the promoters of these metabolic genes, indicating an epigenetic silencing mechanism. This failure of chondrocytes to efficiently clear Gln from the extracellular space, due to epigenetically suppressed transporters, likely contributes to its accumulation in the synovial fluid, while simultaneously creating an intracellular deficiency within the cartilage. This metabolic impairment, once established, creates intracellular Gln deficiency, further amplifying NF-κB signaling and leading to sustained inflammation and cartilage breakdown. Thus, the OA-associated decline in cartilage Gln levels reflects a profound and potentially long-lasting chondrocyte-intrinsic failure to use Gln, orchestrated by both transcriptional and epigenetic mechanisms.

Activation of NF-κB signaling is well known to play a central role in orchestrating the conversion of pathological stimulus into catabolic reactions in OA cartilage (35, 36). We showed that Gln deficiency boosted the activation of NF-κB signaling in IL-1β–treated chondrocytes. In vivo, *Gls1*-cKO mice had a higher percentage of p-IκBα– and p-p65–positive cells in DMM joint cartilage than did their control littermates. Intriguingly, Gln deprivation alone could induce expression of IL-1β in chondrocytes. In fact, it has been reported that Gln deprivation could trigger the activation of NF-κB signaling and subsequent expression of pro-inflammatory factors (37), but the mechanism remains unknown. DM-αKG has been implicated in impeding IKK activation by augmenting prolyl hydroxylase activity to attenuate the NF-κB pathway (38). However, in this study, treatment with dimethyloxalyglycine, an inhibitor of prolyl hydroxylase, showed no rescue of NF-κB signaling suppressed by DM-αKG (Supplemental Figure 13E), suggesting other mechanisms are involved. Among TRAFs, only TRAF6 mediates IL-1R/TLR signaling. After lysine 63 (K63) auto-polyubiquitination of TRAF6, the cascade of events including activation of the IKK complex and subsequent degradation of NF-κB inhibitor IκBα was triggered, enabling nuclear translocation of the NF-κB complex to initiate expression of a broad range of inflammatory cytokines (39). In this study, IL-1β–induced TRAF6 ubiquitination increased upon the blockage of Gln metabolism by *Gls1* and *Glud1* inhibitors. Thus, with enhanced TRAF6 ubiquitination under conditions of impaired Gln metabolism, NF-κB activation was further boosted in IL-1β–treated chondrocytes, exacerbating the anabolic/catabolic imbalance in OA cartilage. This paradigm was also evident in obese mice, which had reduced Gln levels and elevated NF-κB activation in joint cartilage, effecting more severe cartilage destruction compared with their lean control counterparts after DMM surgery.

Control of TRAF6 activity relies largely on K63 auto-polyubiquitination, which can be antagonized by *Ube2o*, an enzyme previously described to be a negative regulator of NF-κB signaling, via disrupting the formation of the MyD88-TRAF6 protein complex (31). Here, with minimal involvement from the TCA cycle, DM-αKG epigenetically increased *Ube2o* expression by activation of a histone H3K27 demethylase, *Kdm6b*, inhibiting TRAF6 ubiquitination and subsequent NF-κB activation. Through inhibition of upstream TRAF6 ubiquitination and the subsequent NF-κB signaling cascade, the balance of anabolism and catabolism was restored by DM-αKG supplementation in OA chondrocytes. More importantly, by decreasing NF-κB activation, DM-αKG administration effectively rescued the cartilage degradation by reversing the anabolic/catabolic imbalance in chondrocytes even under deregulated glutaminolysis conditions, as was the case in *Gls*-cKO and obese mice.

Multiple immune cell types, including macrophages, neutrophils, B cells, and T cells, have been implicated in OA pathogenesis (40, 41). For instance, neutrophils are a major source of inflammatory cytokines and matrix-degrading enzymes (Mmp8, Mmp9, Adam8), and their sustained presence in the joint after injury may substantially exacerbate OA progression. B cells have also been reported to upregulate cytokine and protease expression after injury, further contributing to the disease process. In our study, the tissue samples included articular cartilage, subchondral bone, and bone marrow. scRNA-seq identified 7 major cell clusters, chondrocytes, neutrophils, monocytes, B lymphocytes, T lymphocytes, erythrocytes, and NK cells. Notably, besides the chondrocyte population, we observed a marked increase in neutrophils at 8 weeks after DMM surgery, which was effectively reversed by intra-articular αKG administration. Given the reported role of neutrophils in promoting pathological neovascularization in damaged tissues, we speculate that their accumulation may be linked to osteophyte formation during OA development (42). The potential roles of neutrophils and other immune cells in OA represent an exciting direction for research.

In conclusion, this study revealed a reciprocal interaction of glutaminolysis and inflammatory pathway activation in chondrocytes that contribute to the onset and development of OA pathology. The epigenetic modification H3K27me3 appears to act as the fulcrum for the counterbalancing activities of histone methyltransferase *Ezh2* and histone demethylase *Kdm6b*, which respectively down- and upregulate expression of *Slc1a5/Gls1* to influence glutaminolysis as well as a ubiquitin E2 ligase gene, *Ube2o*, which, once elevated, inhibits Traf6-polyubiquitination-dependent IL-1β/NF-κB signaling. Moreover, this work highlights the protective function of DM-αKG, a cofactor for *Kdm6b*, against cartilage destruction. Taken together, glutaminolysis and αKG, an important epigenetic cofactor, could ultimately be prominent therapeutic targets for perhaps not only OA but also other degenerative conditions related to Gln deficiency, including obesity and aging.

## Methods

### Sex as a biological variable.

Our study incorporated clinical samples from both male and female donors. To establish a controlled experimental model, we used male mice for the in vivo and ex vivo investigations described herein. Because the design did not include a comparison between sexes, sex was not treated as an experimental variable. We note that the potential applicability of the mechanistic findings to female mice requires further validation.

### Human specimens.

Human articular cartilage samples were obtained from patients with OA undergoing total knee arthroplasty. All cartilage was obtained within 2 hours after arthroplasty. The specimens were processed for qPCR and histological examination. For metabolomic analysis, the cartilage was placed on dry ice immediately after being harvested. Damage severity was determined according to ICRS grading scores (43). The patients with OA (*n* = 3 men and 14 women) had a median age of 66 years.

### Mice.

Floxed Gls1 mice (44) (catalog 017894) and Col2a1-CreERT2 mice (45) (catalog 006774) were purchased from The Jackson Laboratory. To generate Gls1-cKO mice, Col2a1-Cre transgenic mice were crossed with Gls1^fl/fl^ mice, generating the following 3 genotypes: Gls1 cKO, Gls1^fl/+^ Col2a1-Cre, and Gls1^fl/fl^. The genotypes of the transgenic mice were determined by PCR analysis of genomic DNA isolated from mouse tail snips. Recombinase activity in chondrocytes of postnatal Col2-Cre^ERT2^ mice was driven by tamoxifen (1 mg/10 g body weight; Sigma), which was administered via intraperitoneal injections to 7-week-old mice for 7 consecutive days. All mice were housed in a specific pathogen–free animal facility with a 12-hour light/12-hour dark cycle. The mice had free access to food and water.

The surgery for DMM was performed on the right knee joints of 10-week-old male C57BL/6 mice (46); sham-operated mice were used as controls. To knock down Slc1a5 in chondrocytes, sham- or DMM-operated mice were given intra-articular injections of recombinant Ad-shSlc1a5; injection of empty adenovirus (Ad-shCtrl) was a control. Intra-articular injection of recombinant adenoviruses was chosen for in vivo overexpression of Slc1a5 and Gls1. A total of 1 × 10^9^ particles in a 10 μL volume were injected into the knee joint cavity once per week for 3 weeks beginning at 10 days after surgery. All adenoviruses were purchased from Wei Nuo Sai Biology. In the DM-αKG treatment group, mice received PBS or DM-αKG (0.7 M) by intra-articular injection into the knee once per week for 5 weeks beginning at 3 weeks after surgery.

For the HFD experiment, beginning at 4 weeks of age, mice were fed a diet of which 60% of kilocalories were from fat. DMM or sham surgery was performed on the right knees of mice after 20 weeks of HFD feeding, and the mice were sacrificed 6 weeks after surgery for histological evaluation. The mice used for all experiments were randomly assigned to control or treatment groups and randomly selected for OA analysis.

### Cell isolation and culture.

Primary chondrocytes were isolated from the articular cartilage of 7-day-old C57BL/6J mice and digested with 0.2% NB4 collagenase. Chondrocytes were collected by centrifugation at 1,000*g* and resuspended in DMEM containing 10% FBS. For Gln deprivation experiments, Gln-free DMEM was used. Human articular chondrocytes were harvested from knee joints of patients with OA by digestion with 0.2% NB4 collagenase. For 3D pellet culture of human OA chondrocytes, 400,000 cells at passage 1 were seeded in a round-bottom, 96-well plate. After 21 days, pellets were collected and subjected to histological analysis. Cells were treated with GPNA (Sigma, G6133), BPTES (Sigma, SML0601), dimethyl-αKG (Sigma, 349631), 3-bromopyruvate (MCE, HY-19992), antimycin A (Agilent, 103015-100), or DMSO as vehicle control in indicated experiments.

### Adenovirus infection of chondrocytes.

Recombinant adenoviruses expressing mouse Slc1a5 (Ad-Slc1a5) and Gls1 (Ad-Gls1) were purchased from Wei Nuo Sai Biology. Knockdown of Slc1a5 was performed using adenoviral vectors carrying Slc1a5 shRNA (Ad-shSlc1a5) (sequence: 5′-GGTCCTGGTCTCCTGGATTAT-3′), which were generated by Wei Nuo Sai Biology. Scrambled shRNA was used as a control. Adenovirus packaging was performed by transfecting HEK293T cells with the recombinant adenoviral vector. The adenoviruses were harvested from the AAV-293 cell supernatant, condensed, and purified for further experiments. Prior to infection, mouse articular chondrocytes were allowed to grow for 2 days, then were infected with adenoviruses for 2 hours at an MOI of 800 and cultured for 48 hours before further analysis.

### Measurement of intracellular Gln and αKG in chondrocytes.

Primary cultured chondrocytes were treated with or without IL-1β (5 ng/mL) for 6, 12, 24, and 36 hours, and the levels of intracellular Gln and αKG were measured with an EnzyChrom Gln Assay Kit (BioAssay Systems, EGLN-100) and α-Ketoglutarate Assay Kit (Sigma, MAK054), respectively, according to the manufacturer’s instructions. Detection of intracellular αKG level in cartilage was performed by harvesting articular cartilage from the knee joint at 1, 3, 5, and 7 days after DM-αKG injection. Chondrocytes were collected by digestion with 0.2% NB4 collagenase for 6 hours at 37°C, and the level of αKG was measured using the α-Ketoglutarate Assay Kit.

### Quantitative real-time PCR.

Total RNA was extracted from cartilage tissue and primary cultures of articular chondrocytes using TRIzol reagent (Invitrogen) according to the manufacturer’s instructions. The RNA was reverse-transcribed into cDNA using a PrimeScript RT Reagent Kit (Takara). qPCR was performed using a SYBR PrimeScript RT-PCR Kit (Takara), and the reaction mixtures were incubated at 95°C for 1 minute followed by 40 cycles of 95°C for 15 seconds and 60°C for 1 minute, using an ABI 7500 System (Applied Biosystems). β-Actin served as a housekeeping gene. The relative gene expression was calculated by the comparative Ct method. The sequences of the primers used are listed in Supplemental Table 1.

### Western blot.

Chondrocytes were lysed in RIPA buffer containing a protease-inhibitor cocktail. The nuclear protein was extracted using a Nuclear Protein Extraction Kit (BiYunTian, P0027). Concentration of the collected protein was determined with a BCA protein assay kit (BiYunTian). Extracted proteins were size fractionated by SDS-PAGE and transferred to PVDF membranes, with subsequent blocking with 5% skim milk powder. The membranes were incubated overnight at 4°C with primary antibodies against SOX9 (Abcam, ab3697; 1:1,000); Collagen II (Abcam, ab85266; 1:1,000); MMP3 (Abcam, ab52915; 1:1,000); MMP13 (Proteintech, 18165-1-AP; 1:1,000); ADAMTS5 (Abcam, ab41037; 1:200); NOS2 (Abcam, ab178945; 1:1,000); SLC1A5 (Sigma, HPA035239; 1:1,000); GLS1 (Proteintech; 12855-1-ap; 1:1,000); GS (Abcam, ab176562; 1:1,000); NF-κB p65 (Santa Cruz Biotechnology; sc-8008; 1:1,000); lamin A/C (Santa Cruz Biotechnology; sc-6215; 1:5,000); phospho-IKKα/β (Cell Signaling Technology; 2694P; 1:1,000); IKKα/β (Abcam, ab178870; 1:1,000); phospho-IκBα (Affinity, AF2002; 1:1,000); IκBα (Affinity, AF5002; 1:1,000); TRAF6 (Santa Cruz Biotechnology; sc-8409; 1:200); K63 Ub (Cell Signaling Technology; 5621S; 1:1,000); and GAPDH (Abcam, ab8245; 1:2,000). After being washed with PBST, the membranes were incubated with HRP-conjugated secondary antibodies (goat anti–mouse IgG, 1:5,000, and goat anti–rabbit IgG, 1:5,000; 115-035-003 and 111-035-003, respectively, from Jackson Immunoresearch). After 3 washes, protein bands were detected by enhanced chemiluminescence.

### Measurement of cellular metabolism by Seahorse.

Primary chondrocytes were seeded in Seahorse 96-well plates at 40,000 cells per well and allowed to attach overnight. Then, cells were treated with IL-1β in the presence or absence of DM-αKG (7 mM) or NF-κB inhibitor Bay11-7085 (10 μM) for 24 hours. Cells for the oxygen consumption rate (OCR) assay were preincubated in medium with or without 4 mM Gln for 24 hours. The OCR and ECAR were measured by an XF96 Seahorse Extracellular Flux Analyzer, following the manufacturer’s instructions. Measurements in cells treated with glucose, oligomycin, and 2-deoxy-d-glucose were made every 5 minutes for the glycolysis stress test. For the Mito Stress test, measurements were performed every 5 minutes after sequential addition of oligomycin, FCCP, and rotenone/antimycin A. Readings of OCR of each well were normalized using cell count numbers. Each condition was performed with 4–6 replicates, and the analysis of data was performed using Wave software (Seahorse Bioscience).

### Immunofluorescence staining.

Immunofluorescence staining was carried out by incubation overnight at 4°C with antibodies against COL1A1 (Servicebio, GB11022-1; 1:200), Aspn (Affinity, DF13642; 1:100), Cilp (Huabio, ER1906-32; 1:100), Clu (Proteintech, 66109-1-Ig; 1:100), and Cnmd (Affinity, DF13715; 1:100). Alexa Fluor 594– or Cy3-conjugated secondary antibodies (Invitrogen) were added for 30 minutes, followed by staining with DAPI (Beyotime). Images were acquired with CaseViewer 2.3 scanning software (Servicebio), and analyzed with ImageJ software (NIH; version 1.8.0_112).

### Histology and IHC.

The whole layer of human OA cartilage was fixed in 4% paraformaldehyde and sectioned. Cell pellets and the whole knee joint of mouse were fixed in 4% paraformaldehyde for 48 hours. Knee joints were decalcified with buffered EDTA (0.5 M EDTA, pH 7.4) and dehydrated for paraffin embedding. Sections were deparaffinized, hydrated, and stained with safranin O-fast green according to the manufacturer’s protocols. The OARSI grading system was used to score cartilage destruction in mouse models (47). Sections for IHC were incubated overnight at 4°C with antibodies against MMP-13 (Proteintech, 18165-1-AP; 1:400), NOS2 (Proteintech; 18985-1-AP; 1:100), COL II (Abcam, ab34712; 1:100), human SLC1A5 (Sigma, HPA035239; 1:100), mouse SLC1A5 (Bioss, bs-21518R; 1:100), GLS1 (Proteintech; 12855-1-ap; 1:100), phospho-IκBα (Affinity, AF2002; 1:100), and GS (Abcam, ab176562; 1:200). Staining was visualized using an HRP detection system (Servicebio), and sections were then counterstained with hematoxylin. Images were acquired with CaseViewer 2.3 scanning software (Servicebio) and analyzed by ImageJ software.

### scRNA-seq.

We performed scRNA-seq experiments on articular cartilage isolated from control, DMM, and DM-αKG–treated mice, as previously described (26). The medial tibial plateau was collected in ice-cold DMEM containing 30 μm actinomycin D (HyClone, SH30070.03) 8 weeks after DMM surgery. Mouse joints (*n* = 5 biological replicate samples, each consisting of cells isolated from the joints of 5 animals) were cut into small pieces and digested at 37°C for 2.5 hours in DMEM containing 0.2% NB4 collagenase and 0.05 mg/mL DNase I (Roche). After digestion, the cell suspension was filtered through a 70 μm cell strainer and then centrifuged at 1,000*g* for 5 minutes. After the supernatant was discarded, the cells were resuspended in cold HBSS buffer containing 0.04% BSA for subsequent staining. scRNA-seq datasets were produced using a Chromium system (10x Genomics) according to the manufacturer’s instructions. Library sequencing was performed with a 10x Genomics Single Cell 3′ Solution (Version 2) Kit and subjected to Illumina sequencing (HiSeq 4000). The reads were converted to FASTQ format using mkfastq from CellRanger 2.1.0 (10x Genomics). Alignment, quantitation, and aggregation of the sample count matrices were performed using the CellRanger 2.1.0 pipeline (10x Genomics) and the mouse reference genome. Subsequent analysis was performed using R Bioconductor (version 3.4.2.) and the R package Seurat (version 2.3). Cell clustering analysis was performed via the FindClusters function, and identification of marker analysis between clusters was performed with the FindMarkers function. To visualize the data, nonlinear dimensional reduction was used, and t-SNE plots were created by using the RunTSNE function. Differential gene expression was determined using unique molecular identifier–normalized gene expression data. Analysis of GO term enrichment was performed for differentially expressed genes (log fold change ≥ 0.5 or < –0.5) using the Enrichment Tool package of Bohao Biotechnology.

### Statistics.

All data are presented as the mean ± SEM. All experiments were replicated at least 3 times. Experiments using primary cells were performed with at least 3 biological replicates. For comparisons between 2 independent groups, 2-tailed Student’s *t* test was used. For multiple comparisons, 1-way or 2-way ANOVA was used. For nonparametric data, the Mann-Whitney *U* test was used. Statistical analyses were performed with Prism 7 (GraphPad Software).

### Study approval.

Human articular cartilage samples were obtained from 17 patients with osteoarthritis (*n* = 3 male, 14 female; median age 66 years) who underwent total knee arthroplasty at Shanghai East Hospital. The use of these materials was approved by the Institutional Review Board of Shanghai East Hospital, and all participants provided written informed consent prior to surgery. All animal procedures were approved by the Animal Care and Experiment Committee of the Shanghai Tongji University School of Medicine.

### Data availability.

Uncropped scans of all Western blots and all raw data used to create all graphs can be found in the supplemental material. All individual values represented in graphs are provided in the Supporting Data Values file. The sequencing data are available in the National Center for Biotechnology Information Gene Expression Omnibus under accession SRX19697728 (https://www.ncbi.nlm.nih.gov/sra/SRX19697728). Any additional information required to reanalyze the data reported in this article is available from the lead author upon request.

## Author contributions

SL designed, performed, and analyzed data from most of the experiments and wrote the manuscript. FY, YES, and LC designed experiments and interpreted data. L Lu and TS performed mouse surgery and intra-articular injection. HJ, PJ, JL, SS, QW, RS, XZ, L Li, WL, KF, ZC, SJ, CZ, and FN provided mice, designed experiments, and analyzed and interpreted data. L Lu and HJ valuated the animal samples and data analysis. QW and RS helped collect clinical samples. CZ, ORF, and YZ designed experiments, interpreted data, and revised the manuscript. LC conceived, designed, and supervised the study, analyzed and interpreted data, and critically revised the manuscript. All authors discussed the results and commented on the manuscript.

## Funding support

- Natural Science Foundation of China (grants 82572807 and 81471798).
- Shanghai Science and Technology Innovation Action Plan (grant 23J11900400).
- Shanghai East Hospital characteristic professional subject construction project (grant 2024-DFTS-004).

## Supplementary Material

Supplemental data

Unedited blot and gel images

Supporting data values

## Version 1 03/02/2026

Electronic publication
