# Supplementary material for: α-Ketoglutarate protects against cartilage damage via epigenetically driven metabolic reprogramming in osteoarthritis models
Source: J Clin Invest. 2026 Mar 2;136(5):e172380. doi: 10.1172/JCI172380 (PMC12948417; doi:10.1172/JCI172380)
Supplement: Supplemental data [file jci-136-172380-s065.pdf]

Supplemental information

Materials and methods

Cell transfection

HEK293T cells were grown in DMEM supplemented with 10% FBS and penicillin-streptomycin. Plasmids encoding the HA-TRAF6, Flag-K63-Ubiquitin, MYC-UBE2O were purchased from Wei Nuo Sai Biology, the si-ube2o were purchased from RiboBio. Cells were transfected using Lipofectamine 2000 (Life Technologies) according to the manufacturer's protocol. Briefly, HA-TRAF6, Flag-K63-Ubiquitin, MYC-UBE2O, si-ube2o and si-NC were separately dissolved in DMEM. These mixed solutions were placed at room temperature for 5 mins. Each solution was subsequently mixed with the Lipofectamine 2000 transfection reagent for 20 mins. The transfected cells were incubated in serum-free medium at 37°C, 5% CO_2 for 6h and then replaced the mixed medium with DMEM supplemented with 10% FBS and penicillin-streptomycin.

Metabolomic Analyses

After treatment with IL-1β for 36 h, the primary cultured chondrocytes were harvested, washed twice with PBS and cryopreserved in liquid nitrogen. For analysis of chondrocytes in vivo, mouse articular cartilage on the medial side of the tibial plateau was harvested at 8 weeks after sham or DMM surgery. A pooled sample was created with the tissues from 5 individual mice. Articular cartilage from both mice and humans were weighed (50 mg) and subsequently cryopreserved in liquid nitrogen. The levels of intracellular metabolites were determined by liquid chromatography–tandem mass spectrometry (LC-MS) analysis. In brief, metabolites were extracted using a precooled methanol and H_2O (4:1, v/v) solvent mixture by homogenization with a TissueLyser-192 homogenizer, sonicated in an ice-cold water bath for 10 min, and centrifuged for 10 min (14,000 g, 4°C). The supernatant was dried with a speed vacuum concentrator and reconstituted in acetonitrile and H_2O (1:1, v/v), vortexed for 30 s, sonicated for 3 min and centrifuged for 15 min (13400 g, 4°C), followed by collection of the

supernatant for LC–MS analysis. The supernatants were analyzed by a Shimadzu LC-20AD chromatography system and an AB SCIEX QTRAP 5500 mass spectrometer. Samples were injected onto an iHILIC-Fusion(P) column, and mobile phase A (acetonitrile) and mobile phase B (H_2O/0.2% formic acid) were delivered at a flow rate of 250 nl/min. Quantitative evaluation of amino acids and α-ketoglutarate was performed using the obtained peak area ratios multiplied based on calibration curves with pure standards.

^{13}C labeled glutamine tracer analysis

Chondrocytes at primary passage treated with or without IL-1β, were incubated with glutamine-free DMEM medium containing 10% FBS and 4mM ^{13}C-glutamine (Cambridge, CLM-1822-H). After 6 hours, chondrocytes were harvested and re-suspended in 0.6 ml cold 50% aqueous methanol containing norvaline (100 μM) as an internal standard. After being frozen on dry ice for 30 mins and thawed on ice, 400 μl chloroform was added to the suspension and vortexed for 30s before centrifugation for 10 min at 16,000 g (4 °C). Metabolite analysis using Gas Chromatography-Mass Spectrometer (GC-MS) following the manufacturer's instruction (Agilent Technologies). Briefly, 70 μL of pyridine was added to the dried pellet and incubated for 20 min at 80 °C. After cooling, 30 μL of N-tert-butyldimethylsilyl-N-methyltrifluoroacetamide was added and samples were re-incubated for 1 h at 80 °C before centrifugation for 10 min at 16000 g (4 °C). Metabolites were analyzed using the 7890A GC system combined with the 5975C Inert MS system. Enrichment of [^{13}C] was measured by quantifying the abundance of the following ions: αKG, 304–318 m/z; aspartate, 334–346 m/z; citrate, 465–482 m/z; fumarate, 245–254 m/z; glutamate, 363–377 m/z; and malate, 335–347 m/z. All peaks were manually examined and verified relative to known spectra for each metabolite.

Chromatin immunoprecipitation (ChIP)

ChIP was performed using the ChIP Assay Kit (Millipore, 17-371) according to the manufacturer's instructions. Briefly, 1×10^7 chondrocytes were treated with 1%

formaldehyde for 10 min to cross-link histones to DNA, followed by quenching unreacted formaldehyde with 10x glycine for 5 min. After twice washing with PBS, cells were scraped into conical tube and were lysed in SDS lysis buffer containing protease inhibitor Cocktail II. Chromatin was sheared on ice to generate an average length of 200-500 bp DNA fragments with an ultrasonic processor and centrifuged at 12,000 g for 10 min. The soluble chromatin supernatant was immunoprecipitated with anti-H3K27me3 at 4°C overnight, IgG was used as the negative control. Protein G Agarose-antibody/chromatin complex was washed by resuspending the beads in the low-salt and following high-salt buffer. After incubated with Elution Buffer (contain 20% SDS, 1M $NaHCO_3$ and sterile distilled water) to elute histone complexes, immunoprecipitated DNA and input DNA were analyzed by qRT-PCR, and results are presented as percentage of input. The qPCR primers that used for amplification of promoters are listed in Supplementary Table 1.

RNA sequencing analysis

Total RNA was extracted from mouse articular chondrocytes using TRIzol reagent, and three biological replicates were used for each experimental group. Complementary DNA libraries were prepared with a TruSeq RNA Sample Preparation Kit V2 (Illumina) in accordance with the manufacturer's protocol. Briefly, 2 micrograms of total RNA (RIN > 9.5) from each sample were used for purification with RNA purification beads, and the poly(A) RNA was fragmented with divalent cations under elevated temperature. First-strand cDNA was synthesized from the fragmented RNA by reverse transcription. In second-strand cDNA synthesis, the double-stranded DNA was subsequently adenylated at the 3' ends. Finally, universal adapters were ligated to the cDNA fragments, and PCR amplification was performed to produce the final cDNA library. RNA sequencing was performed using a HiSeq 2500 System. All of the RNA sequencing procedures were performed by Bohao Biotechnology. The reads were processed and aligned to the reference genome (mm10) using the TopHat program. Differential expression of genes was calculated with the DESeq algorithm. Furthermore, GO analysis was performed to elucidate the biological implications of the differentially

expressed genes in the cellular component, biological process, and molecular function categories. Fisher's exact test was used to identify the significantly influenced GO categories and pathways. Pathway analysis was applied to identify the significantly influenced pathways ($P < 0.05$) related to the differentially expressed genes (including upregulated and downregulated genes) according to the Kyoto Encyclopedia of Genes and Genomes (KEGG) database. The mappable results were analyzed using the DESeq2 package.

Ubiquitination assay

The ubiquitination assay was performed with an Immunoprecipitation Kit (Beyotime Biotechnology, P2179S) according to the manufacturer's instructions. Briefly, primary cultured chondrocytes were treated with IL-1β for 0, 5 and 10 min, 2×10^6 cells were harvested and washed twice in cold PBS and lysed in lysis buffer supplemented with protease inhibitor cocktail. Each lysate was centrifuged at 13 000 g at 4 °C for 5 min, and the supernatant was transferred to a new tube. Each supernatant was incubated with Protein A+G magnetic beads bound with TRAF6 antibody for overnight at 4 °C. After three washes with lysis buffer supplemented with protease inhibitor cocktail, bound proteins were recovered by boiling in SDS-PAGE sample loading buffer for 5 min. TRAF6 and K63 ubiquitination of TRAF6 was detected by western blot with antibody against TRAF6 (Santa Cruz, sc-8409, 1:200) and K63 Ub (Cell Signaling Technology, 5621S, 1:1000) respectively.

Supplemental Figure

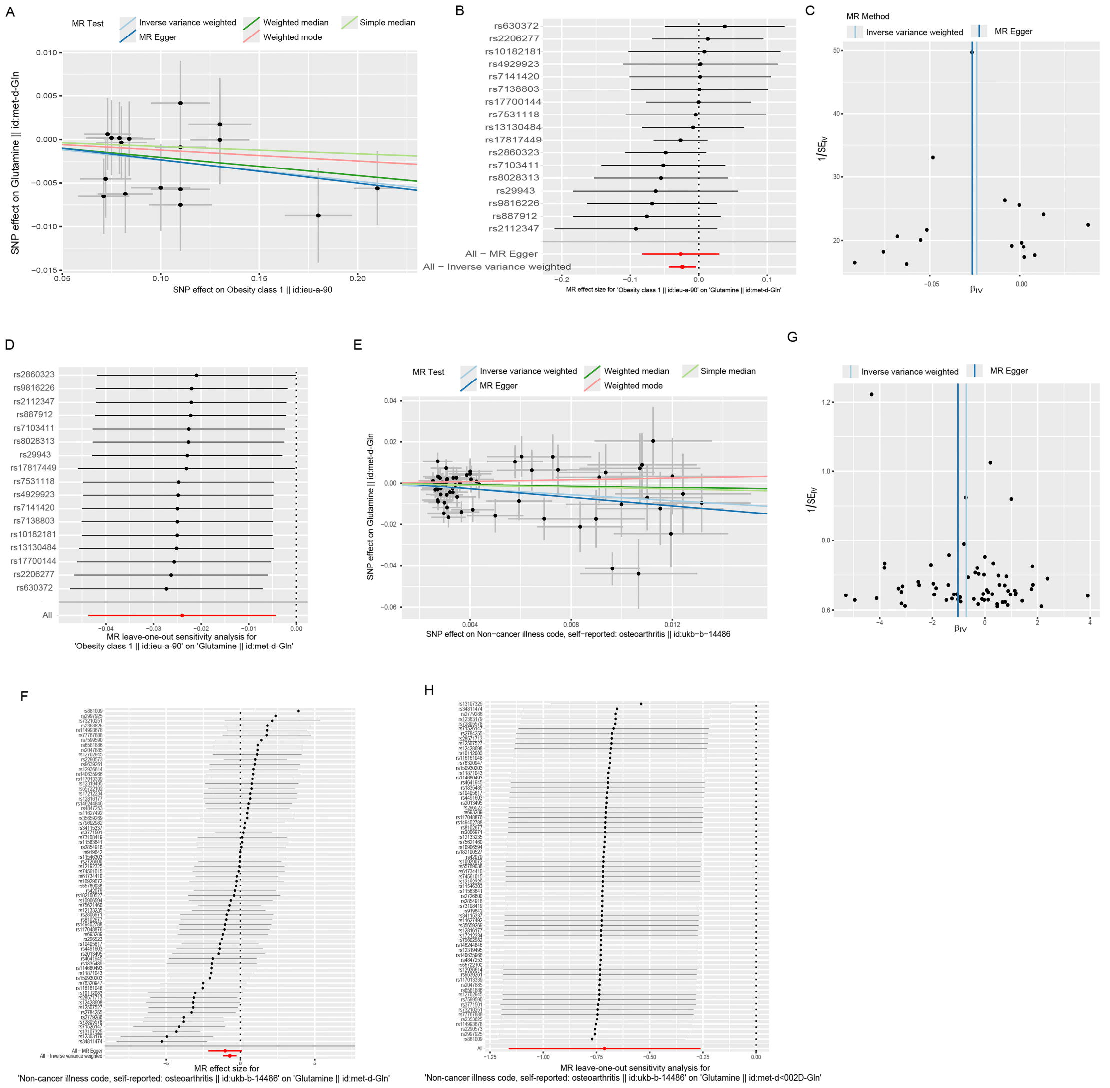

Supplementary Fig 1. Mendelian randomization reveals a causal effect of glutamine metabolism in the pathogenesis of OA.

(A-D) Causal effect of obesity on blood glutamine levels assessed by two-sample Mendelian randomization.

(E-H) Causal effect of OA on blood glutamine levels assessed by two-sample Mendelian randomization.

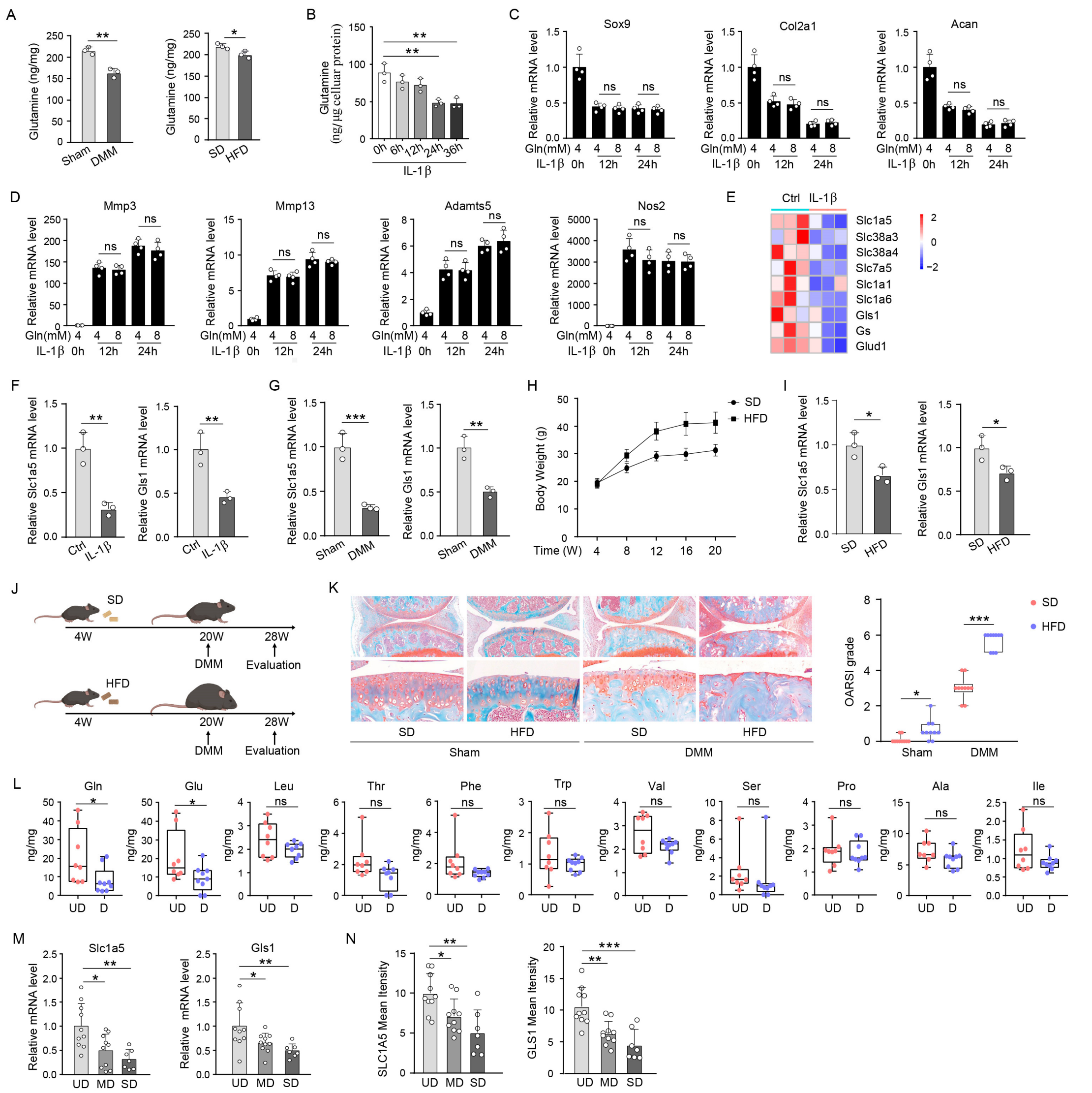

Supplementary Fig 2. Alteration of glutamine metabolism in osteoarthritic chondrocytes.

(A) Glutamine levels in cartilage of SD and HFD-treated mice, as well as in sham- or DMM-operated knee cartilage at 8 weeks post surgery (n=3 per group).

(B) Glutamine levels in chondrocytes treated with or without IL-1β at indicated time points. n=3 per group.

(C, D) qRT-PCR (n = 4 per group) of anabolic genes (C) and catabolic genes (D) in IL-1β-treated chondrocytes supplemented with glutamine (4 or 8 mM) for 12 and 24 hours.

(E) RNA-seq of genes relevant to glutamine metabolism in chondrocytes treated with or without IL-1β for 24 hours. n=3 per group.

(F) qRT-PCR of Slc1a5 and Gls1 in chondrocytes treated with or without IL-1β for 24 hours. n=3 per group.

(G) qRT-PCR (n=3 per group) of Slc1a5 and Gls1 in joint cartilage of sham- or DMM-operated mice at 8 weeks post surgery.

(H) Changes in weight of mice fed a standard diet (SD) or high-fat diet (HFD). n = 15 mice per group.

(I) qRT-PCR (n=3 per group) of Slc1a5 and Gls1 in joint cartilage of SD and HFD mice.

(J) Schematic of dietary exposure in SD and HFD mice.

(K) Safranin-O staining and OARSI score in sham or DMM-operated SD and HFD mice (n = 10 mice).

(L) Quantitative analysis of amino acids in undamaged (n = 8) and damaged (n = 9) human OA cartilage by LC-MS.

(M, N) qRT-PCR and mean intensity of SLC1A5 (M) and GLS1 (N) in undamaged (n=10), mildly damaged (n=10) and severely damaged (n=7) human OA cartilage. Scale bars, 20μm.

The data are presented as boxplots or as the mean ± SEM, and the dots represent biological replicates. One-way ANOVA with Tukey's multiple-comparisons test was used for statistical analysis in B-D, M and N; two-tailed unpaired Student's t tests were performed for A, F, G, I and L. Whitney U test was used for OARSI grade in K. *p < 0.05, **p < 0.01, *** p < 0.001., N.S., not significant.

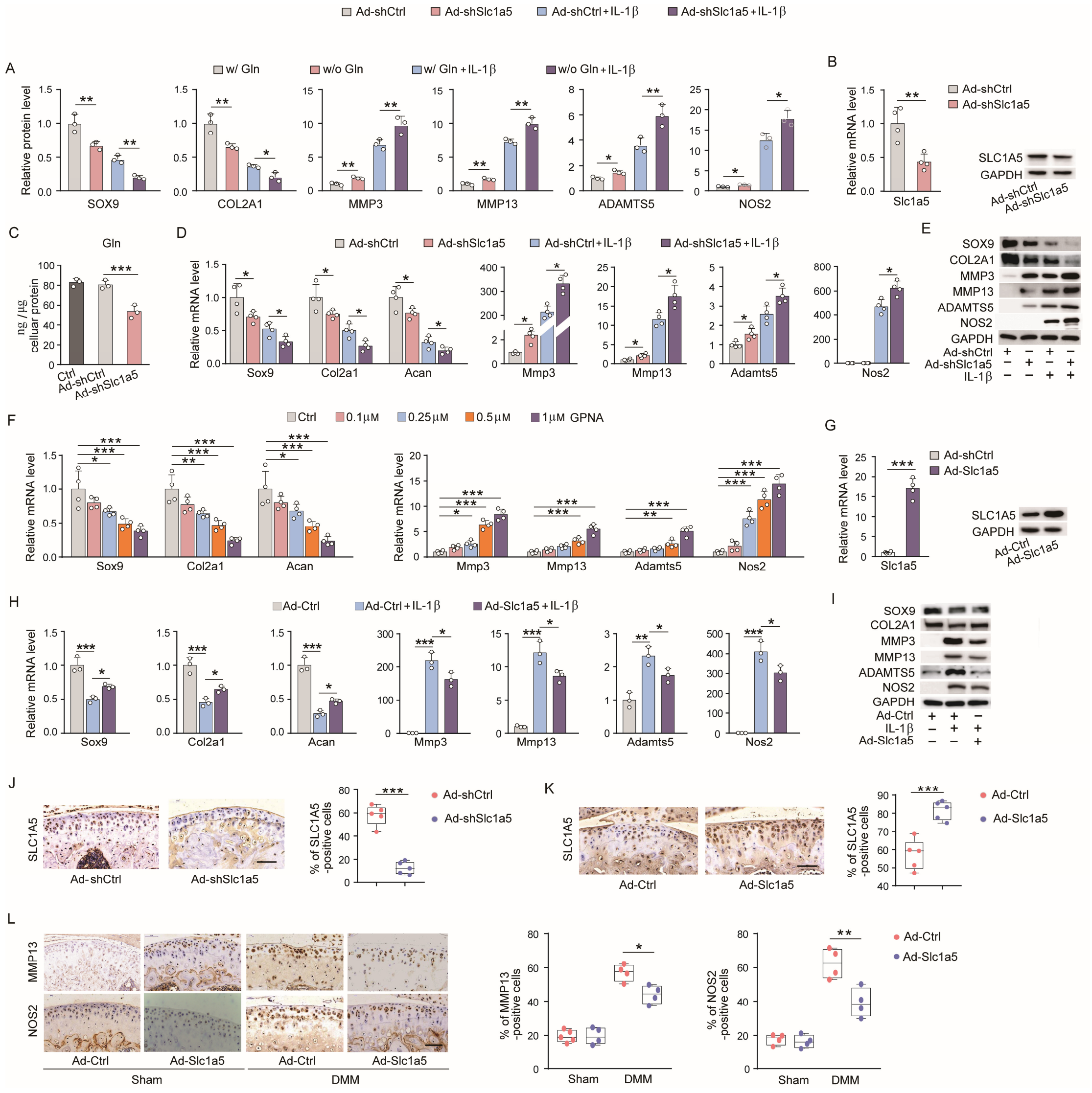

Supplementary Fig 3. SLC1A5 mediates the pathogenesis of OA.

(A) Quantitative analysis of anabolic and catabolic factors in IL-1β-treated chondrocytes with (w) or without (w/o) glutamine conditions for 24 hours. Blots are representative of three independent experiments.

(B) qRT-PCR (n = 4 per group) and western blot analysis of SLC1A5 in chondrocytes infected with Ad-shCtrl or Ad-shSlc1a5 for 72 h. The culture medium contains 4mM glutamine. Blots are representative of three independent experiments.

(C) Glutamine level in chondrocytes infected with Ad-shCtrl or Ad-shSlc1a5 for 72 hours. The culture medium contains 4mM glutamine. n = 3 per group.

(D, E) qRT-PCR (n = 4 per group) (D) and western blot analysis (E) of the indicated anabolic and catabolic factors in the presence or absence of IL-1β (1 ng/ml) for 24 h in chondrocytes after infection with Ad-shCtrl or Ad-shSlc1a5 for 72 h. The culture

medium contains 4mM glutamine. Blots are representative of three independent experiments.

(F) qRT-PCR of anabolic and catabolic factors in chondrocytes treated with GPNA at different doses for 72 hours. The culture medium contains 4mM glutamine. n = 4 per group.

(G) qRT-PCR (n = 4 per group) and western blotting of SLC1A5 in chondrocytes infected with Ad-Ctrl and Ad-Slc1a5 for 72 h. The culture medium contains 4mM glutamine. Blots are representative of three independent experiments.

(H, I) qRT-PCR (n = 3 per group) (H) and western blotting (I) of the indicated anabolic and catabolic factors in chondrocytes infected with Ad-Ctrl or Ad-Slc1a5 under stimulation with IL-1β (1 ng/ml) for 24 h. The culture medium contains 4mM glutamine. Blots are representative of three independent experiments.

(J) Representative images of IHC staining of SLC1A5 in knee joints that underwent intra-articular injection of Ad-shCtrl or Ad-shSlc1a5 weekly three times beginning at 10 weeks. Scale bars, 20 μm. n = 5 mice per group.

(K) Representative images of IHC staining of SLC1A5 in knee joints that underwent intra-articular injection of Ad-Ctrl or Ad-Slc1a5 weekly three times beginning at 10 weeks. Scale bars, 20 μm. n = 5 mice per group.

(L) Representative MMP13 and NOS2 IHC staining images and quantification of cells positive for MMP13 and NOS2 in Ad-shCtrl or Ad-shSlc1a5 mice at 8 weeks after sham and DMM surgery.

The data are presented as boxplots or as the mean ± SEM, and the dots represent biological replicates. Two-tailed unpaired Student's t tests were performed for B, G, J and K; one-way ANOVA with Tukey's multiple-comparisons tests were used for statistical analysis in A, C, D, F, H and L. *$p < 0.05$, **$p < 0.01$, *** $p < 0.001$.

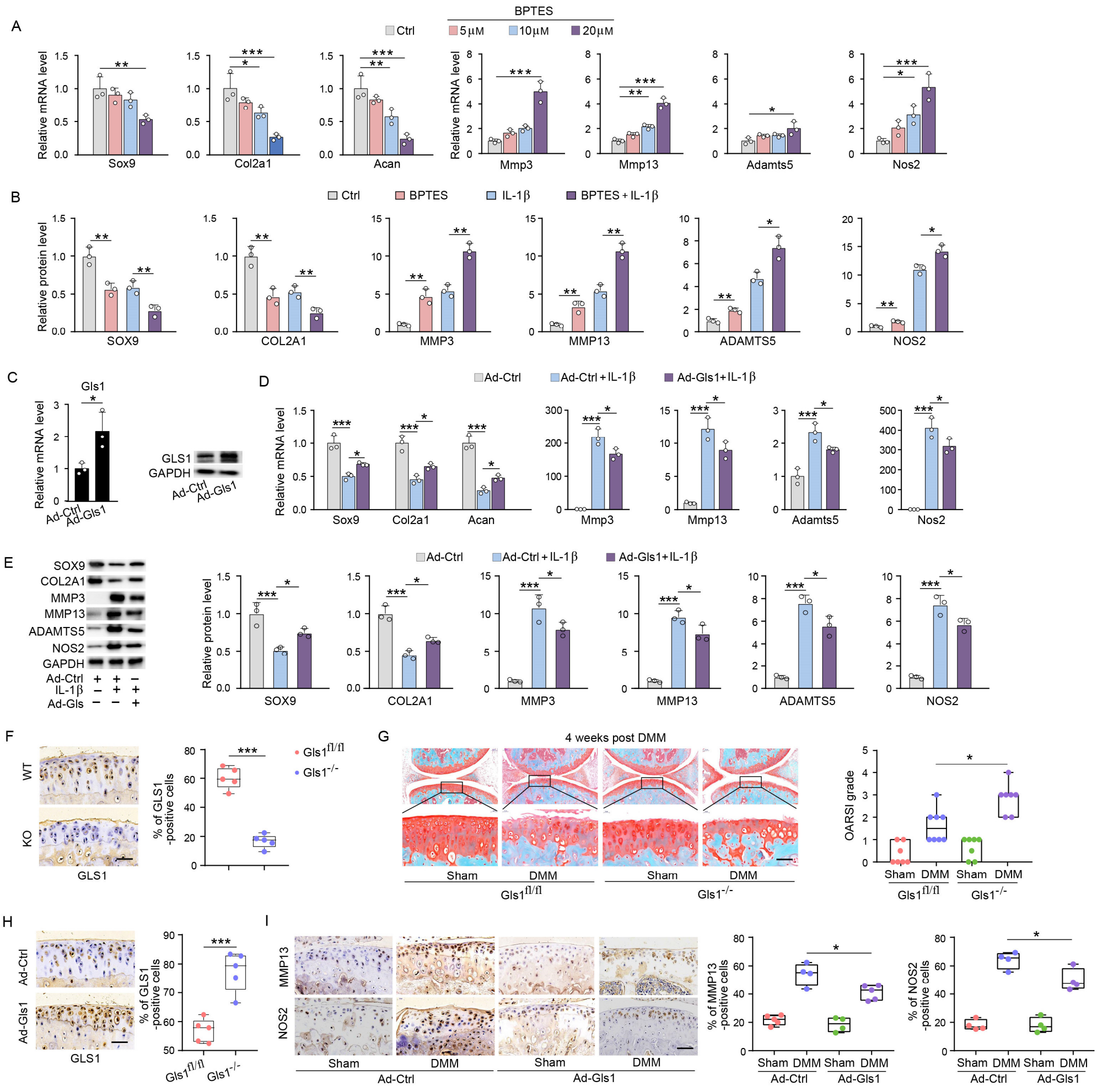

Supplementary Fig 4. GLS1 mediates the pathogenesis of OA.

(A) qRT-PCR of the indicated anabolic and catabolic factors in chondrocytes treated with BPTES at a dose of 0, 5, 10, or 20 μm for 72 hours. n = 3 per group.

(B) Protein quantitative analysis of the indicated anabolic and catabolic factors in chondrocytes treated with BPTES or IL-1β alone or with combined BPTES and IL-1β for 24 hours. The culture medium contains 4mM glutamine.

(C) qRT-PCR (n = 3 per group) and western blotting of GLS1 in chondrocytes infected with Ad-Ctrl or Ad-Gls1 for 72 h. The culture medium contains 4mM glutamine. Blots are representative of three independent experiments.

(D, E) qRT-PCR (n = 3 per group) (D) and western blotting quantitative analysis (E) of the indicated anabolic and catabolic factors in chondrocytes infected with Ad-Ctrl or Ad-Gls1 under stimulation with IL-1β (1 ng/ml) for 24 h. The culture medium contains

4mM glutamine. Blots are representative of three independent experiments.

(F) Representative images of IHC staining of GLS1 in joint sections of Gls1-cKO mice ($Gls1^{-/-}$) and their littermates ($Gls1^{fl/fl}$). Scale bars, 20 µm. n = 5 mice per group.

(G) Representative safranin O/fast green staining images (E) and OARSI scores (sham of $Gls1^{fl/fl}$, n = 7 mice; DMM of $Gls1^{fl/fl}$, n = 8 mice; sham of $Gls1^{-/-}$, n = 7 mice; DMM of $Gls1^{-/-}$, n = 7 mice) (F) for $Gls1^{-/-}$ and control $Gls1^{fl/fl}$ littermates at 4 weeks post sham or DMM surgery.

(H) Representative IHC images of GLS1 in mice that underwent intra-articular injection of Ad-Ctrl or Ad-Gls1 once weekly for 3 weeks. Scale bars, 20 µm.

(I) IHC staining for MMP13 and NOS2 and quantification of cells positive for MMP13 (sham+Ad-Ctrl, n = 5 mice; DMM+Ad-Ctrl, n = 4 mice; sham+Ad-Gls1, n = 4 mice; DMM+ Ad-Gls1, n = 5 mice) and NOS2 (n = 4 mice per group) in joint sections of mice at 8 weeks after DMM surgery. Ad-Ctrl or Ad-Gls1 was intra-articularly injected weekly three times beginning at 10 days after surgery. Scale bars, 20 µm.

The data are presented as the mean ± SEM, and the dots represent biological replicates. A two-tailed unpaired Student's t test was performed for C, F, H; one-way ANOVA with Tukey's multiple-comparisons tests were used for statistical analysis in A, B, D, E, G and I. *$p < 0.05$, **$p < 0.01$, *** $p < 0.001$.

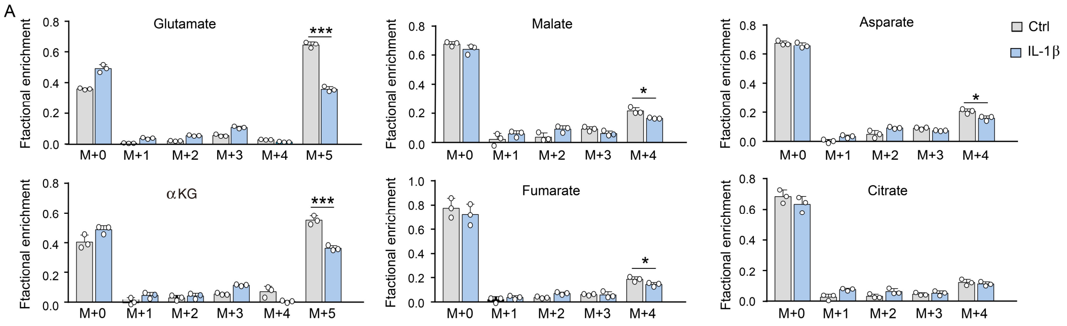

Supplementary Fig 5. The fractional enrichments in the ^{13}C isotopologues of metabolites derived from glutamine.

(A) The fractional enrichments in the ^{13}C isotopologues of metabolites derived from glutamine, including glutamate, α-KG, malate, fumarate, aspartate, citrate, as determined by IC-MS analysis in chondrocytes treated with IL-1β for 6 hours. n = 3 per group.

The data are presented as the mean ± SEM, and the dots represent biological replicates. A two-tailed unpaired Student's t test was performed for A. *p < 0.05, **p < 0.01, *** p < 0.001.

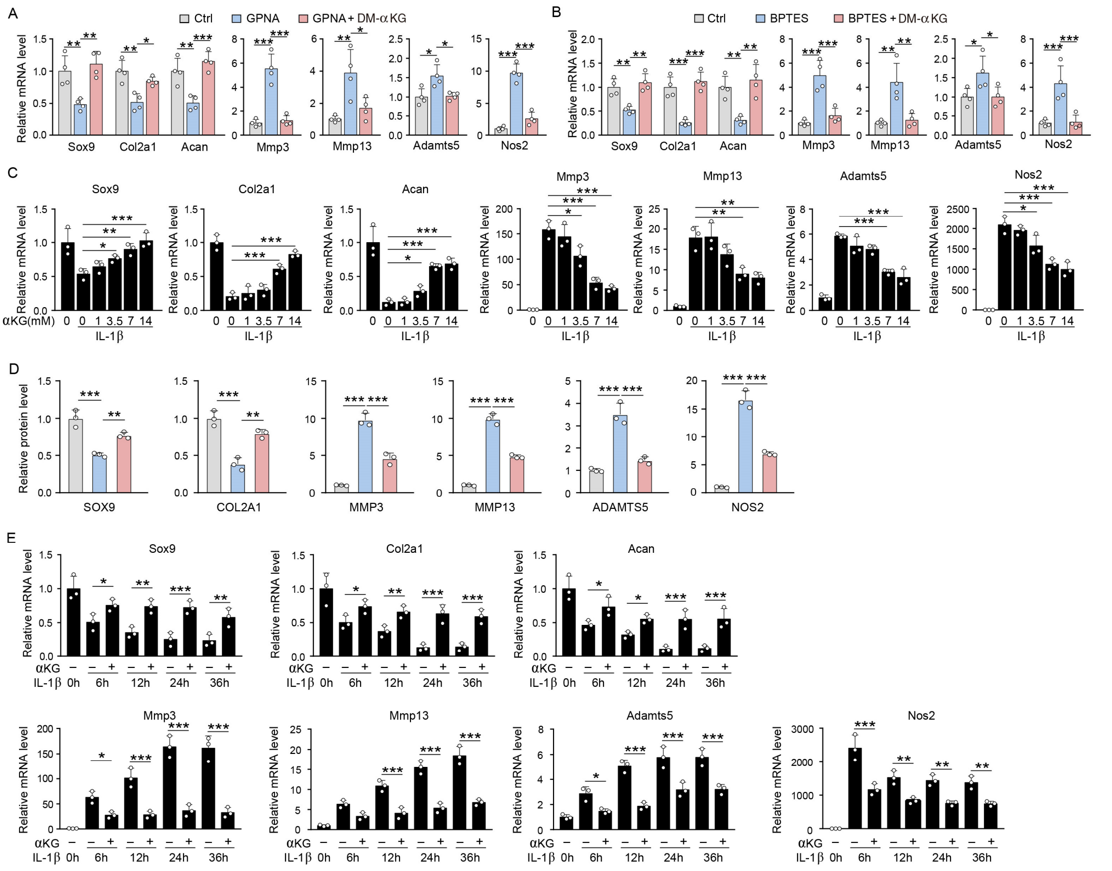

Supplementary Fig 6. αKG attenuates OA progression in vitro.

(A) qRT-PCR of anabolic and catabolic factors in chondrocytes treated with GPNA (0.5 μm) in the presence or absence of DM-αKG (7 mM) for 72 hours. n = 4 per group.

(B) qRT-PCR of the indicated anabolic and catabolic factors in BPTES-treated chondrocytes supplemented with DM-αKG (7 mM) for 72 hours. n = 4 per group.

(C) Restoration of anabolic factors and suppression of catabolic factors by supplementation with different doses of DM-αKG in chondrocytes treated with IL-1β, as assayed by qRT-PCR. n = 3 per group.

(D) Protein quantitative analysis (n=3 per group) of the indicated anabolic and catabolic factors regulated by DM-αKG supplementation in IL-1β-treated chondrocytes.

(E) qRT-PCR (n = 3 per group) of the indicated anabolic and catabolic factors in IL-1β-treated chondrocytes supplemented with DM-αKG (7 mM) at 6, 12, 24, and 36 hours.

The data are presented as the mean ± SEM, and the dots represent biological replicates. One-way ANOVA with Tukey's multiple-comparisons tests were used for statistical analysis in A-E. *p < 0.05, **p < 0.01, *** p < 0.001.

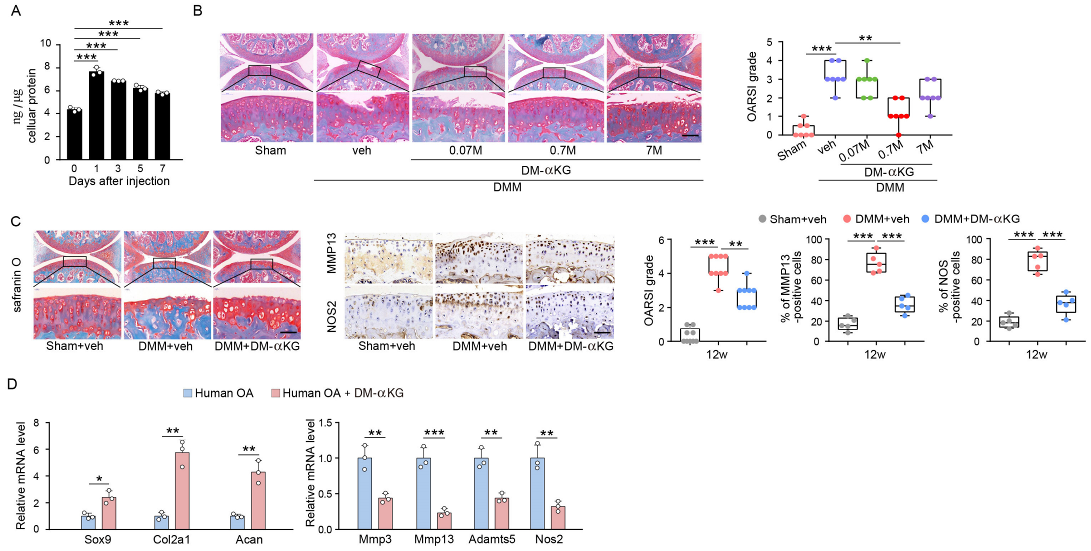

Supplementary Fig 7. αKG attenuates cartilage destruction in OA.

(A) αKG levels in articular cartilage 0, 1, 3, 5, 7 days after intra-articular injection of DM-αKG (0.7 M, per injection). The injections were performed 3 weeks after DMM operation. n = 3 per group.

(B) Safranin O/fast green staining and OARSI scores of knee joints at 8 weeks after sham or DMM surgery. DM-αKG at a dose of 0.07M, 0.7M, 7M (per injection) was intra-articularly injected weekly 5 times beginning at 3 weeks after surgery. Injection of PBS served as a control. n = 7 mice per group. Scale bars, 20 μm.

(C) Representative safranin O/fast green staining images, OARSI scores (n = 9 mice per group), IHC staining for MMP13 and NOS2 and quantification of MMP13- and NOS2-positive cells (n = 5 mice per group) in knee joints injected with DM-αKG or PBS 12 weeks after sham or DMM surgery. Scale bars, 20 μm.

(D) qRT-PCR (n=3 per group) of the indicated anabolic and catabolic factors in pellet cultures of human OA chondrocytes treated with or without DM-αKG for 21 days. Scale bars, 200 μm.

The data are presented as boxplots or as the mean ± SEM, and the dots represent biological replicates. One-way ANOVA with Tukey's multiple-comparisons tests were used for statistical analysis in A and C (percentage of positive cells). A two-tailed unpaired Student's t test was performed for D. Whitney U tests were used for OARSI grade in B and C. *p < 0.05, **p < 0.01, *** p < 0.001. Scale bars, 100 μm.

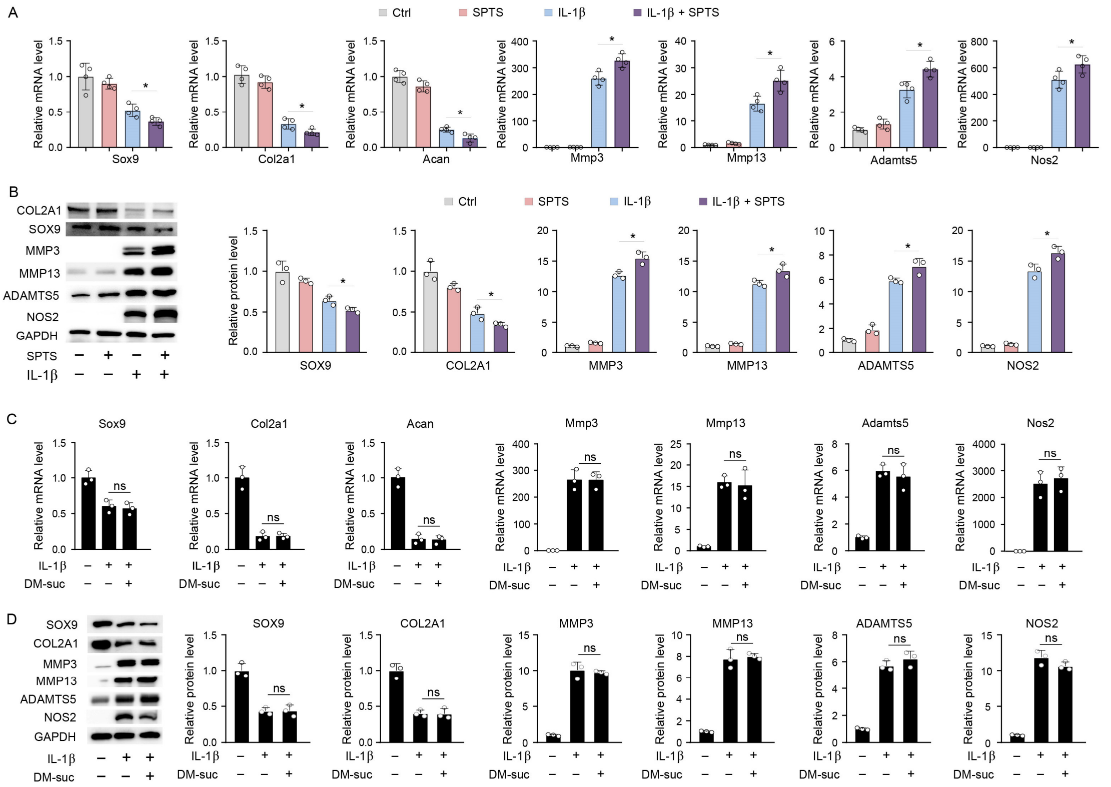

Supplementary Fig 8. Disruption in the TCA cycle increased imbalance of anabolic and catabolic factors and Succinate did not rescue the imbalance of anabolic and catabolic factors in IL-1β-treated chondrocytes.

(A, B) qRT-PCR (n = 4 per group) (A) and western blot quantitative analysis (n = 3 per group) (B) of the indicated anabolic and catabolic factors regulated by SPTP supplementation in IL-1β-treated chondrocytes for 24 hours. Blots are representative of three independent experiments.

(C, D) qRT-PCR (n = 3 per group) (C) and western blot quantitative analysis (n = 3 per group) (D) of the indicated anabolic and catabolic factors in IL-1β-treated chondrocytes supplemented with diethyl-succinate (5 mM) for 24 hours. Blots are representative of three independent experiments.

The data are presented as the mean ± SEM, and the dots represent biological replicates. One-way ANOVA with Tukey's multiple-comparisons tests were used for statistical analysis in A-D. *$p < 0.05$, **$p < 0.01$, *** $p < 0.001$.

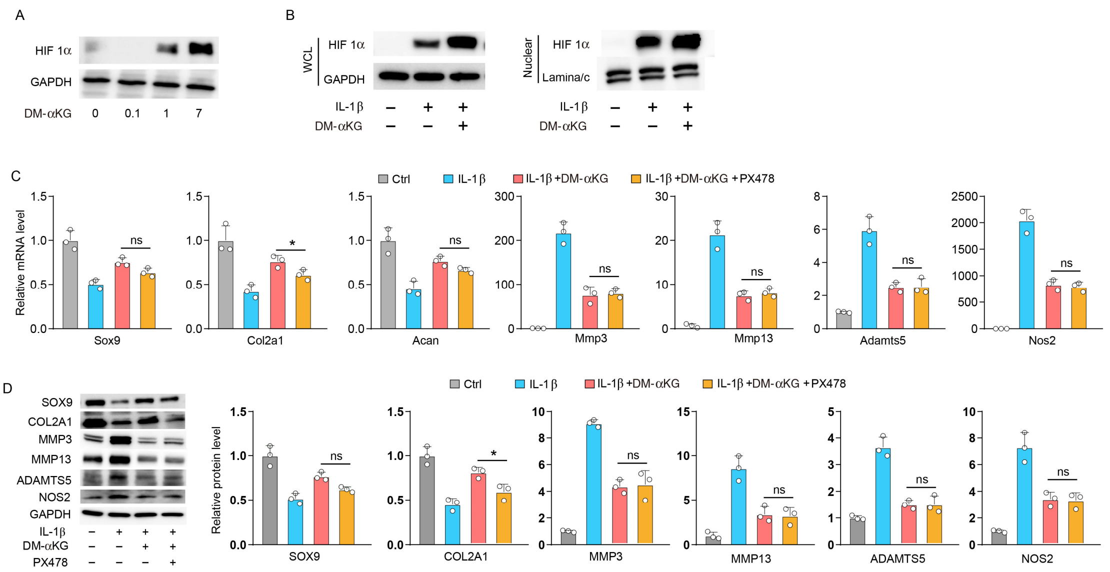

Supplementary Fig 9. αKG-mediated chondroprotection operates independently of HIF-1α signaling

(A) Western blot analysis showing HIF-1α protein levels in chondrocytes treated with varying concentrations of DM-αKG for 24 hours.

(B) Western blot detection of HIF-1α expression in IL-1β-stimulated chondrocytes following DM-αKG supplementation for 24 hours. WCL, whole-cell lysate. Blots are representative of three independent experiments.

(C, D) Quantitative analysis of qRT-PCR (n=3 per group) (C) and western blot (D) analysis for the indicated anabolic and catabolic factors regulated by αKG and PX478 supplementation in IL-1β-treated chondrocytes. Blots are representative of three independent experiments.

The data are presented as the mean ± SEM, and the dots represent biological replicates. One-way ANOVA with Tukey's multiple-comparisons tests were used for statistical analysis in C and D. *$p < 0.05$, **$p < 0.01$, *** $p < 0.001$.

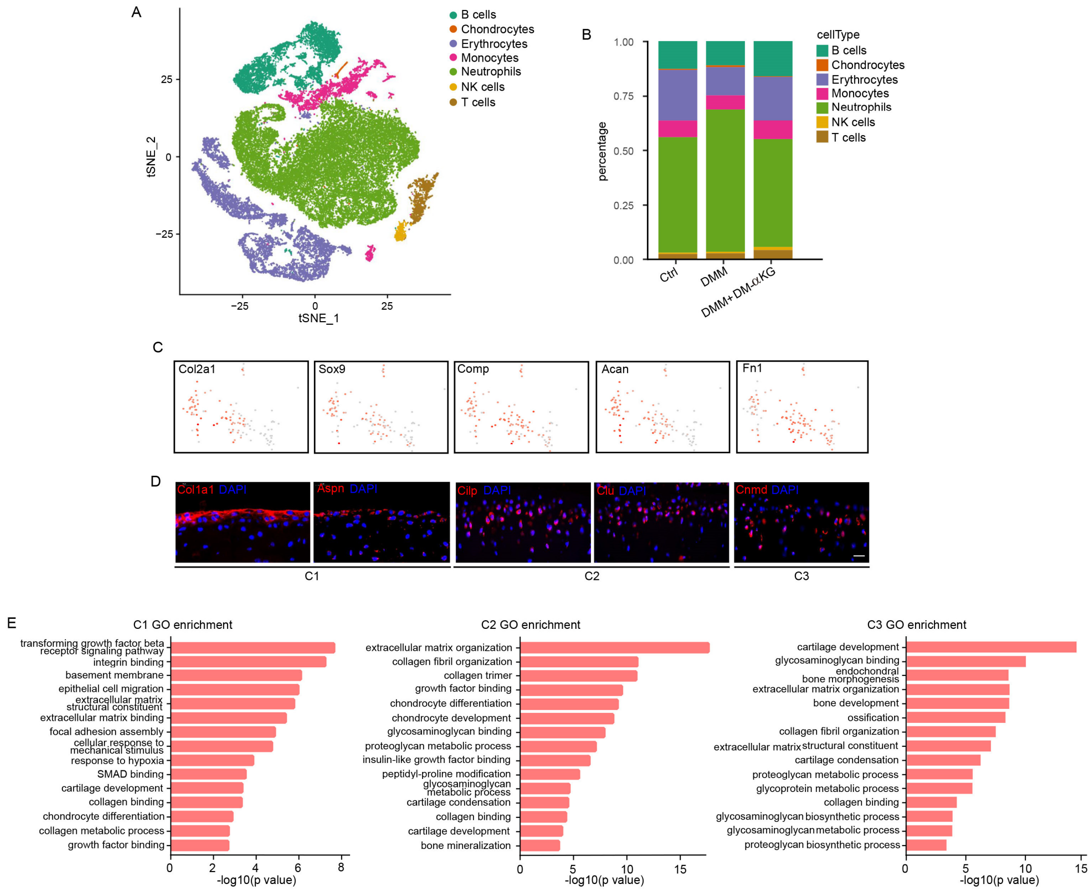

Supplementary Fig 10. Single-cell RNA sequencing analysis of mouse chondrocytes.

(A) Cell clusters of cell identity in cartilage and bone marrow visualized by tSNE. Colors indicate cell types. Each dot represents one cell.

(B) Cell percentages in each cluster of control, DMM and DMM + DM-αKG mice.

(C) Chondrocyte populations and feature plots of chondrocyte marker genes in each of the automatically assigned clusters.

(D) Representative immunofluorescence staining of Aspn, Col1a1, Clip, Clu, and Cnmd in cartilage tissues. Scale bar, 20 μm.

(E) GO category analysis for clusters C1, C2 and C3.

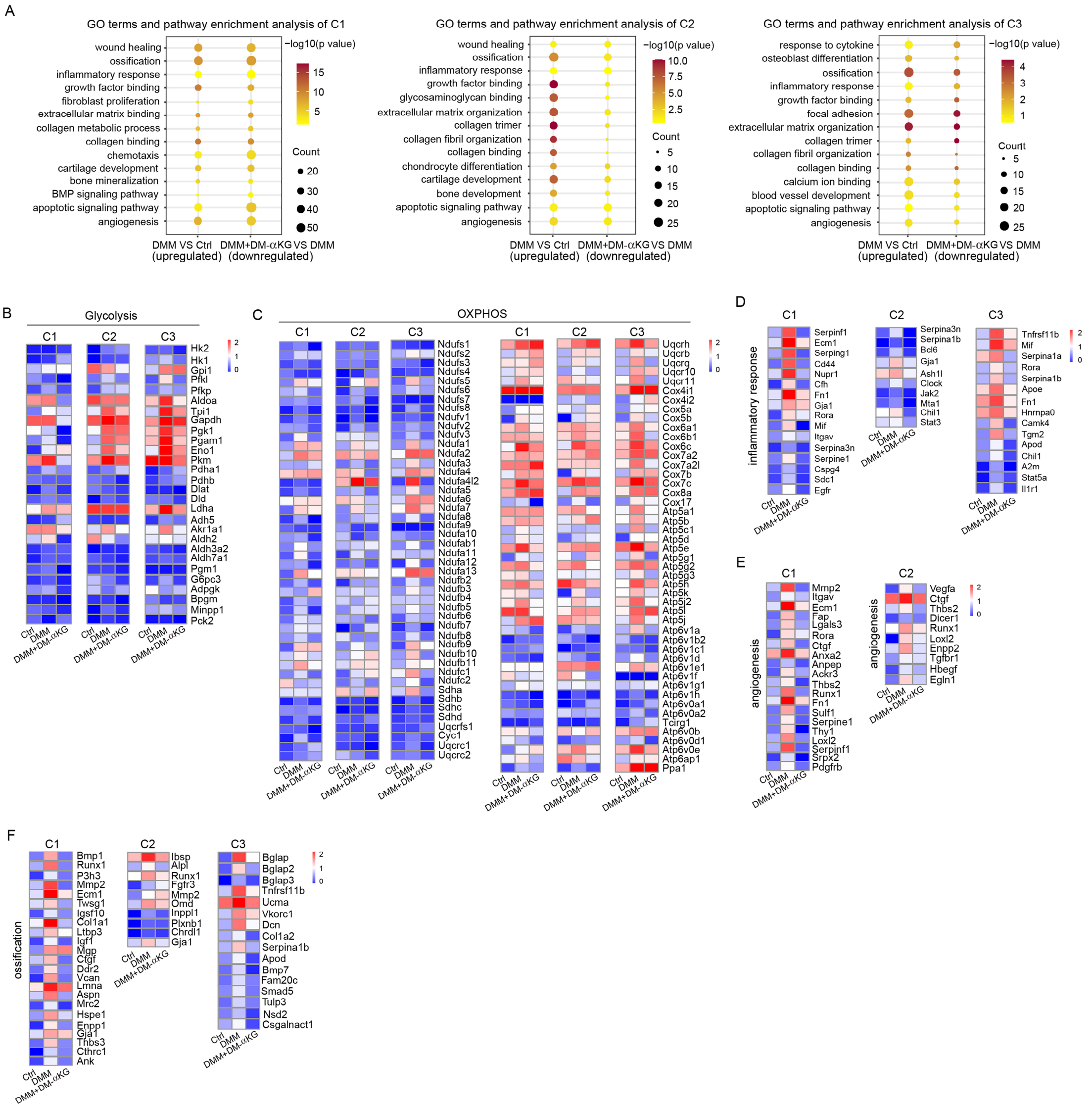

Supplementary Fig 11. ScRNA-seq shows that genes of metabolism in three clusters of articular chondrocytes.

(A) GO terms and pathway enrichment analysis results for upregulated genes in the DMM group versus the ctrl group and downregulated genes in the DMM+DM-αKG group versus the DMM group in C1, C2 and C3

(B-C) Heatmaps of genes related to glycolysis (B) and OXPHOS (C) in the control, DMM and DMM+DM-αKG groups.

(D-E) Heatmaps of genes related to inflammatory response (D) and angiogenesis (E) in the control, DMM and DMM+DM-αKG groups.

(F) Heatmaps of genes related to ossification in the control, DMM and DMM+DM-αKG groups.

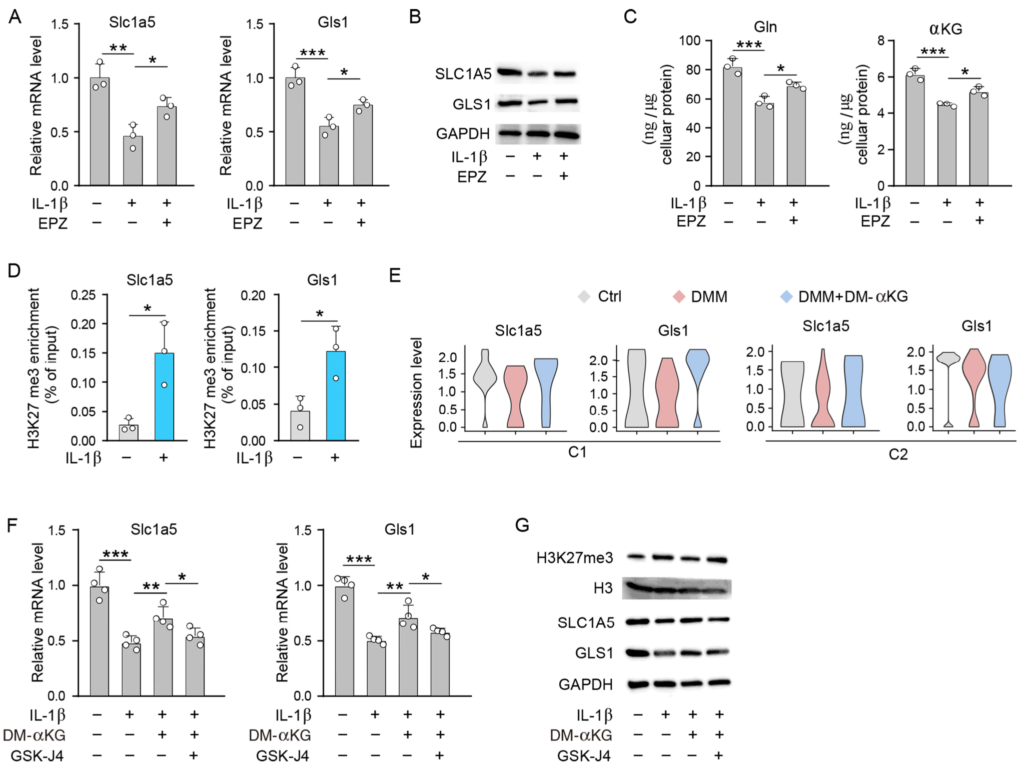

Supplementary Fig 12. Epigenetic inhibition of glutaminolysis genes via H3K27me3 methylation is regulated by αKG availability.

(A, B) qRT-PCR (n=3 per group) (A) and western blot (B) of SLC1A5 and GLS1 in chondrocytes treated with IL-1β or with combined H3K27-specific methylation inhibitor EPZ005687 and IL-1β for 24 hours. Blots are representative of three independent experiments.

(C) Glutamine and αKG levels in chondrocytes treated with IL-1β or with combined H3K27-specific methylation inhibitor EPZ005687 and IL-1β for 24 hours. n=3 per group.

(D) Chondrocytes were treated with IL-1β for 24h, and then cells were harvested for ChIP assay using an antibody against H3K27me3. Immunoprecipitation of the Slc1a5 and Gls1 were analysed by qRT-PCR. n=3 per group.

(E) Violin plots displaying the genes associated with glutamine metabolism in the control, DMM and DMM+DM-αKG groups.

(F, G) qRT-PCR (n=4 per group) (F) and western blot (G) of H3K27me3, SLC1A5 and GLS1 in IL-1β-treated chondrocytes supplemented with DM-αKG and GSK-J4 for 24 hours. Blots are representative of three independent experiments.

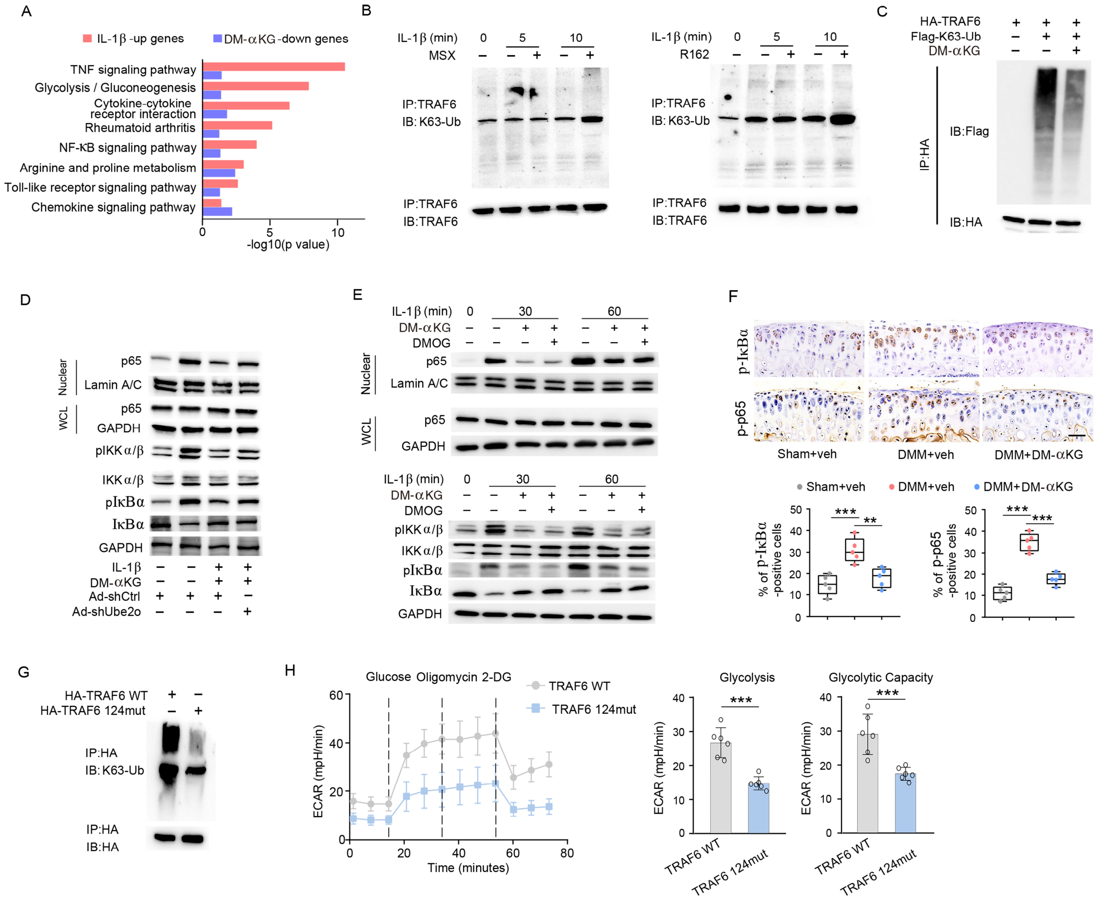

Supplementary Fig 13. Supplementation of DM-αKG suppressed the NF-κB pathway in OA chondrocytes.

(A) KEGG analysis of up- or downregulated genes regulated by DM-αKG supplementation in IL-1β-treated chondrocytes.

(B) Western blot analysis of K63-linked ubiquitination of endogenous TRAF6 in IL-1β stimulated chondrocytes in the presence of MSX, R162, respectively. Blots are representative of three independent experiments.

(C) Immunoblot analysis of K63-linked ubiquitination of TRAF6 in HEK293T cells transfected to express HA-TRAF6 with or without Flag-tagged K63-linked ubiquitin (Flag-K63-Ub) and DM-αKG.

(D) Western blot analysis of p65, IKKα/β, p-IKKα/β, IκBα and p-IκBα from Ad-shCtrl and Ad-shUbe2o chondrocytes stimulated with IL-1β and DM-αKG for 60 min. WCL, whole-cell lysate. Blots are representative of three independent experiments.

(E) Western blot analysis of NF-κB p65, IKKα/β, p-IKKα/β, IκBα and p-IκBα in IL-

1β-treated chondrocytes supplemented with DM-αKG alone or a combination of DM-αKG and DMOG. WCL, whole-cell lysate. Blots are representative of three independent experiments.

(F) IHC staining for pIκBα, p-p65 and quantification of pIκBα- and p-p65-positive cells after sham or DMM surgery and treatment with veh or DM-αKG. n=5 mice per group.

(G) The effect of the mutation at the 124th ubiquitination site mutation on TRAF6 ubiquitination, TRAF6 or TRAF6 124mut construct were transfected into C28/I2 cells, respectively. Ubiquitinated TRAF6 was detected by western blot. Blots are representative of three independent experiments.

(H) ECAR of C28/I2 after transfection with pCDH-3×Flag-TRAF6 or pCDH-3×Flag-TRAF6 124mut plasmids, as measured by the Seahorse Analyzer. Quantification of glycolysis from one timepoint in Glycolysis Stress Test. n=6 per group.

The data are presented as boxplots or as the mean ± SEM, and the dots represent biological replicates. A two-tailed unpaired Student's t test was performed for G; One-way ANOVA with Tukey's multiple-comparisons test was used for statistical analysis in E. *$p < 0.05$, **$p < 0.01$, *** $p < 0.001$.

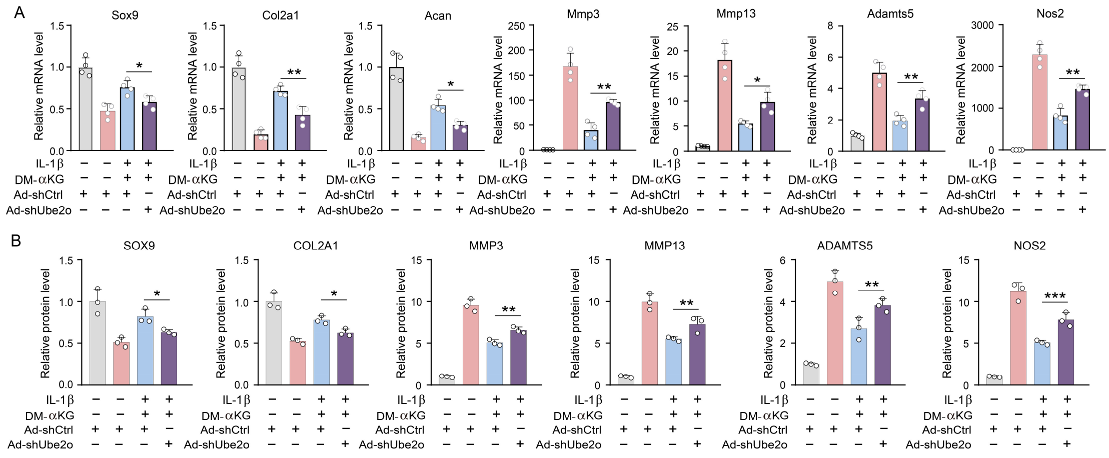

Supplementary Fig 14. DM-αKG regulates anabolic and catabolic genes through UBE2O.

(A) qRT-PCR (n=4 per group) of the indicated anabolic and catabolic factors from Ad-shCtrl and Ad-shUbe2o chondrocytes stimulated with IL-1β and DM-αKG.

(B) Protein quantitative analysis (n=3 per group) of the indicated anabolic and catabolic factors from Ad-shCtrl and Ad-shUbe2o chondrocytes stimulated with IL-1β and DM-αKG.

The data are presented as the mean ± SEM, and the dots represent biological replicates. One-way ANOVA with Tukey's multiple-comparisons tests were used for statistical analysis in A and B. *$p < 0.05$, **$p < 0.01$, *** $p < 0.001$.

Supplementary Table 1. Sequence of primers

Gene	Primer (F=forward; R=reverse)	Amplicon size (bp)
β-Actin (mouse)	F: 5'GCCACTGTCGAGTCGCGT3'	275
	R: 5'GATACCTCTCTTGCTCTGGGC3'	
Sox 9 (mouse)	F: 5'AGTACCCGCATCTGCACAAC3'	187
	R: 5'ACGAAGGGTCTCTTCTCGCT3'	
	F: 5'AGCAAGGTGACCAGGGTATT3'	98

COL2A1(mouse)	R: 5’ACCAGGAGAGCCACGTTC3’	
ACAN（mouse）	F: 5’CGTTGCAGACCAGGAGCAAT3’	275
	R: 5’GGTTTGGACGCCACTTCTCA3’	
MMP3（mouse）	F: 5’TCCTGATGTTGGTGGCTTCAG3’	102
	R: 5’TGTCTTGGCAAATCCGGTGTA3’	
MMP13（mouse）	F: 5’ACTACCATCCTGCGACTCTTG3’	111
	R: 5’GTTTGCCAGTCACCTCTAAGC3’	
ADAMTS（mouse）	F:5’GCCATTGTAATAACCCTGCACC3’	292
	R:5’TCAGTCCCATCCGTAACCTTTG3’	
NOS2（mouse）	F: 5’ACCTTGTTCAGCTACGCCTT3’	112
	R: 5’CATTCCCAAATGTGCTTGTC3	
GLS1（mouse）	F: 5’AACGTCAGATGGTGTCATGCT3’	206
	R: 5’TGAATTTGGCCAGCTGAGGA3’	
SLC1A5（mouse）	F: 5’TCGTCTTTGGTGTGGCTCTG3’	176
	R: 5’CTGGCGGACGTCTTTCATCT3’	
GS（mouse）	F: 5’CTGGGTTGATGGTACCGGAG3’	177
	R:5’CGGAAGGGGTCTCGAAACAT3’	
GLUD1（mouse）	F: 5’CAATGCGCATGCCTGTGTTA3’	205
	R:5’CATAGAGTGCAGGCCCACAT3’	
Il1b（mouse）	F: 5’TGCCACCTTTTGACAGTGATG3’	138
	R:5’TGATGTGCTGCTGCGAGATT3’	
Tnf（mouse）	F: 5’CTGAACTTCGGGGTGATCGG3’	170
	R:5’TTGAGATCCATGCCGTTGGC3’	
UBE2O (mouse)	F: 5’AAGCTGAAACTGGAGGACCG3’	277
	R:5’AGCACCTGGCACCATTAGAC3’	
Sox 9（human）	F: 5’GGCAGCTGTGAACTGGCCA 3’	290
	R:5’GCACACGGGGAACTTGTCC 3’	

COL2A1（human）	F: 5’CCGTGCTCCTGCCGTTTC3’	294
	R:5’CTGAGGCAGTCTTTCACGTCT3’	
ACAN（human）	F:5’TGGGAACCAGCCTATACCCCAG3’	112
	R:5’CAGTTGCAGAAGGGCCTTCTGTAC3’	
MMP3（human）	F: 5’CACTCACAGACCTGACTCGG3’	82
	R: 5’GAGTCAGGGGGAGGTCCATA3’	
MMP13（human）	F: 5’TGGTCCAGGAGATGAAGACC3’	97
	R:5’TCCTCGGAGACTGGTAATGG3’	
ADAMTS（human）	F: 5’CAAGAGCCTGGAAGTGAGCA3’	205
	R:5’GCTGCGCTCTGGAGAACATA3’	
NOS2（human）	F: 5’TCCAAATCTTGCCTGGGGTC3’	218
	R:5’TCTCCCGTCAGTTGGTAGGT3’	
GLS1（human）	F:5’GCAGAGGGTCATGTTGAAGTTG3’	102
	R: 5’CAGTGCTTCATCCATGGGAGT3’	
SLC1A5（human）	F: 5’GTGTCCTCACTCTGGCCATC3’	130
	R: 5’CCCAGAGCGTCACCTTCTAC3’	
GS（human）	F: 5’AAGGCGCTTGGTTGGC3’	300
	R: 5’CTCAGGCAACTCTTCCACACA3’	
β-Actin（human）	F: 5’GAAGGATTCCTATGTGGGCG3’	276
	R: 5’GATAGCACAGCCTGGATAGCA3’	
