# Supplementary material for: α-Ketoglutarate protects against cartilage damage via epigenetically driven metabolic reprogramming in osteoarthritis models
Source: J Clin Invest. 2026 Mar 2;136(5):e172380. doi: 10.1172/JCI172380 (PMC12948417; doi:10.1172/JCI172380)
Supplement: Unedited blot and gel images [file jci-136-172380-s066.pdf]

Figure 1E

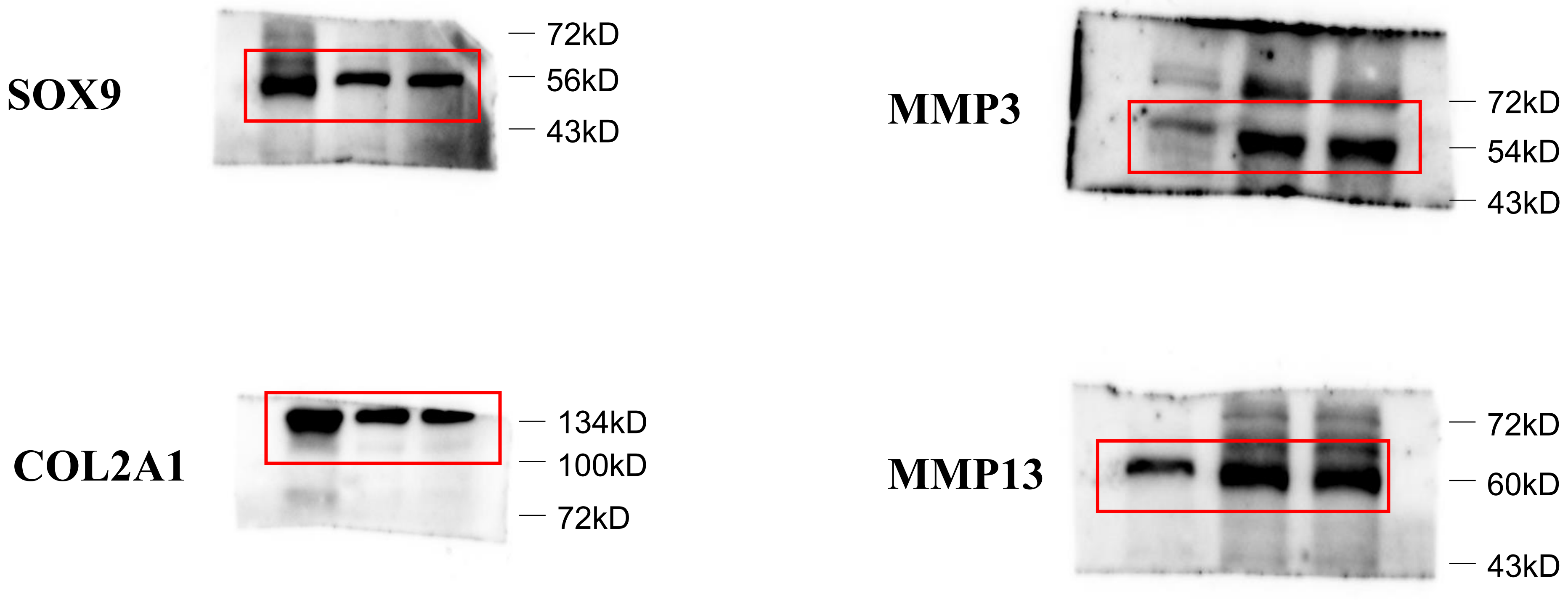

SOX9
72kD
56kD
43kD
MMP3
72kD
54kD
43kD
COL2A1
134kD
100kD
72kD
MMP13
72kD
60kD
43kD

Figure 1E

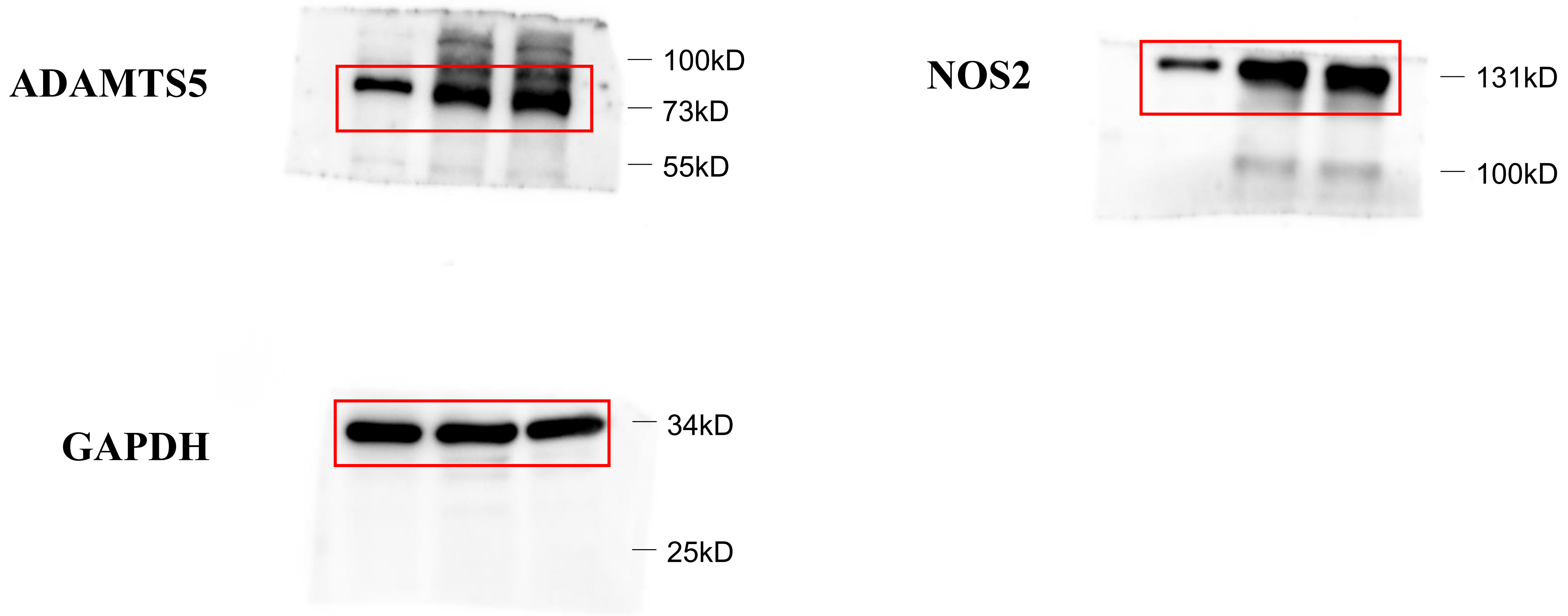

Figure 1G

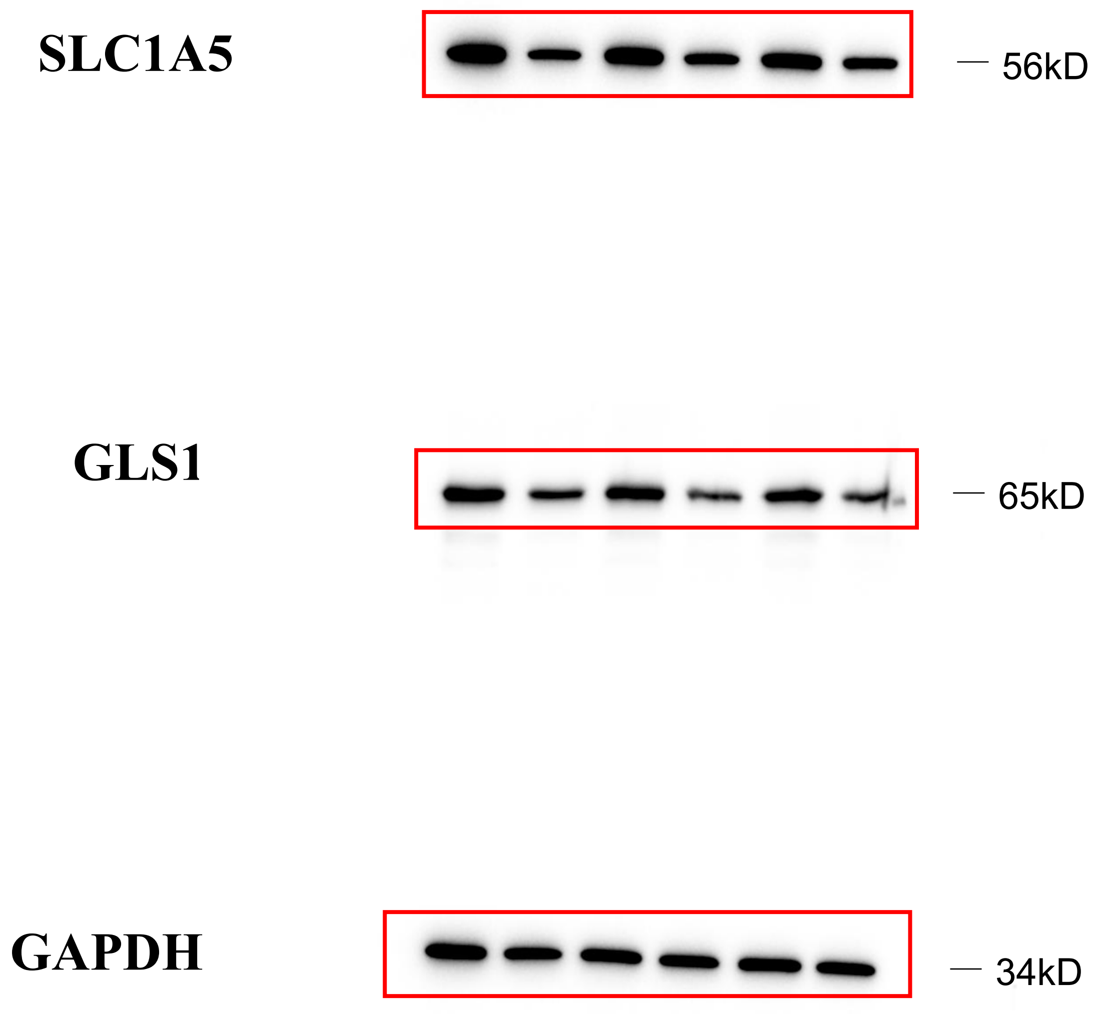

Figure 2B

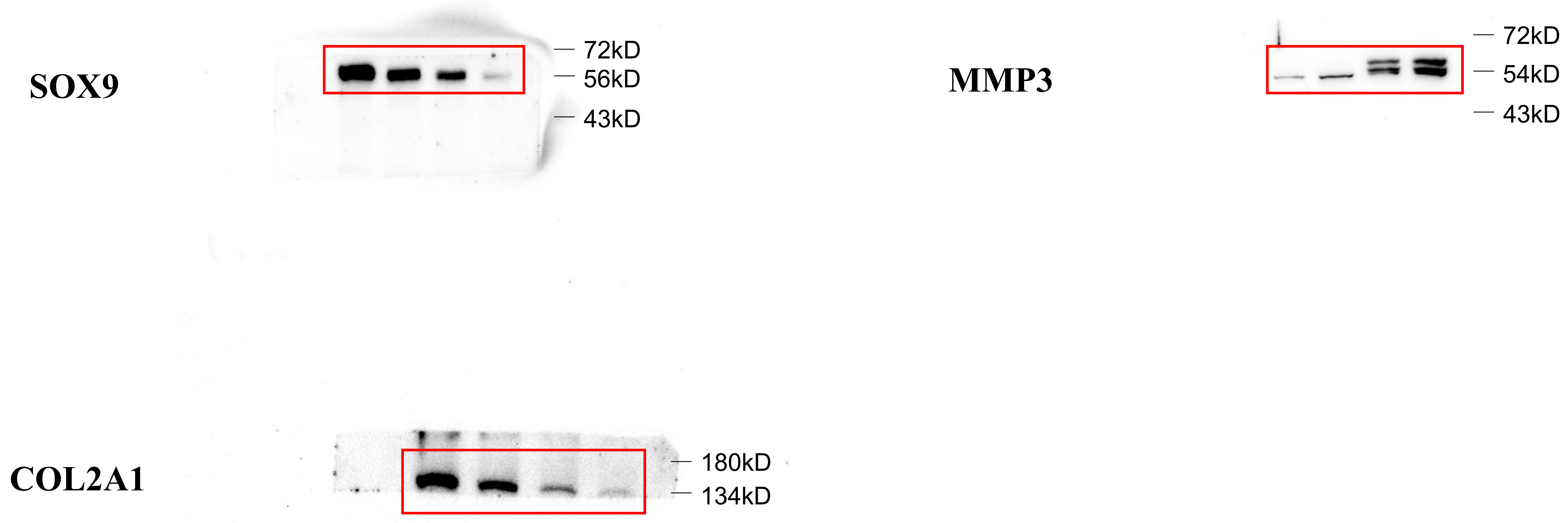

Figure 2B

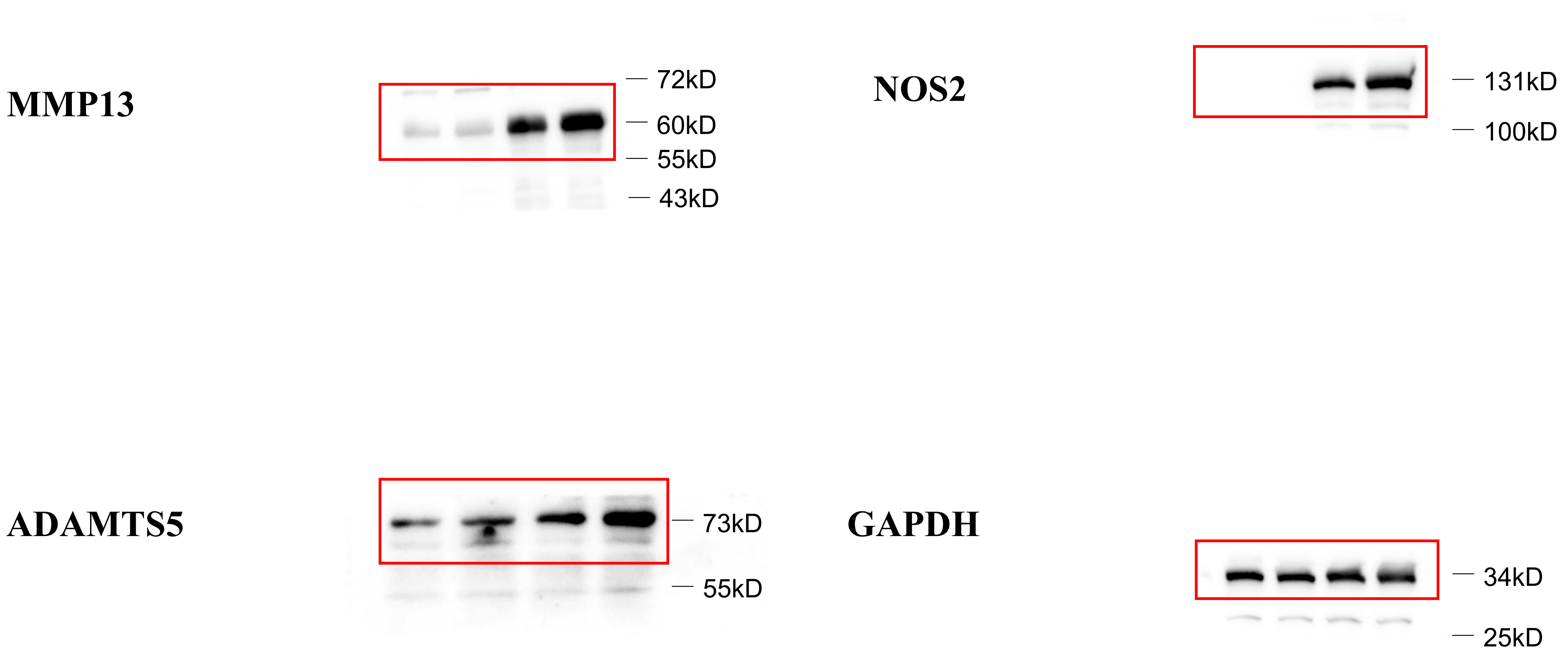
MMP13
72kD
60kD
55kD
43kD
NOS2
131kD
100kD
ADAMTS5
73kD
55kD
GAPDH
34kD
25kD

Figure 3B

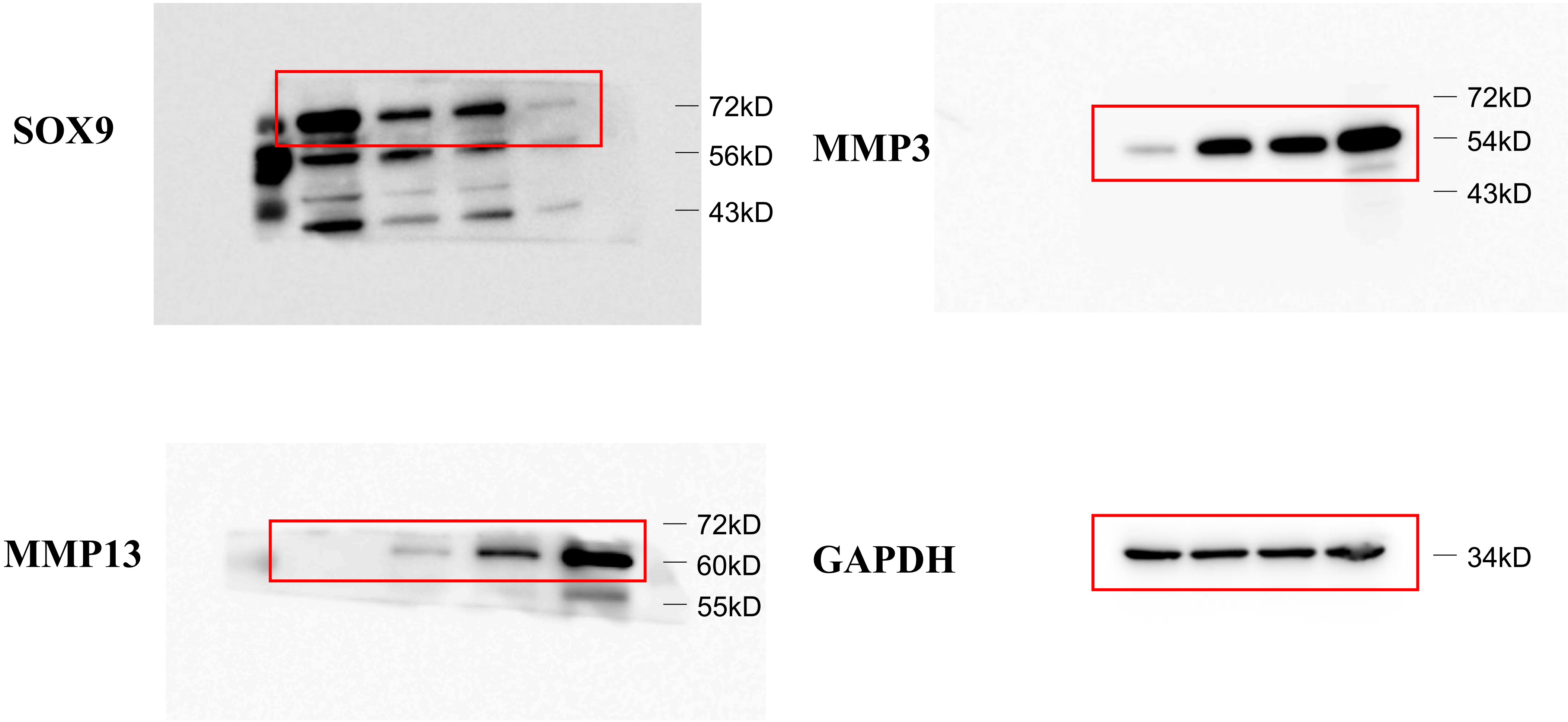
SOX9
72kD
56kD
43kD
MMP3
72kD
54kD
43kD
MMP13
72kD
60kD
55kD
GAPDH
34kD

Figure 3B

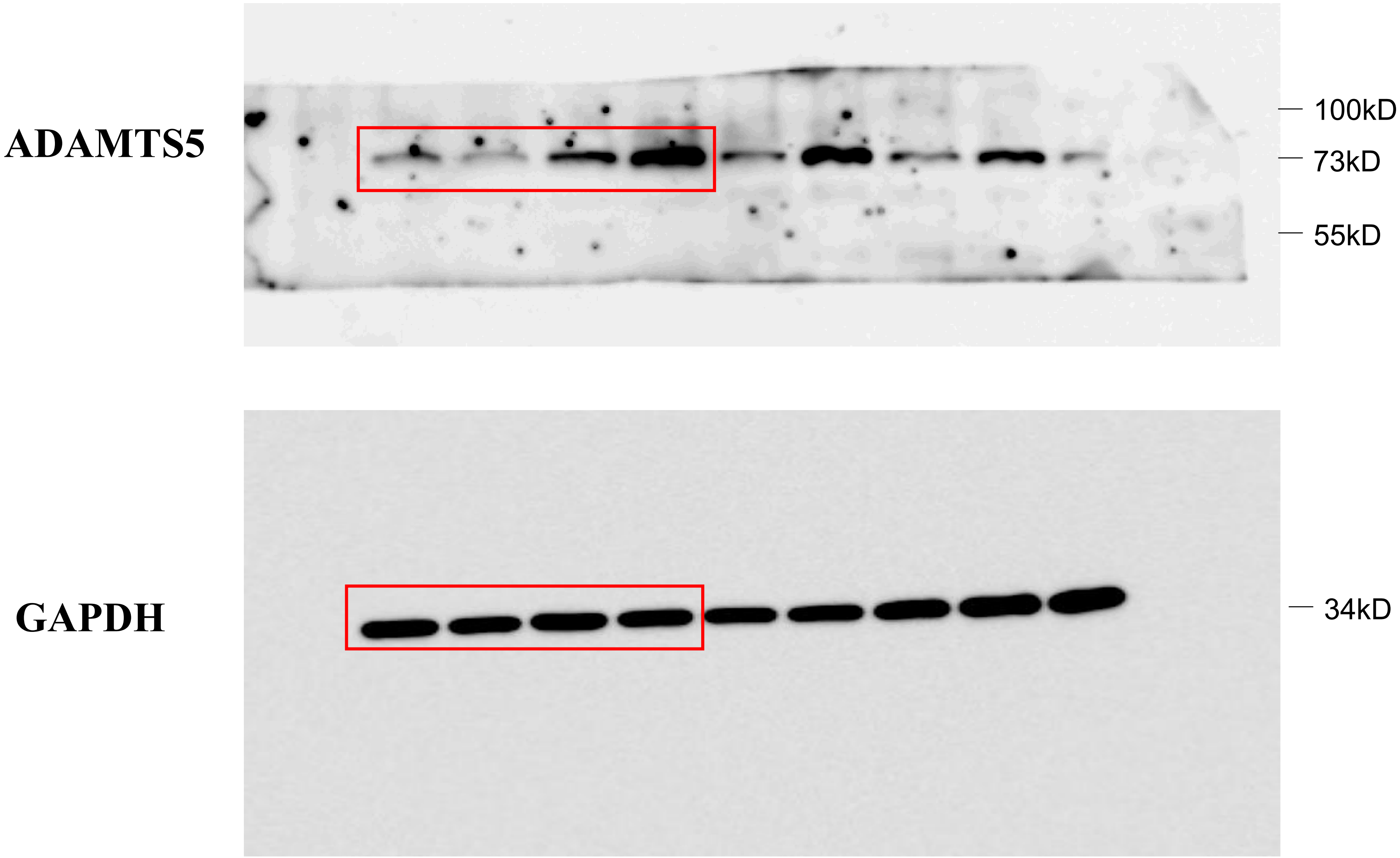

Figure 3B

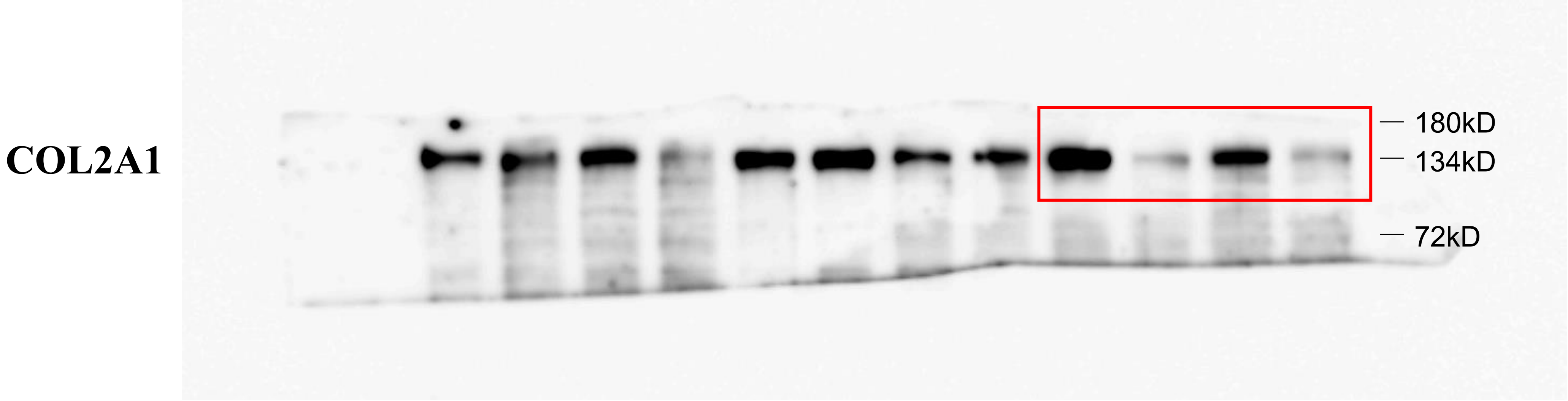

Figure 3B

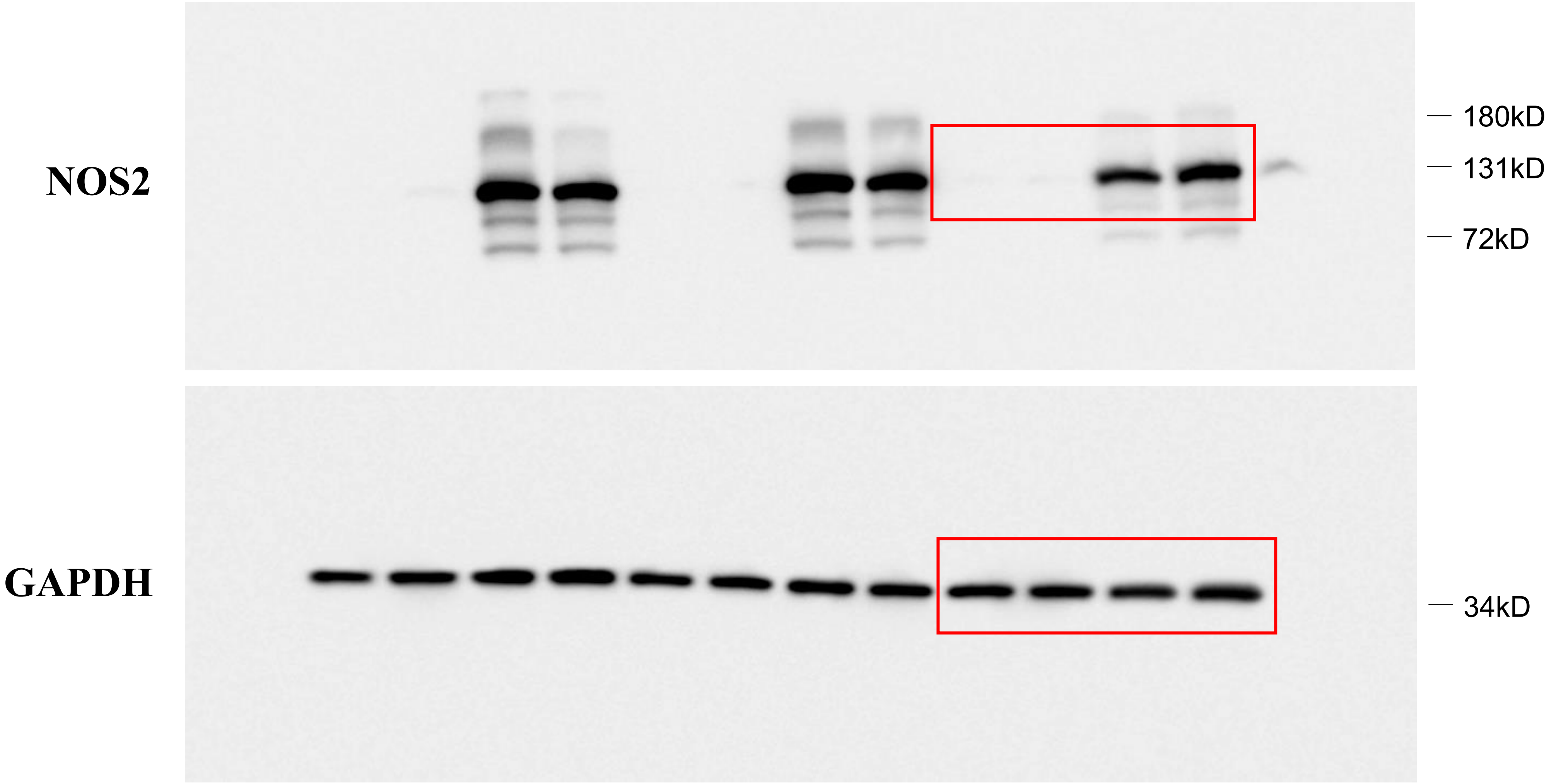

Figure 4E

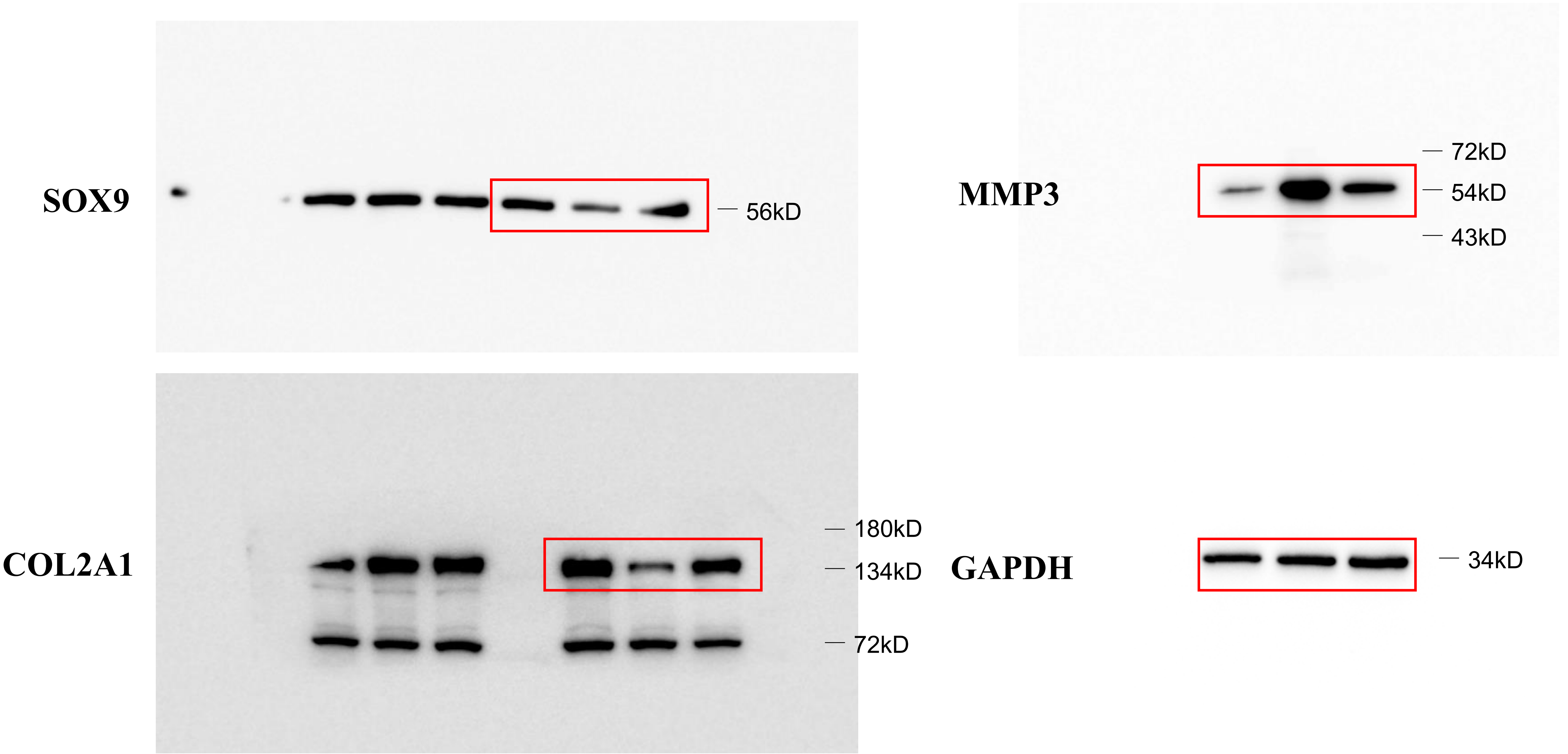

SOX9
56kD
MMP3
72kD
54kD
43kD
COL2A1
180kD
134kD
72kD
GAPDH
34kD

Figure 4E

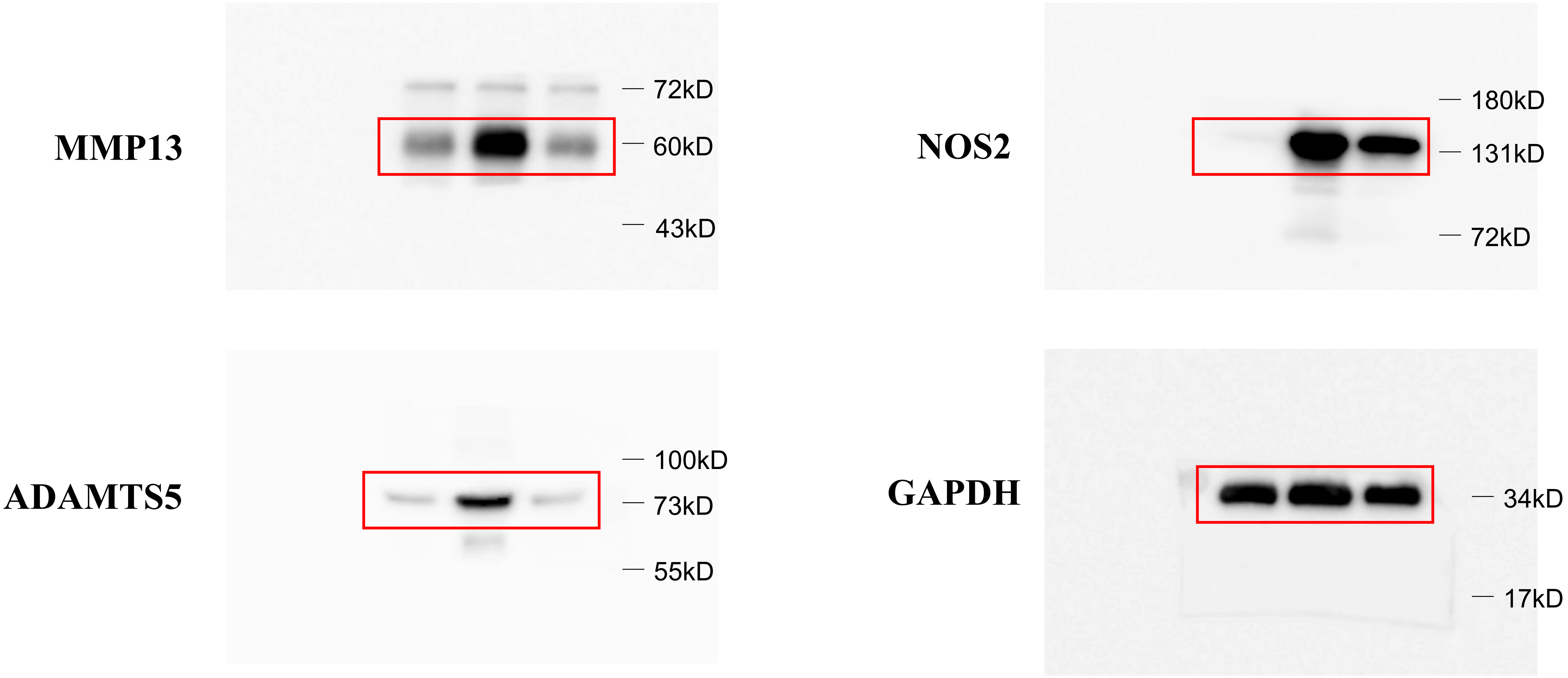
MMP13
72kD
60kD
43kD
NOS2
180kD
131kD
72kD
ADAMTS5
100kD
73kD
55kD
GAPDH
34kD
17kD

Figure 5L

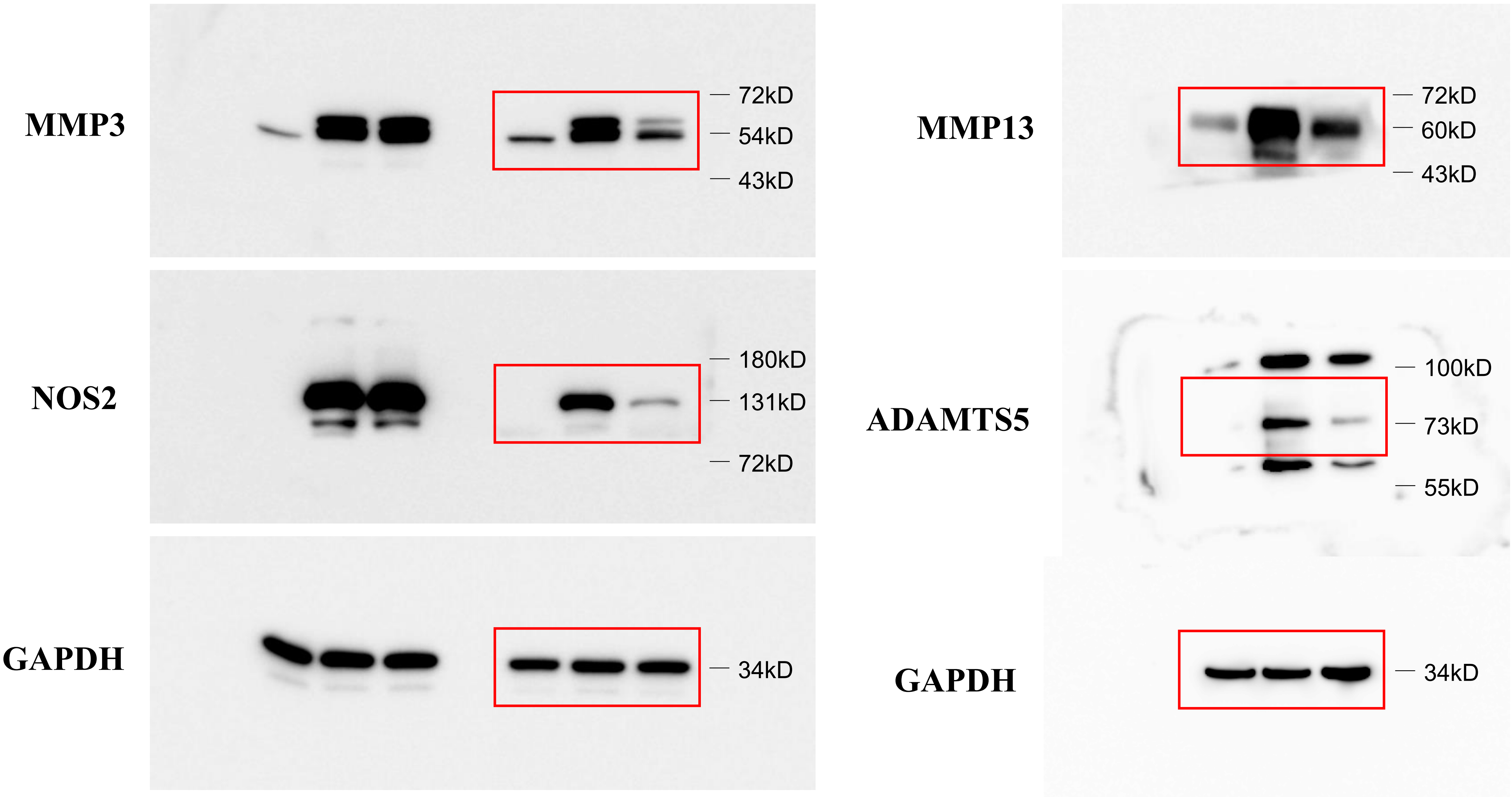

MMP3
72kD
54kD
43kD
NOS2
180kD
131kD
72kD
GAPDH
34kD
MMP13
72kD
60kD
43kD
ADAMTS5
100kD
73kD
55kD
GAPDH
34kD

Figure 6C

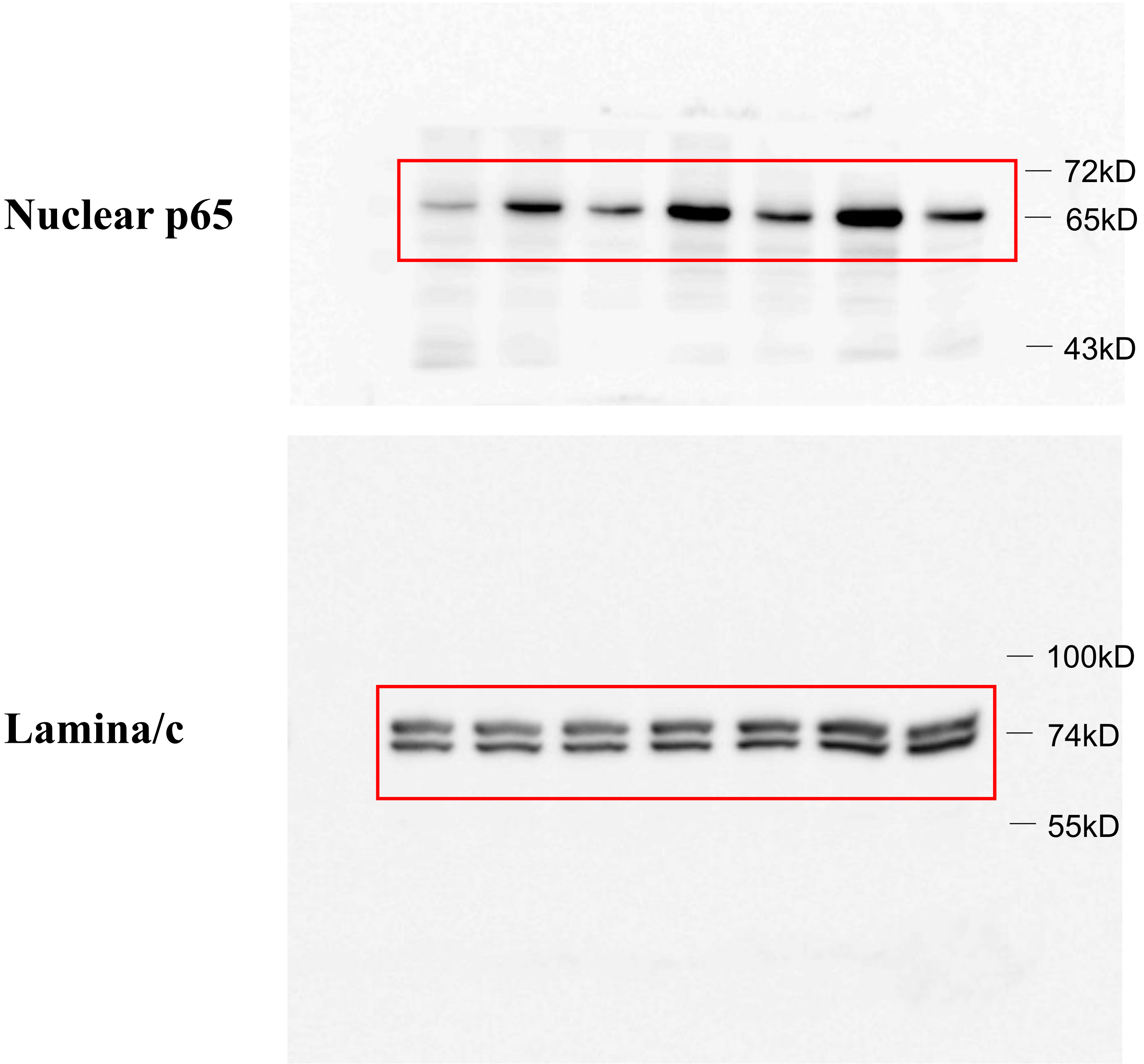

Figure 6C

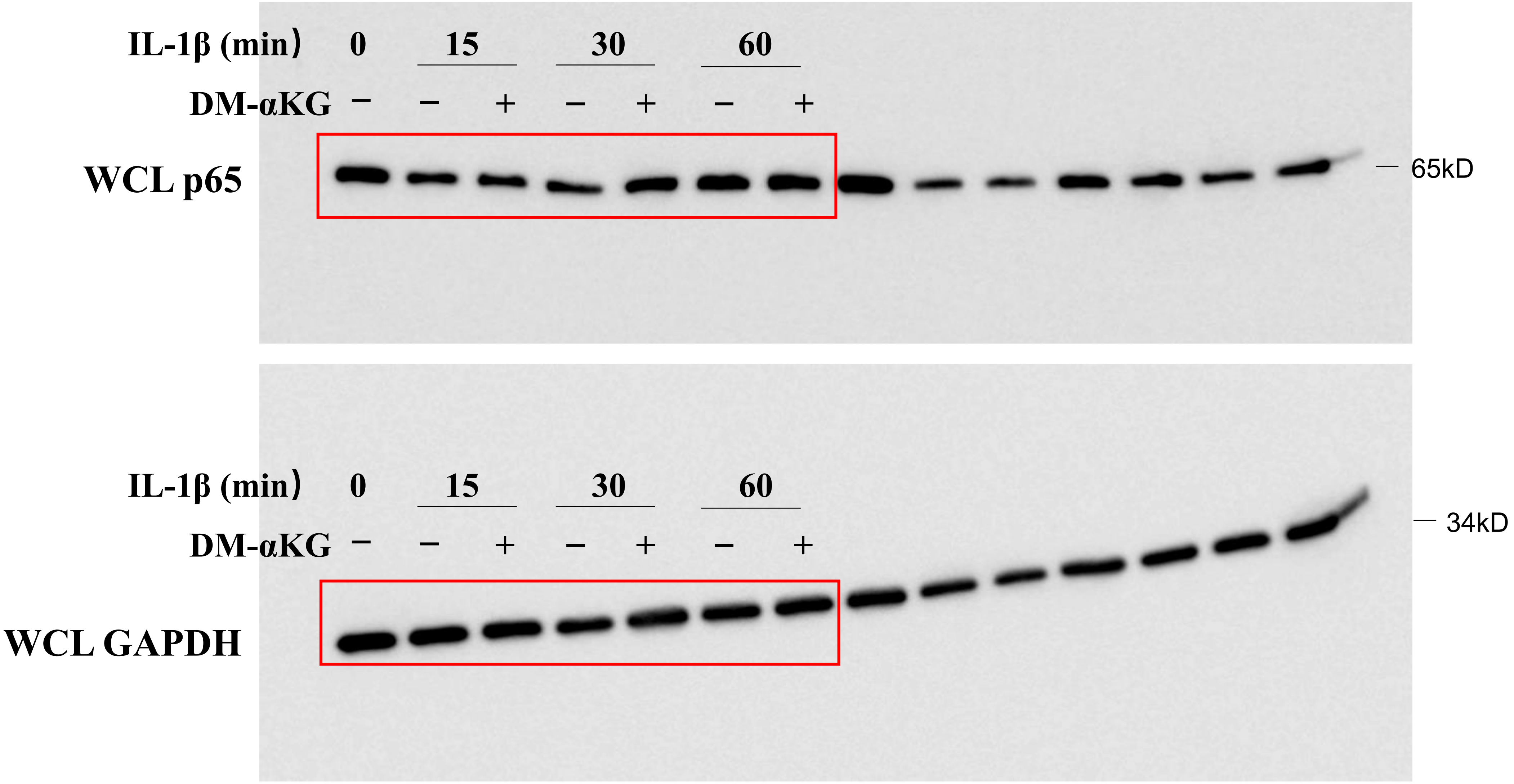
IL-1β (min) 0 15 30 60
DM-αKG − − + − + − +
WCL p65
65kD
IL-1β (min) 0 15 30 60
DM-αKG − − + − + − +
WCL GAPDH
34kD

Figure 6C

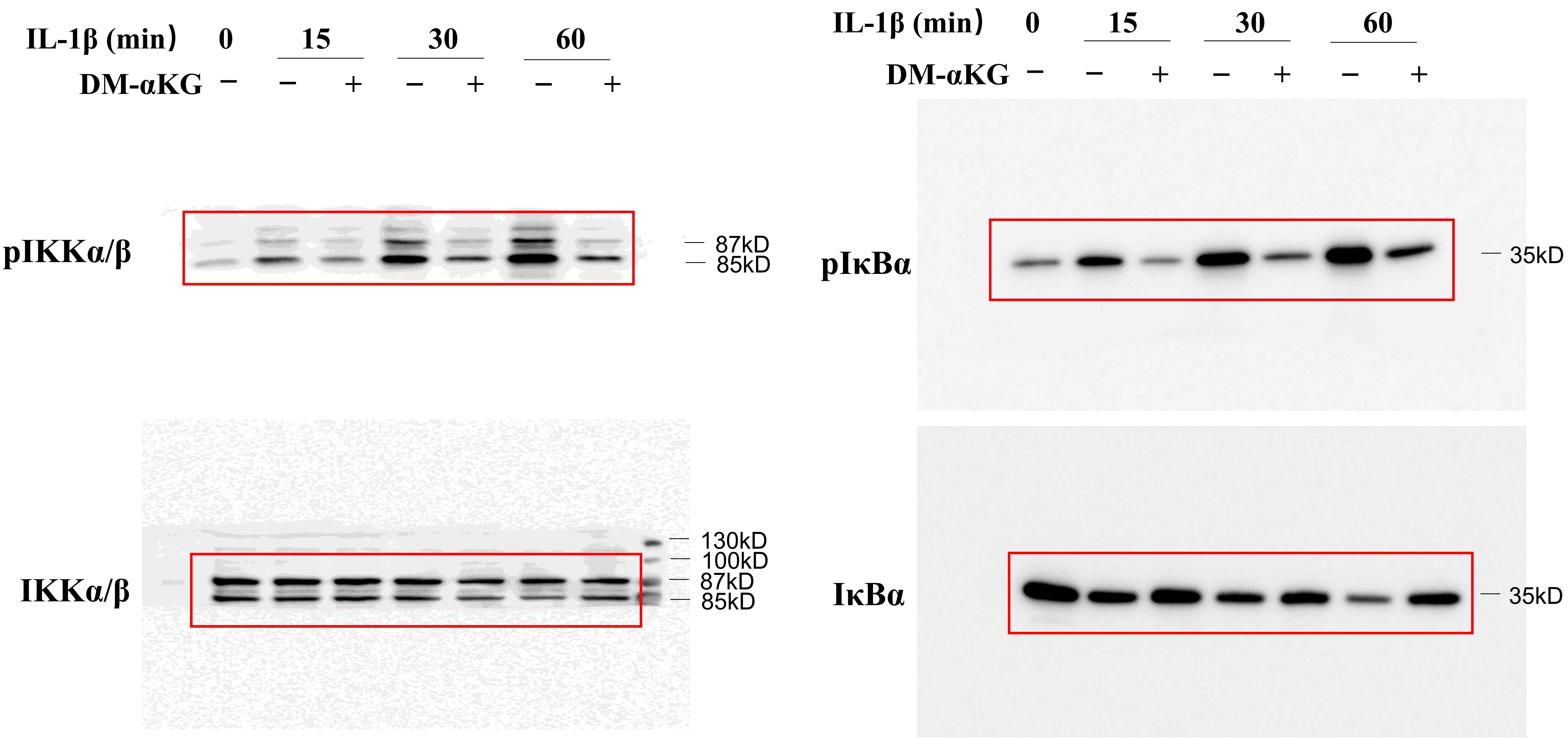
IL-1β (min) 0 15 30 60
DM-αKG − − + − + − +
pIKKα/β
87kD
85kD
IKKα/β
130kD
100kD
87kD
85kD
IL-1β (min) 0 15 30 60
DM-αKG − − + − + − +
pIκBα
35kD
IκBα
35kD

Figure 6C

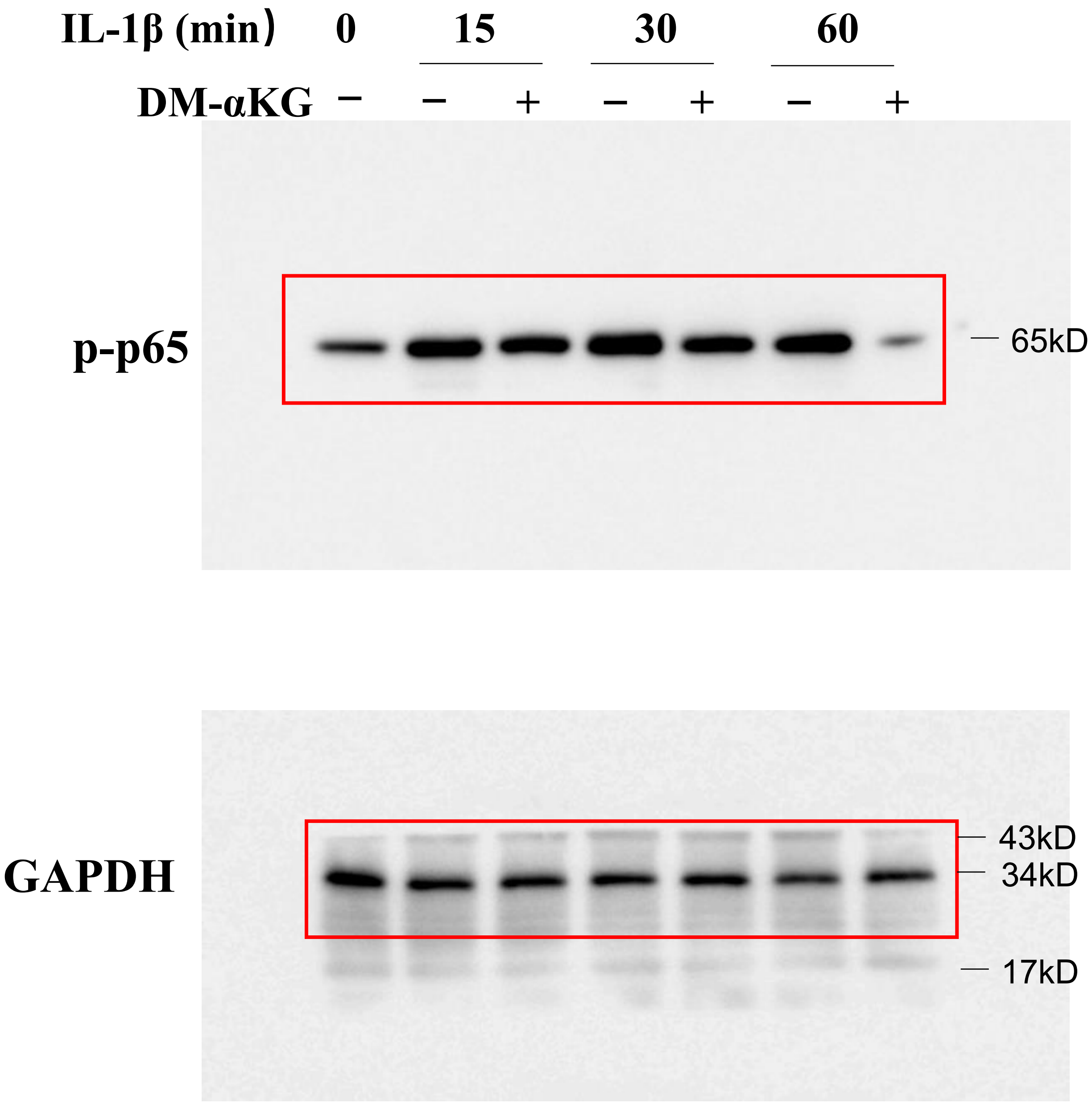

Figure 6D

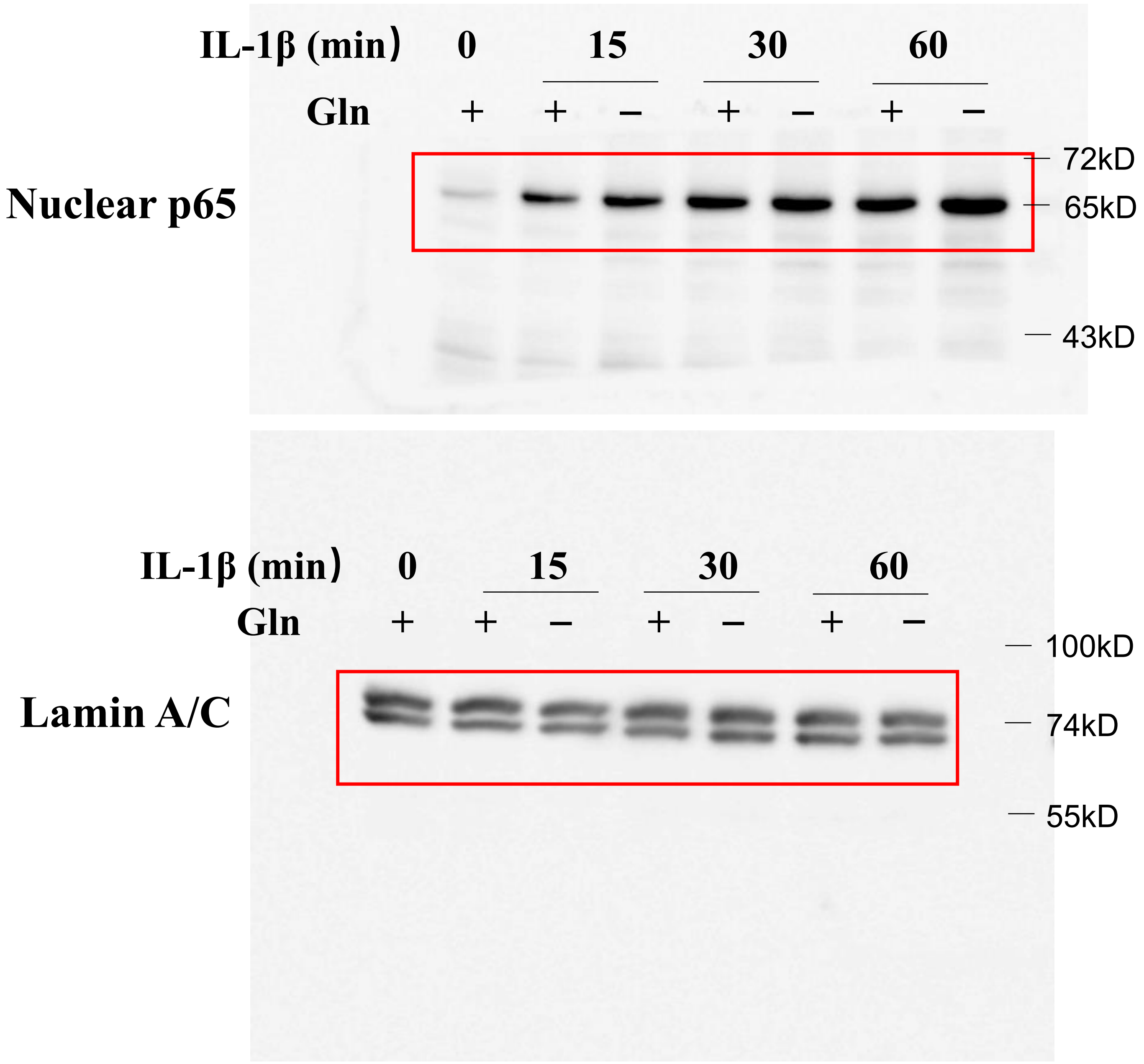
IL-1β (min) 0 15 30 60
Gln + + – + – + –
Nuclear p65
72kD
65kD
43kD
IL-1β (min) 0 15 30 60
Gln + + – + – + –
Lamin A/C
100kD
74kD
55kD

Figure 6D

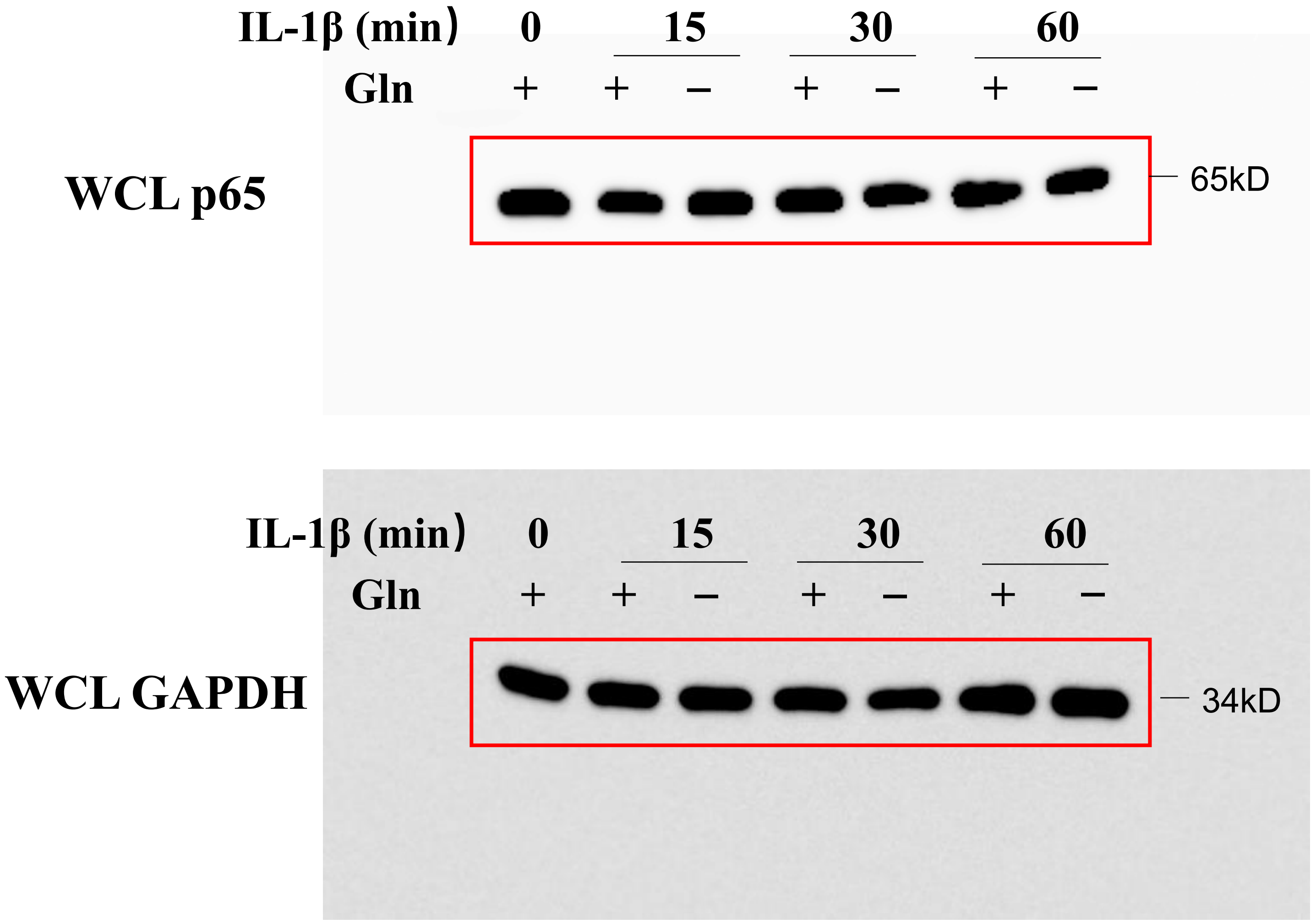

IL-1β (min) 0 15 30 60
Gln + + − + − + −
WCL p65
65kD
IL-1β (min) 0 15 30 60
Gln + + − + − + −
WCL GAPDH
34kD

Figure 6D

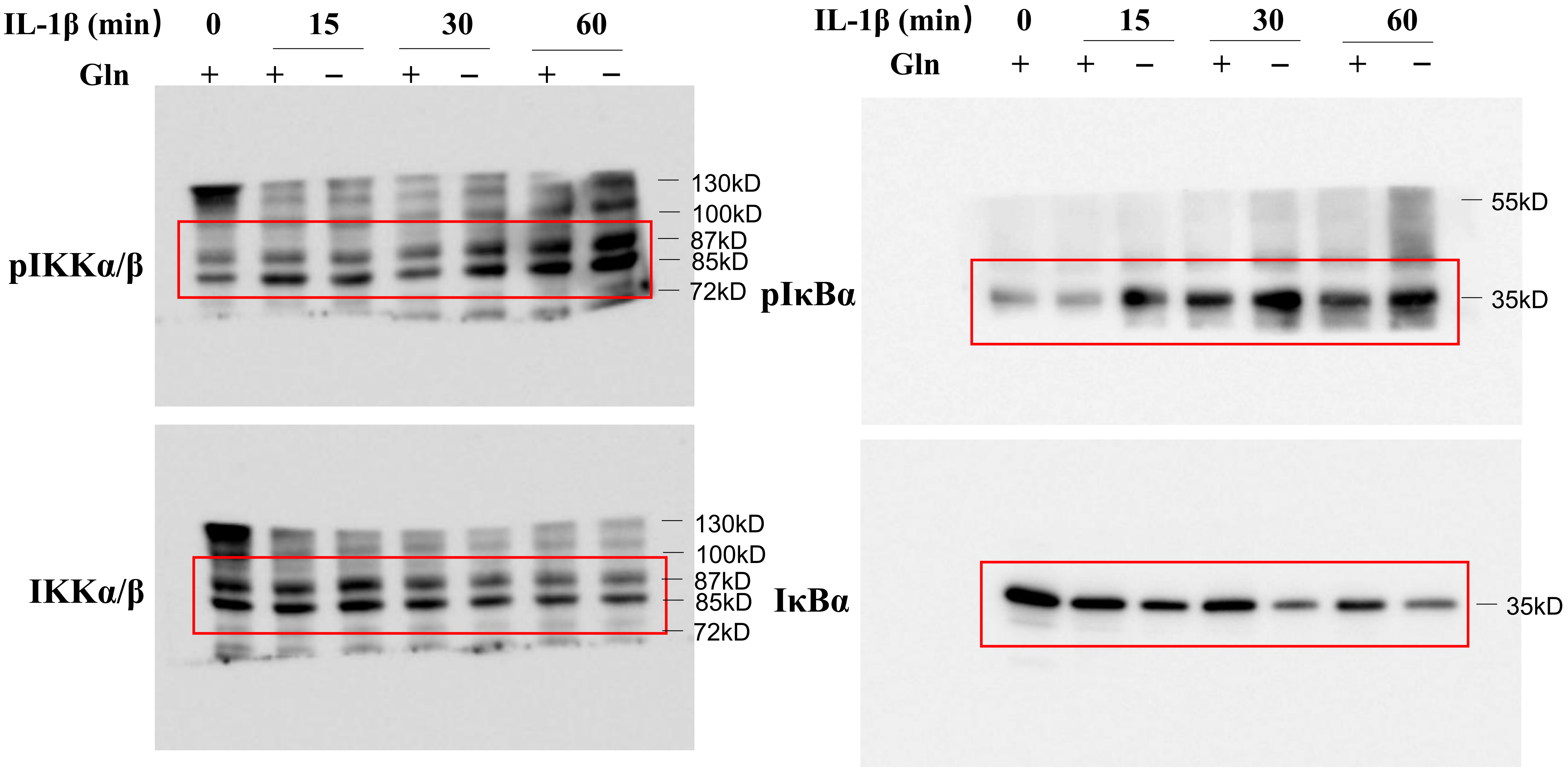

IL-1β (min) 0 15 30 60
Gln + + – + – + –
pIKKα/β
130kD
100kD
87kD
85kD
72kD
IKKα/β
130kD
100kD
87kD
85kD
72kD
IL-1β (min) 0 15 30 60
Gln + + – + – + –
pIκBα
55kD
35kD
IκBα
35kD

Figure 6D

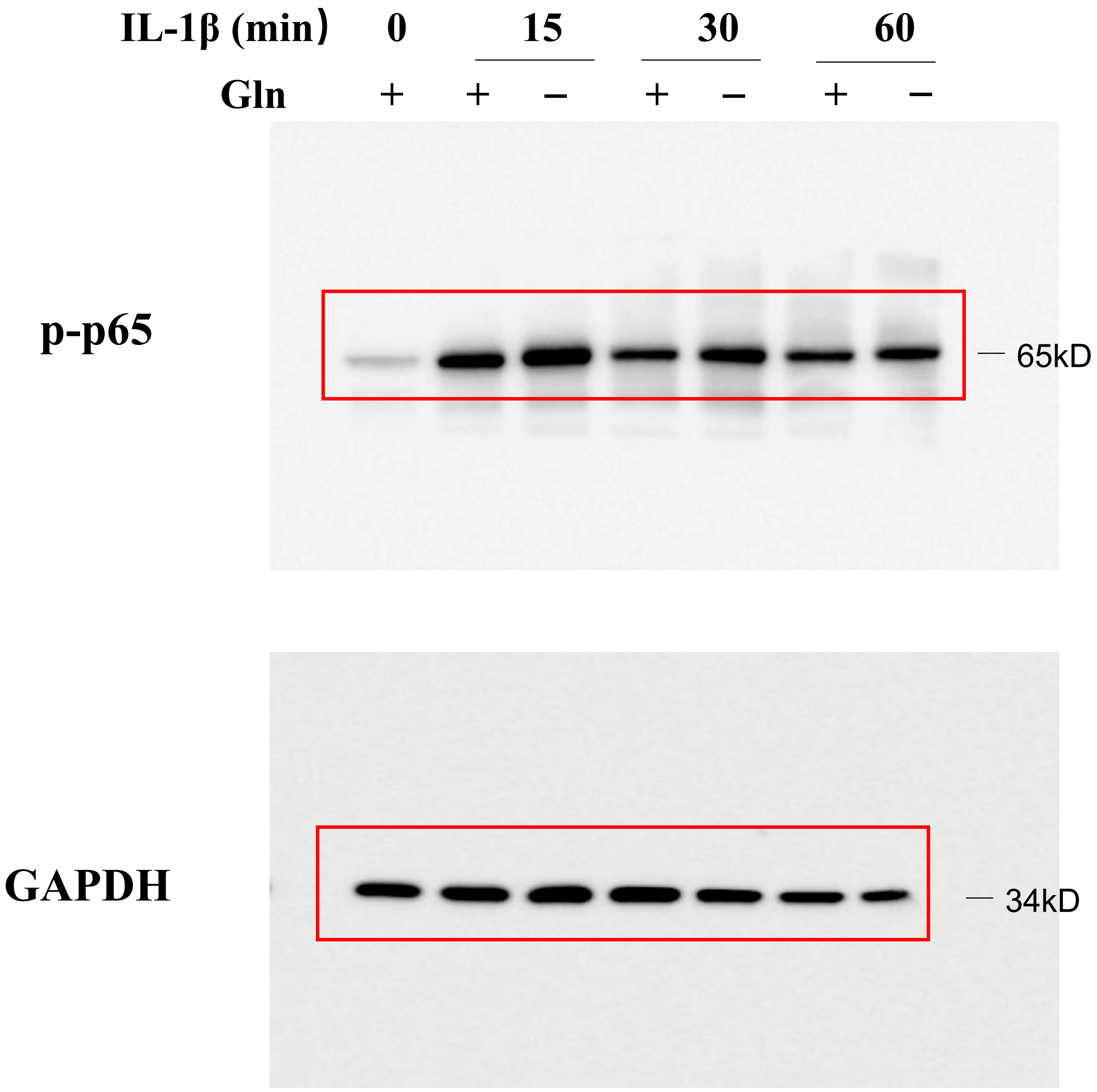

Figure 6E

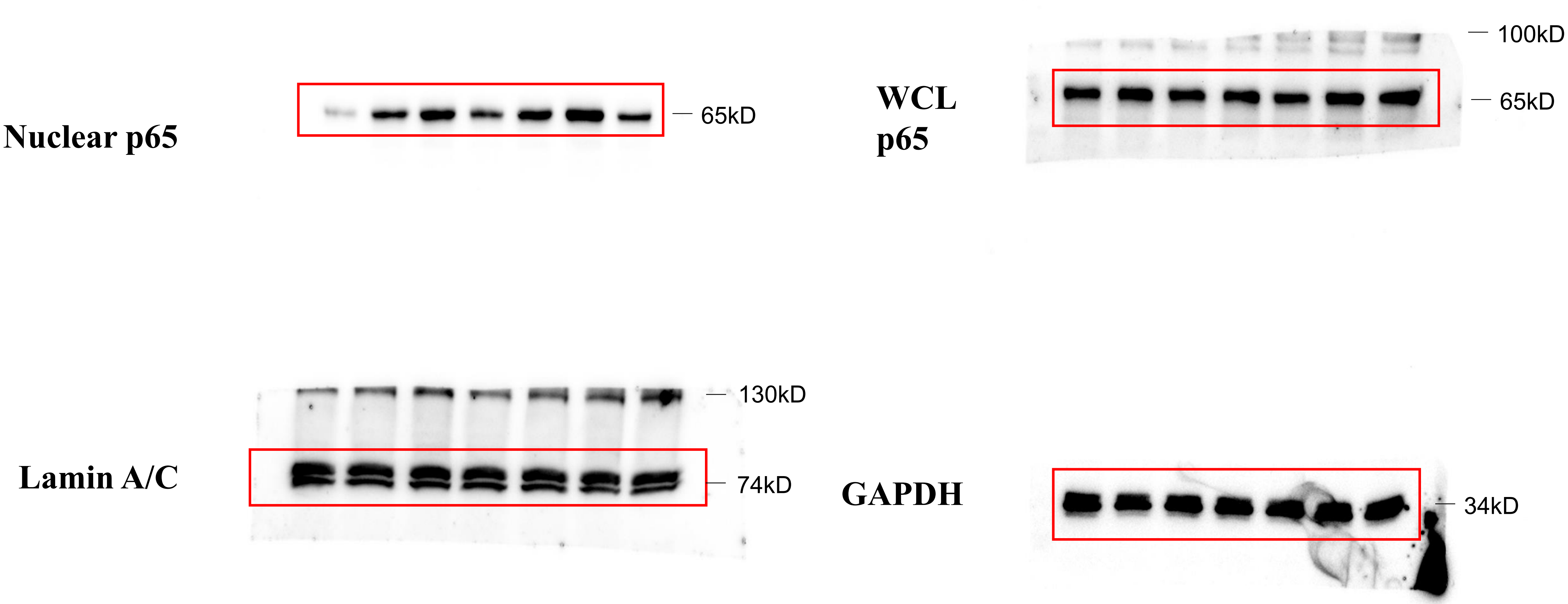
Nuclear p65
65kD
WCL
p65
100kD
65kD
Lamin A/C
130kD
74kD
GAPDH
34kD

Figure 6E

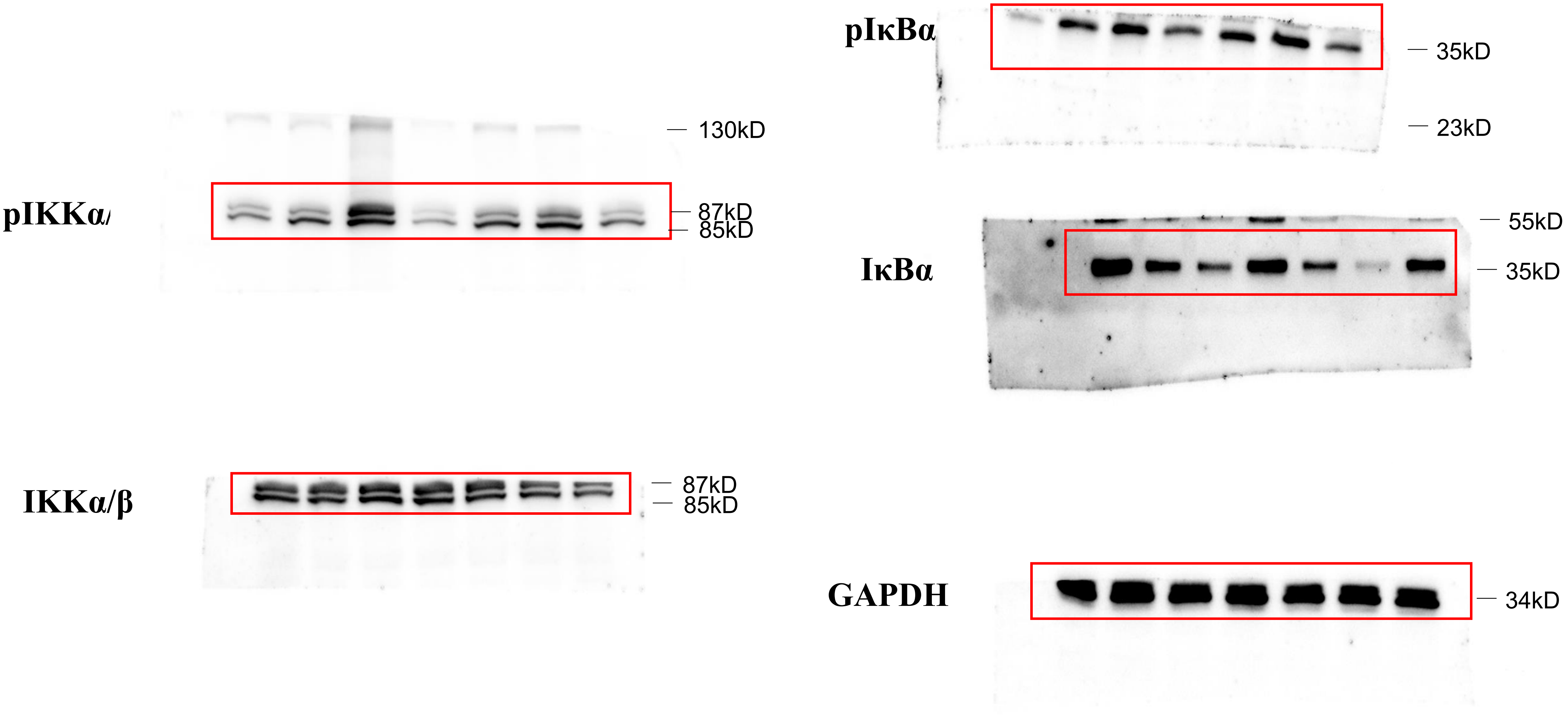
pIκBα
35kD
23kD
pIKKα/
130kD
87kD
85kD
IκBα
55kD
35kD
IKKα/β
87kD
85kD
GAPDH
34kD

Figure 6H

H3K27me3

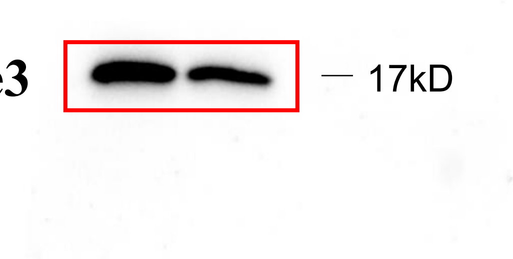

— 17kD

H3

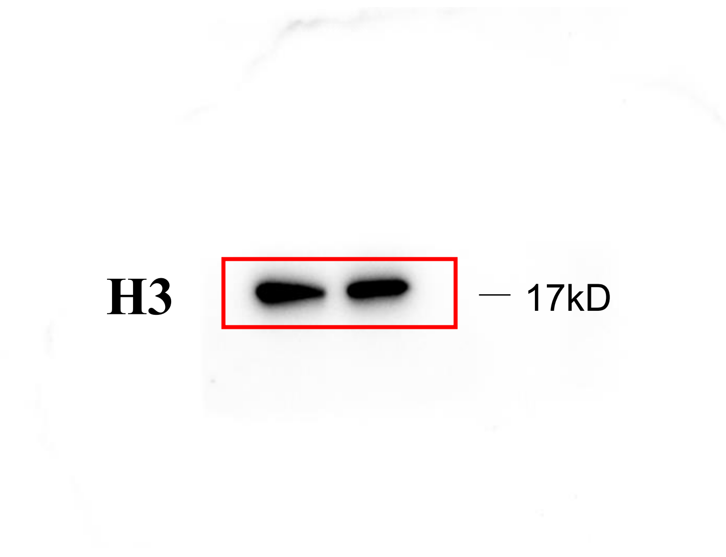

— 17kD

Figure 7A

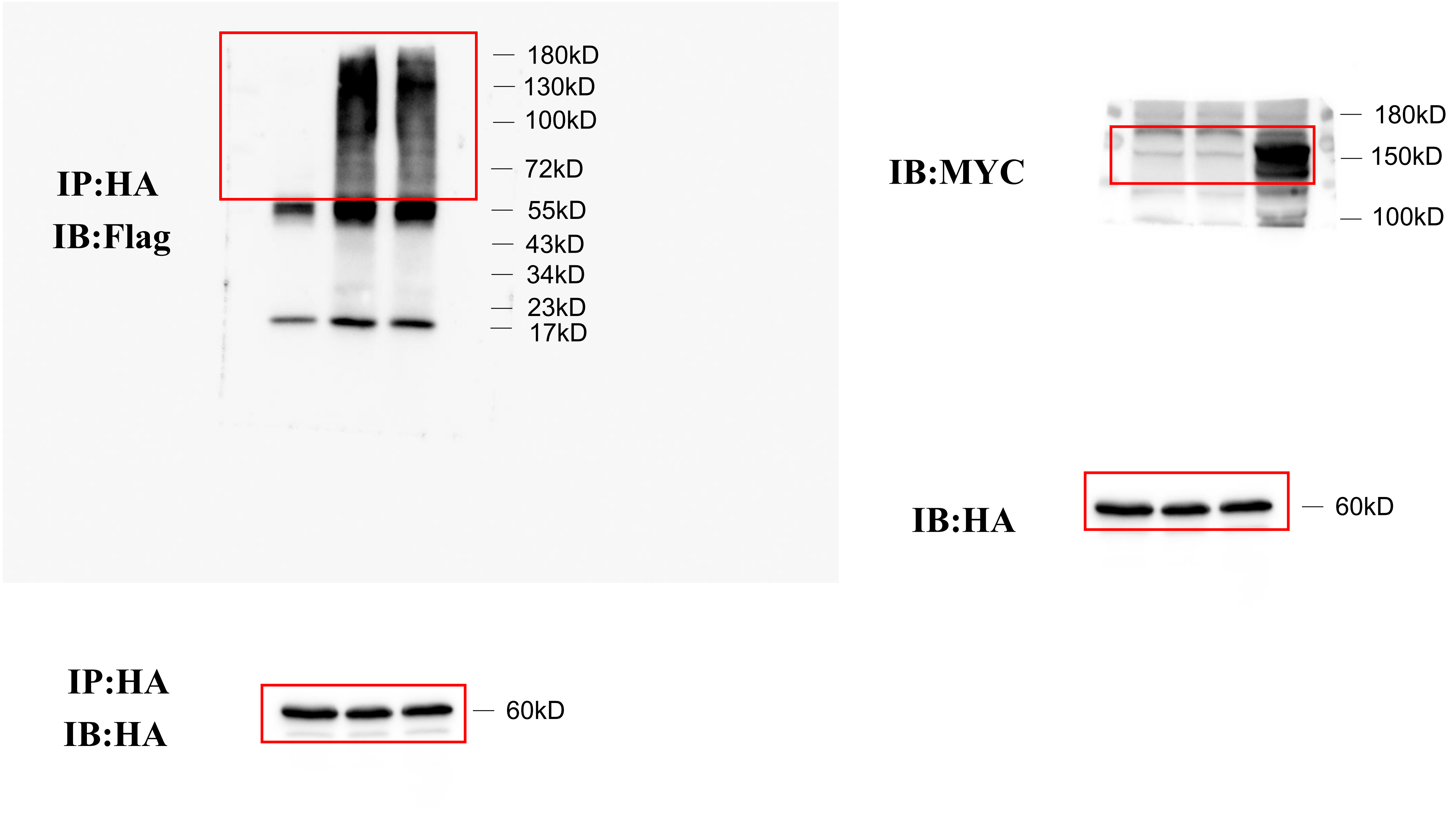
IP:HA
IB:Flag
180kD
130kD
100kD
72kD
55kD
43kD
34kD
23kD
17kD
IB:MYC
180kD
150kD
100kD
IB:HA
60kD
IP:HA
IB:HA
60kD

Figure 7C

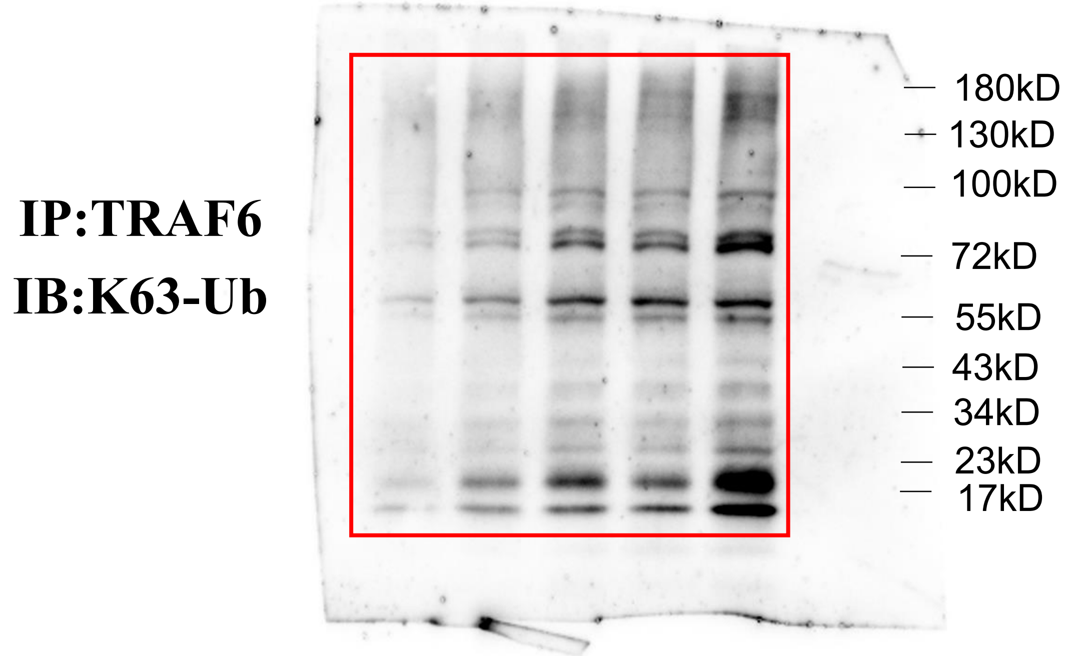

IP:TRAF6
IB:K63-Ub
180kD
130kD
100kD
72kD
55kD
43kD
34kD
23kD
17kD

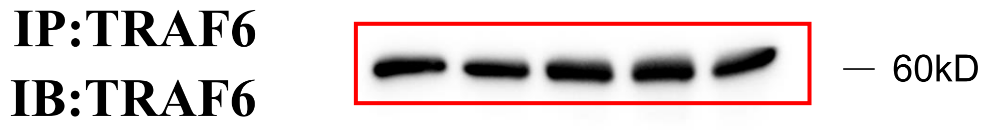

IP:TRAF6
IB:TRAF6
60kD

Figure 7D

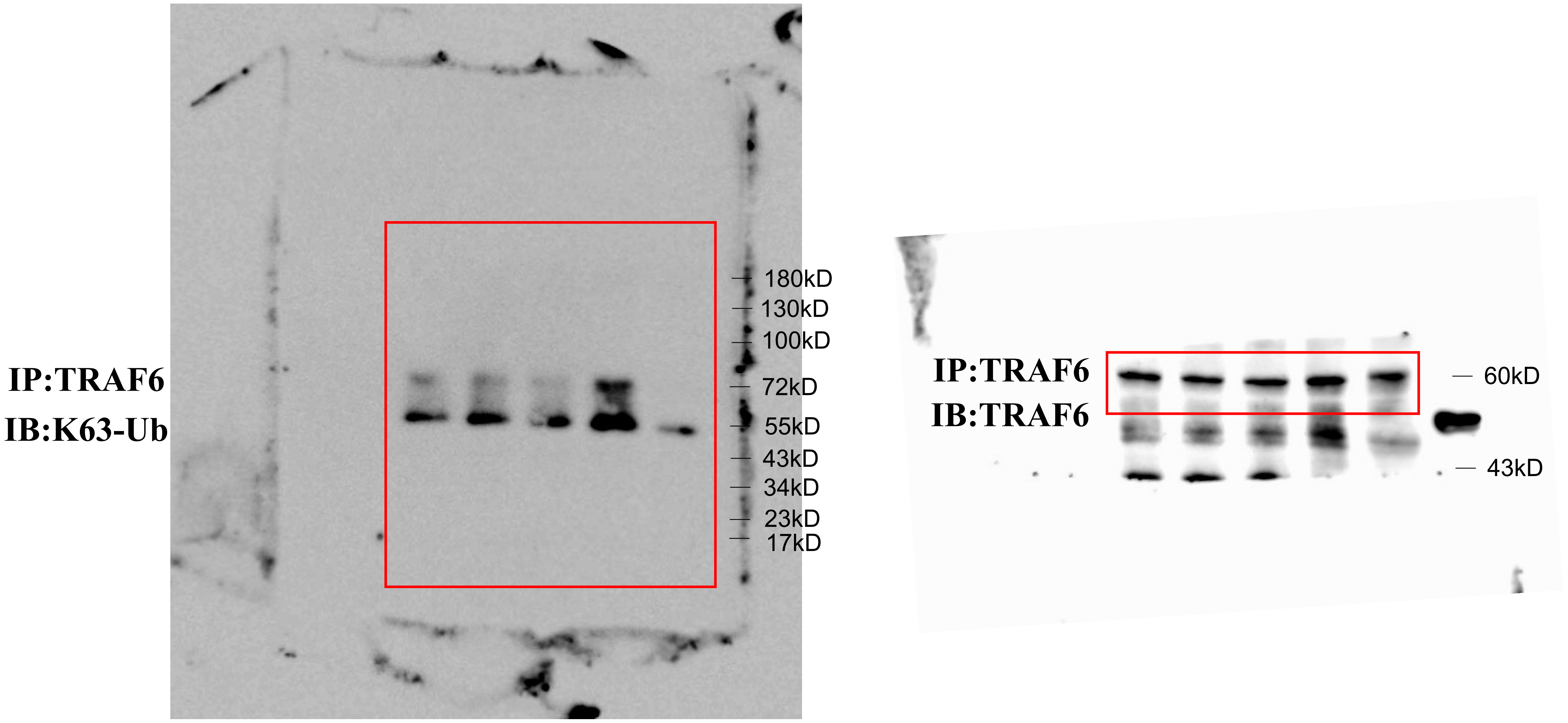

IP:TRAF6
IB:K63-Ub
180kD
130kD
100kD
72kD
55kD
43kD
34kD
23kD
17kD
IP:TRAF6
IB:TRAF6
60kD
43kD

Figure 7E

IP:TRAF6

IB:K63-Ub

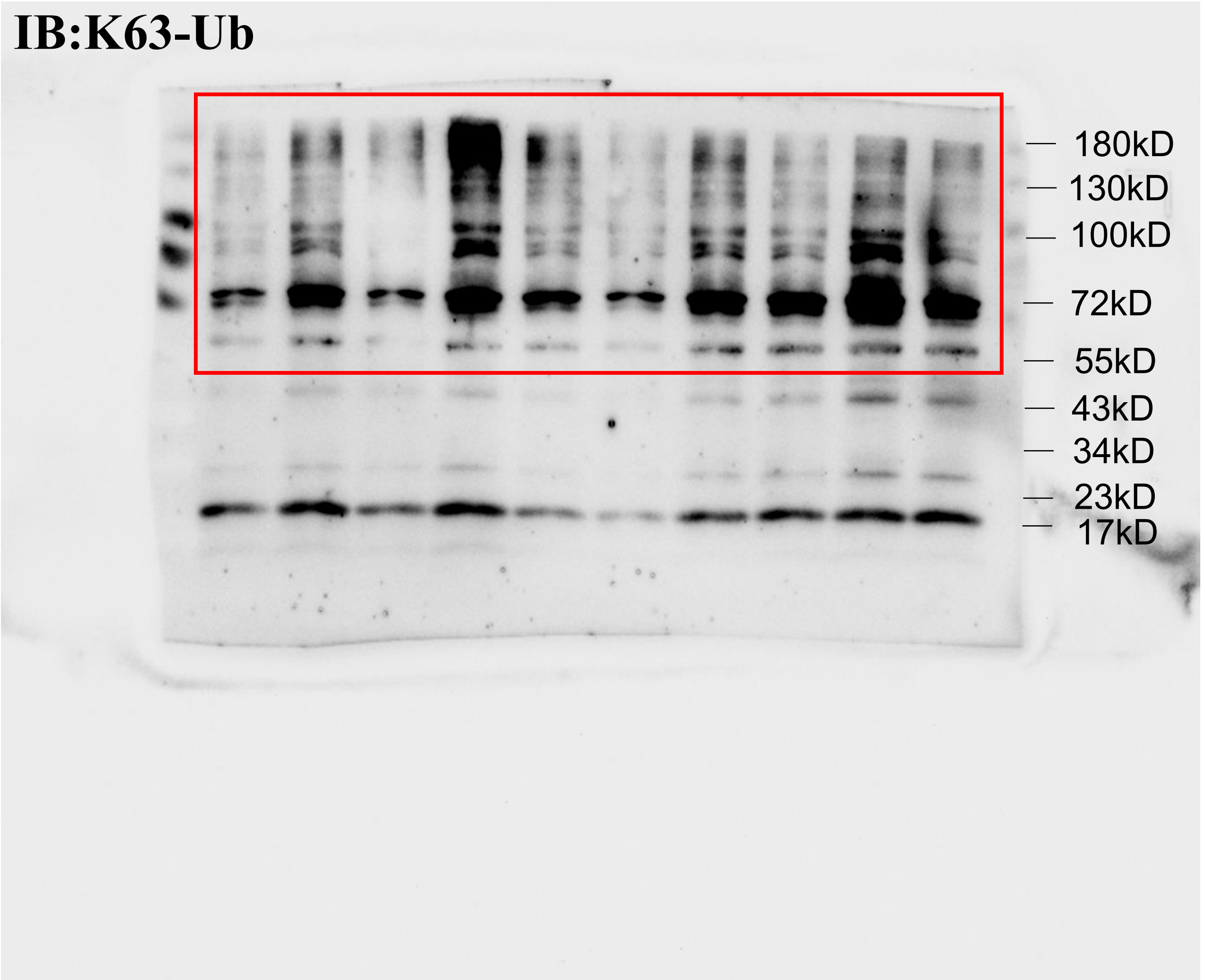

IP:TRAF6

IB:TRAF6

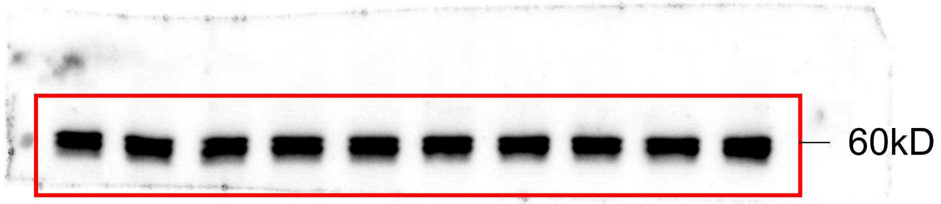

Figure 7H

SOX9

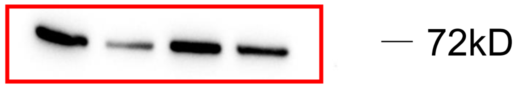

MMP3

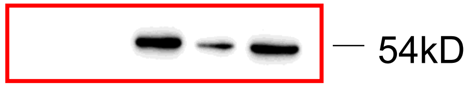

COL2A1

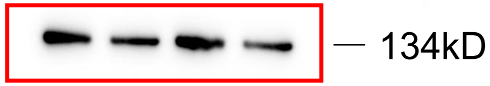

MMP13

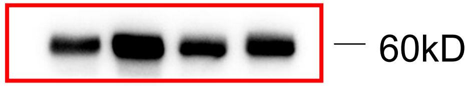

Figure 7H

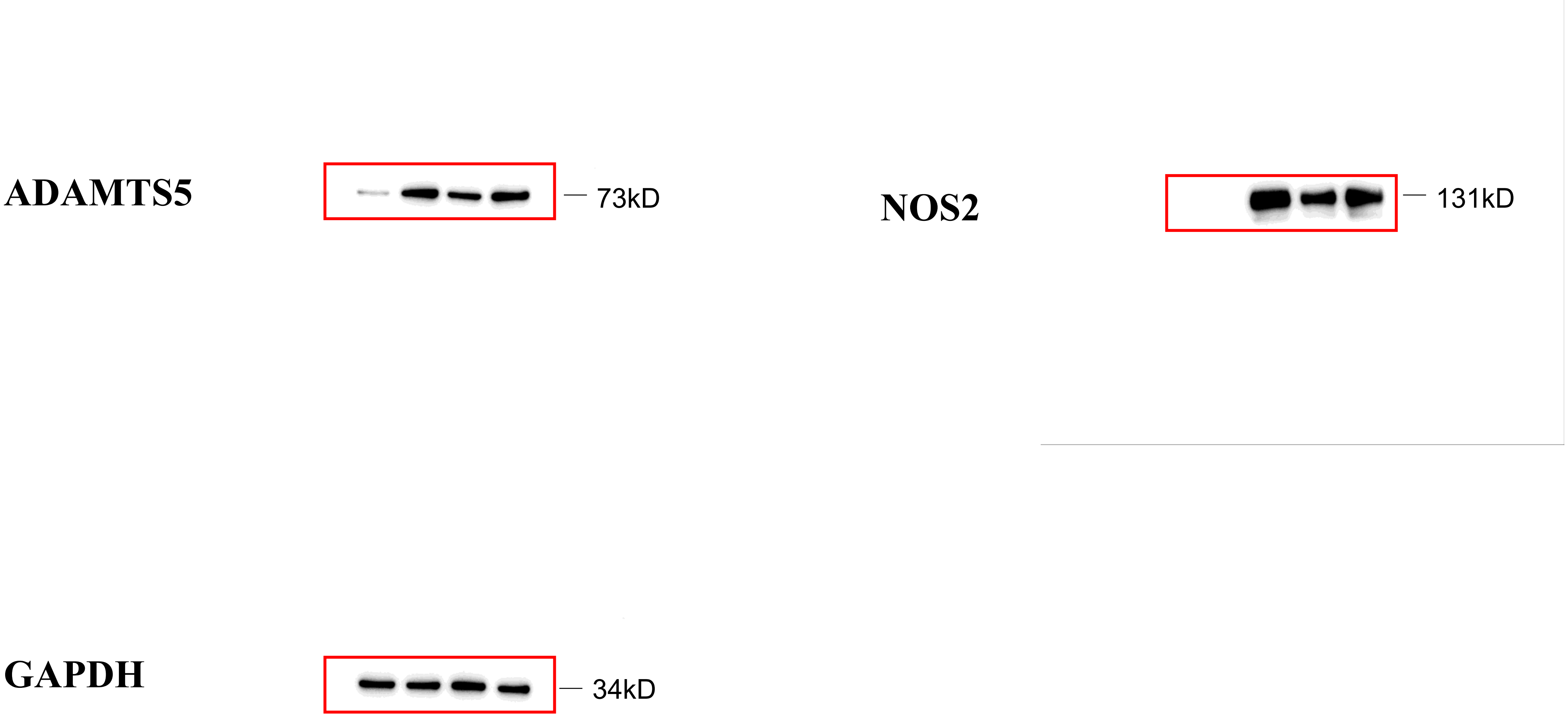

SFigure 3B

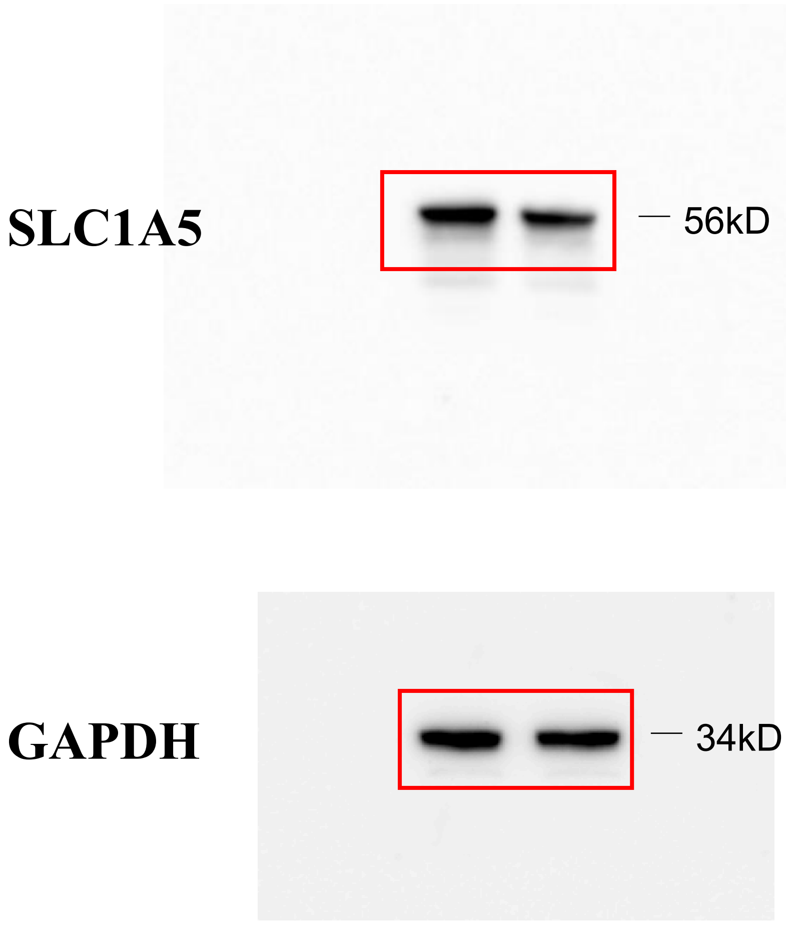

SFigure 3E

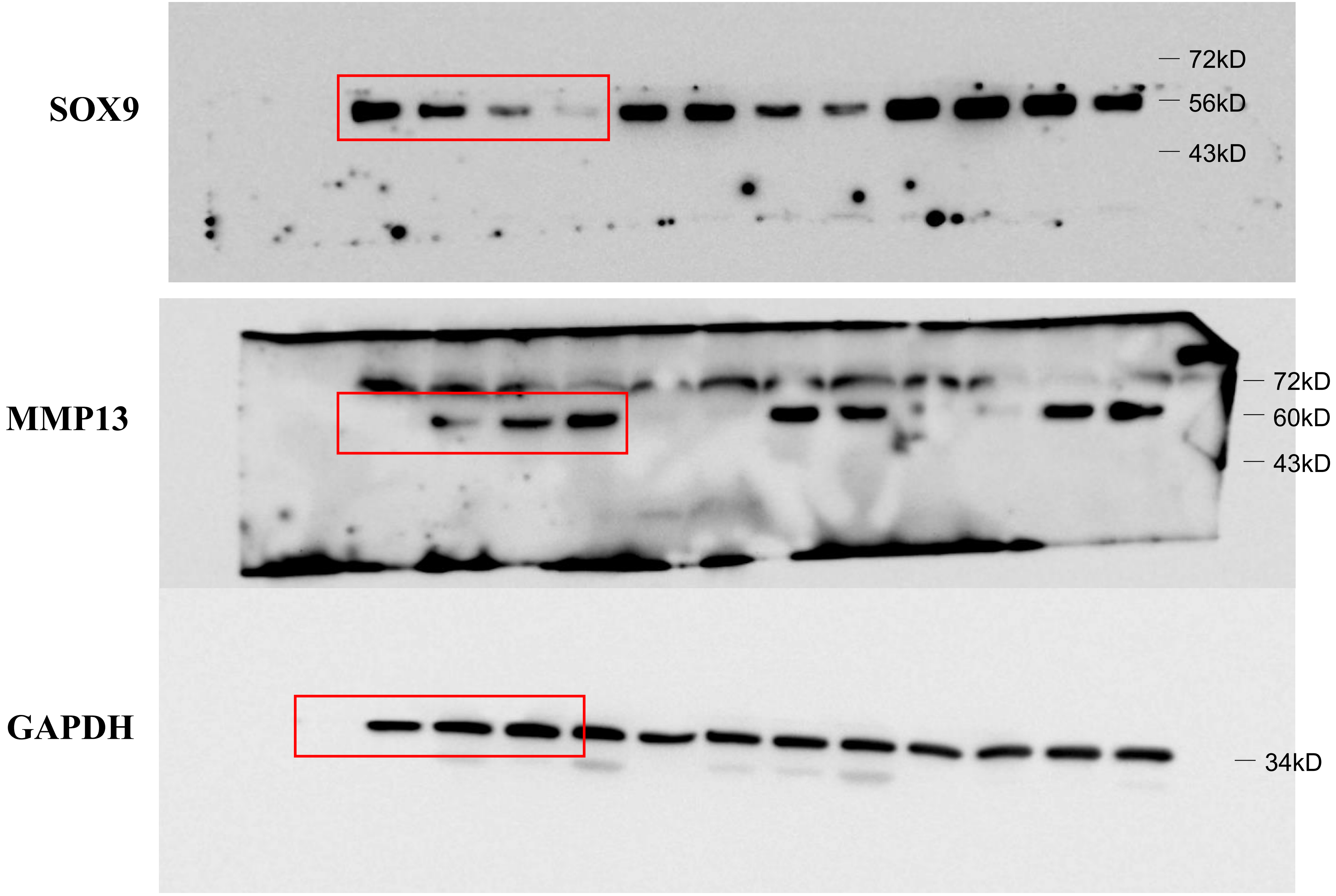

SFigure 3E

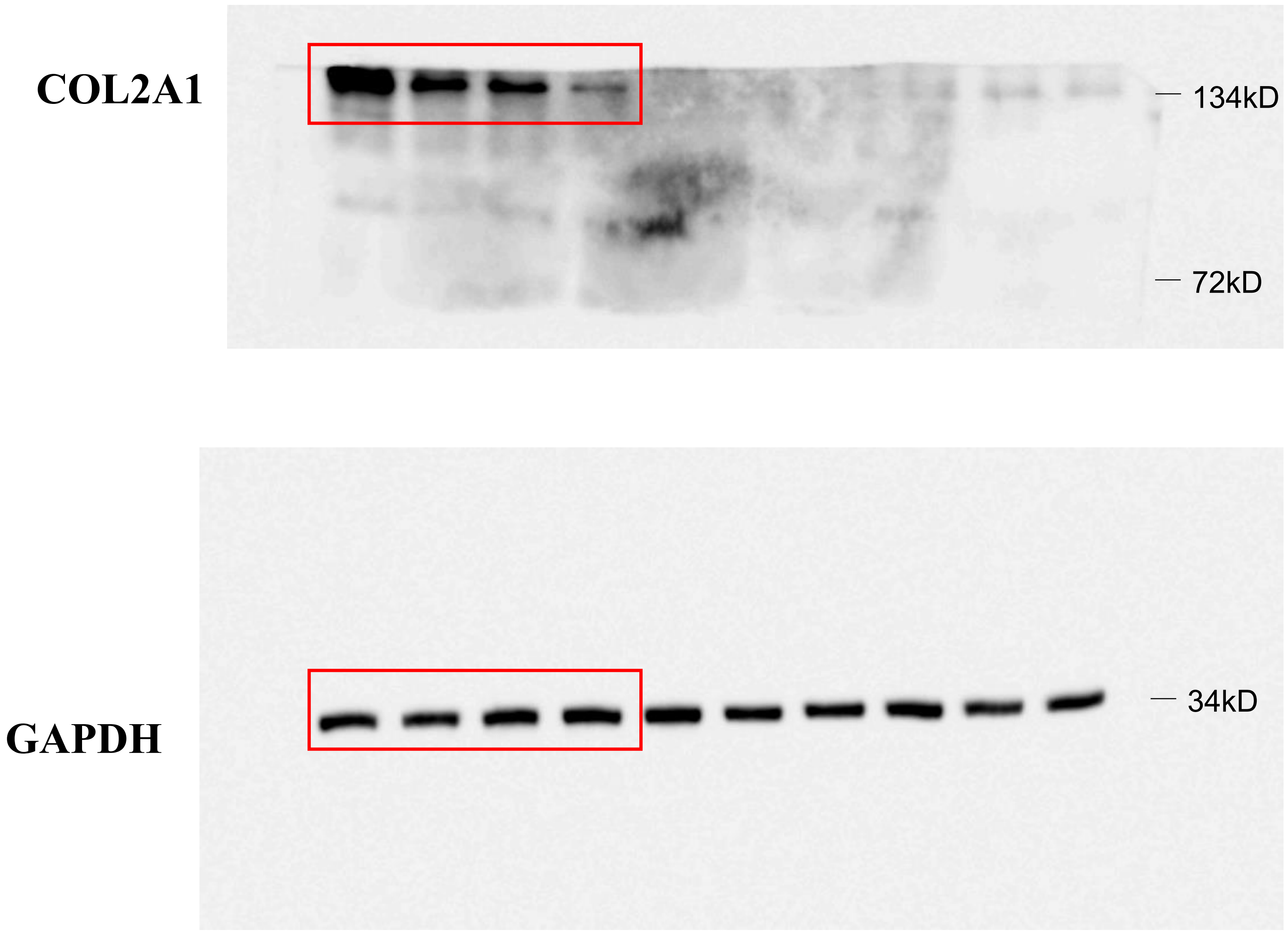

SFigure 3E

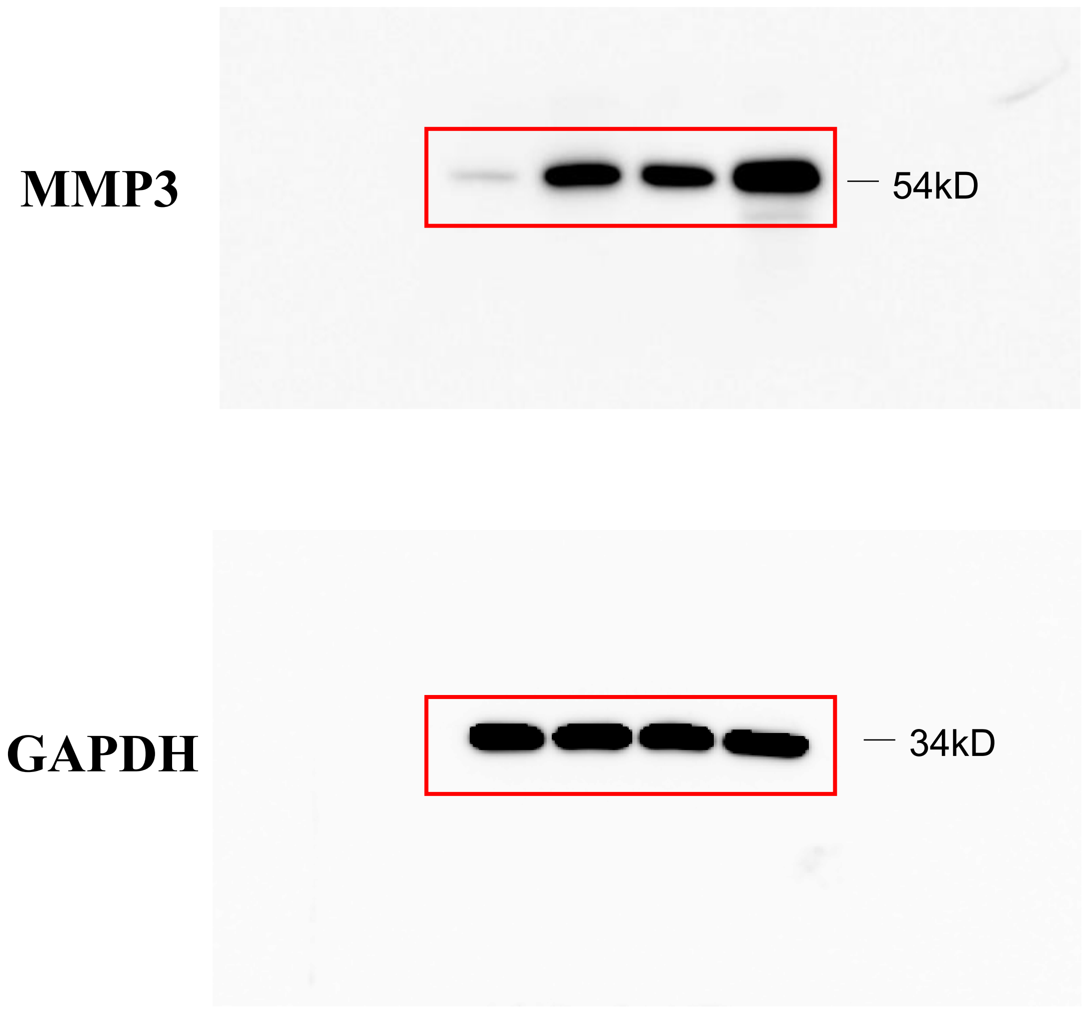

SFigure 3E

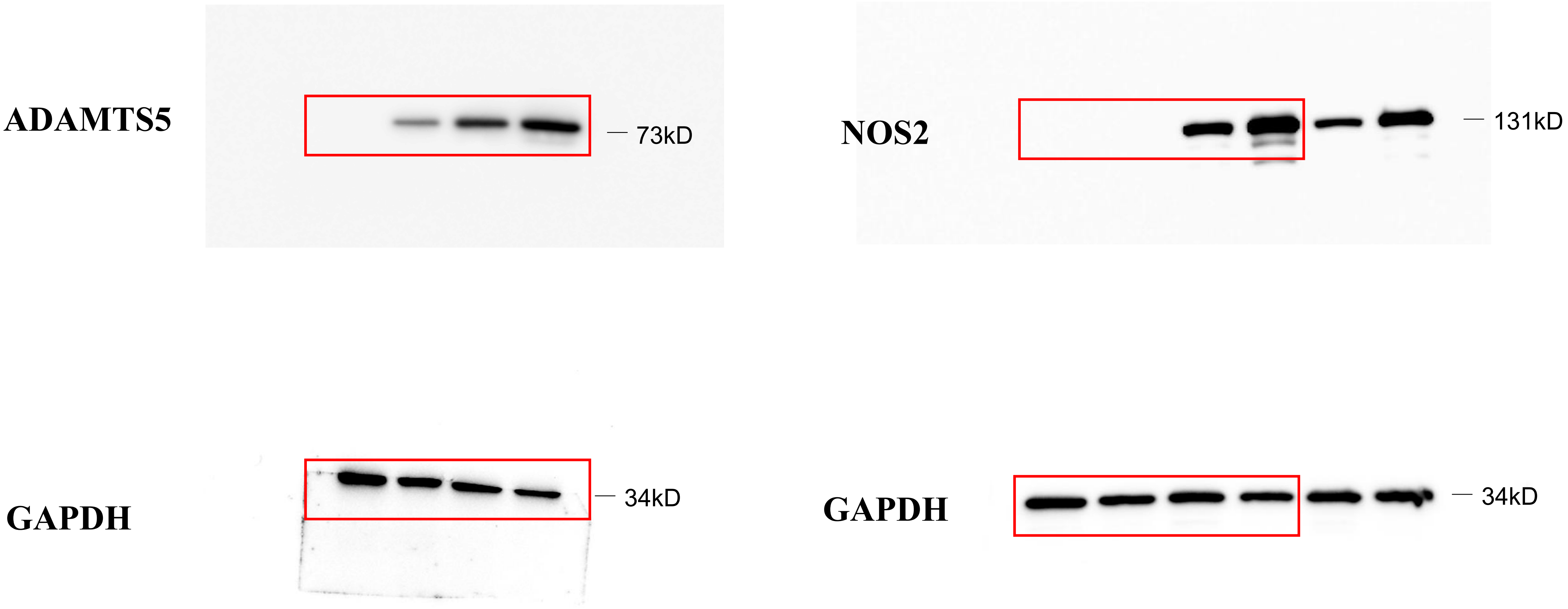

SFigure 3G

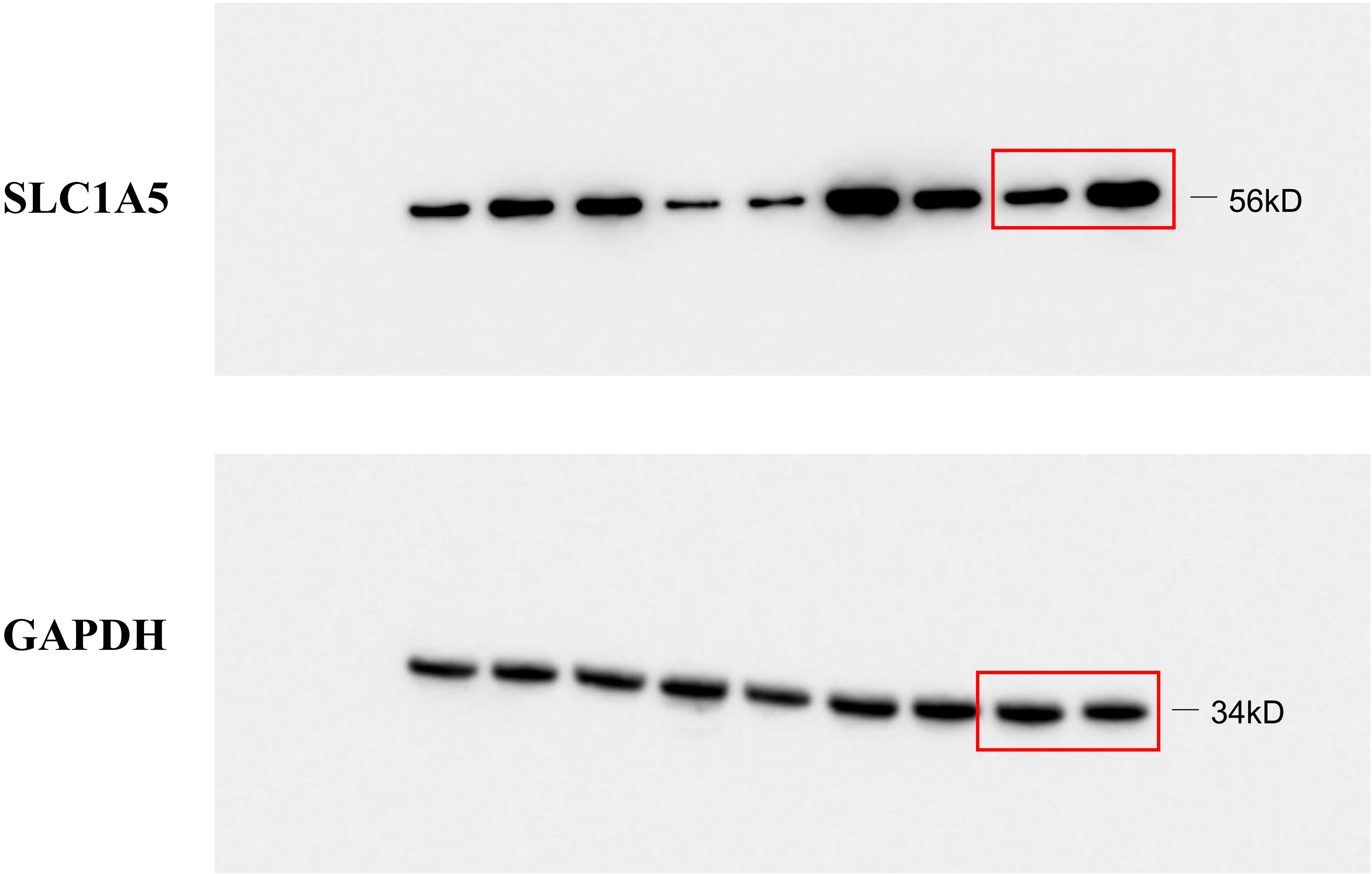

SFigure 3I

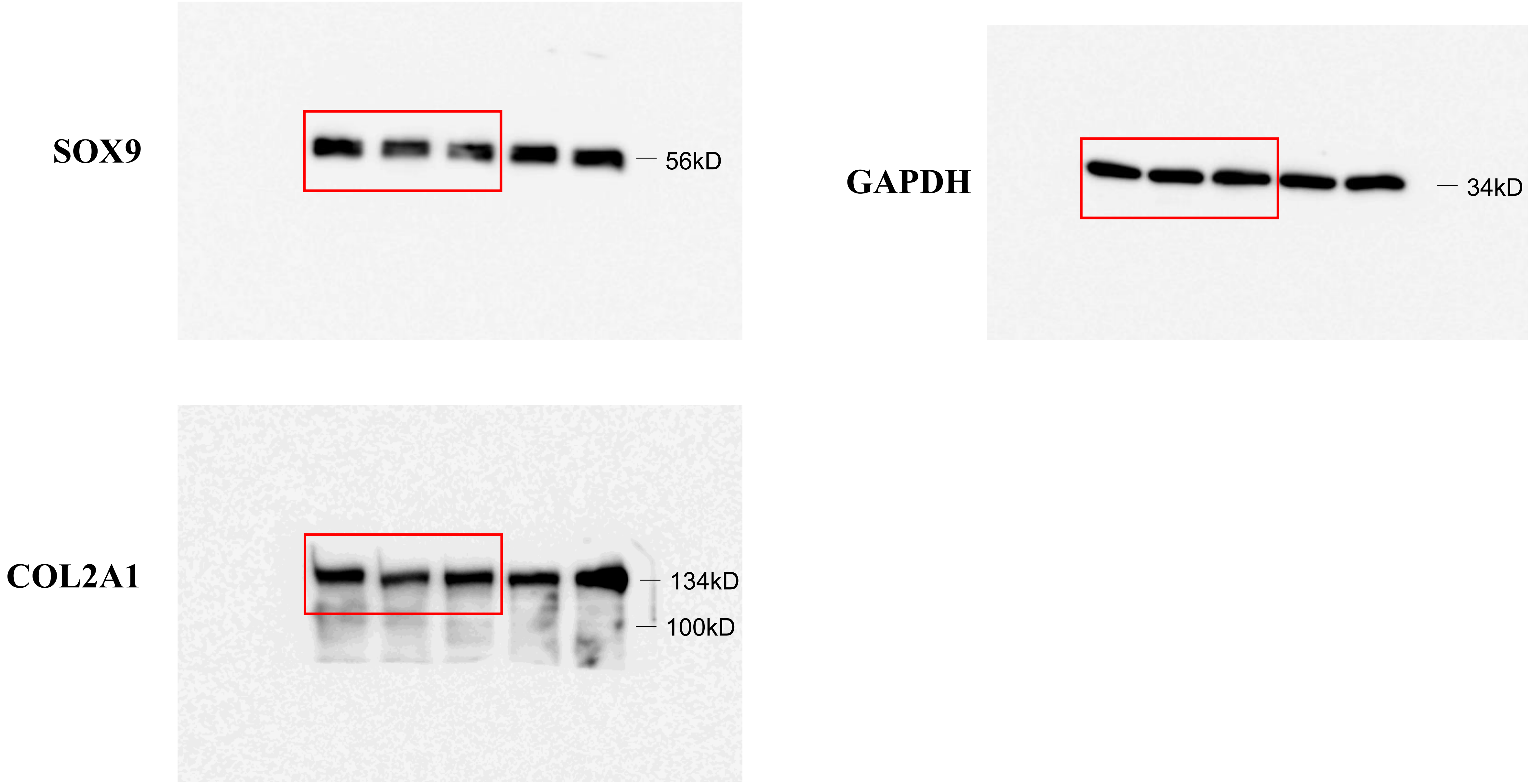

SFigure 3I

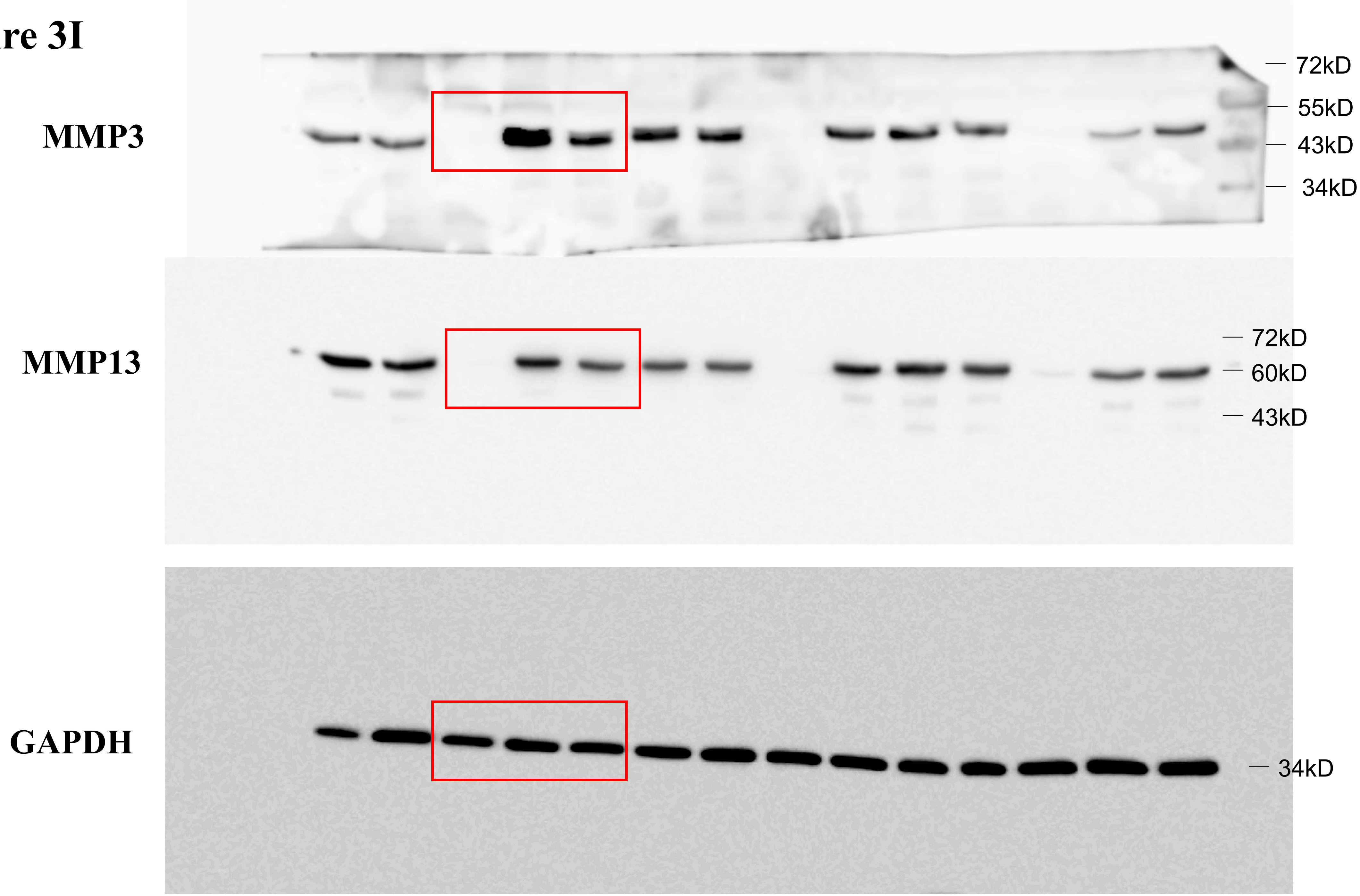

SFigure 3I

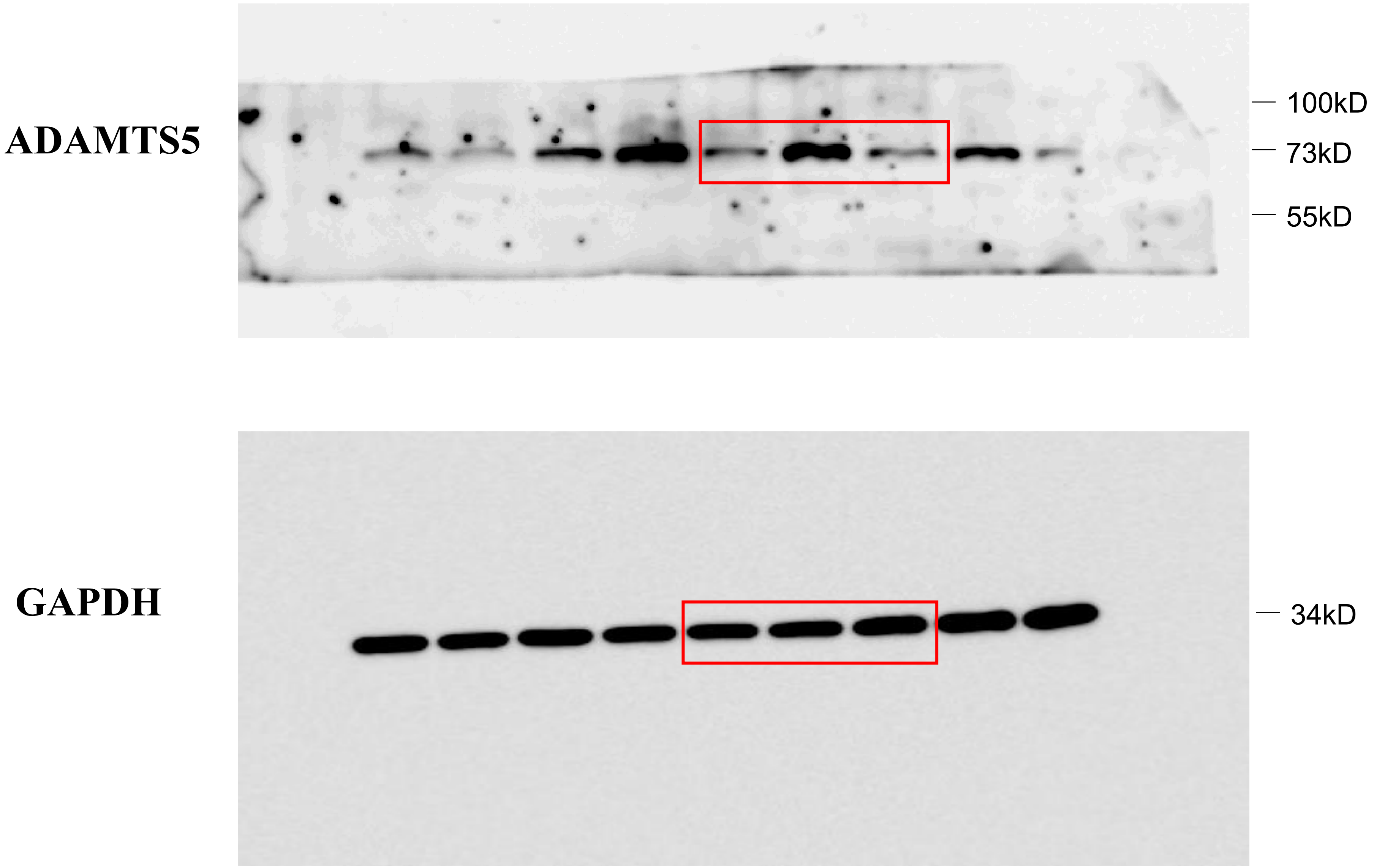

SFigure 3I

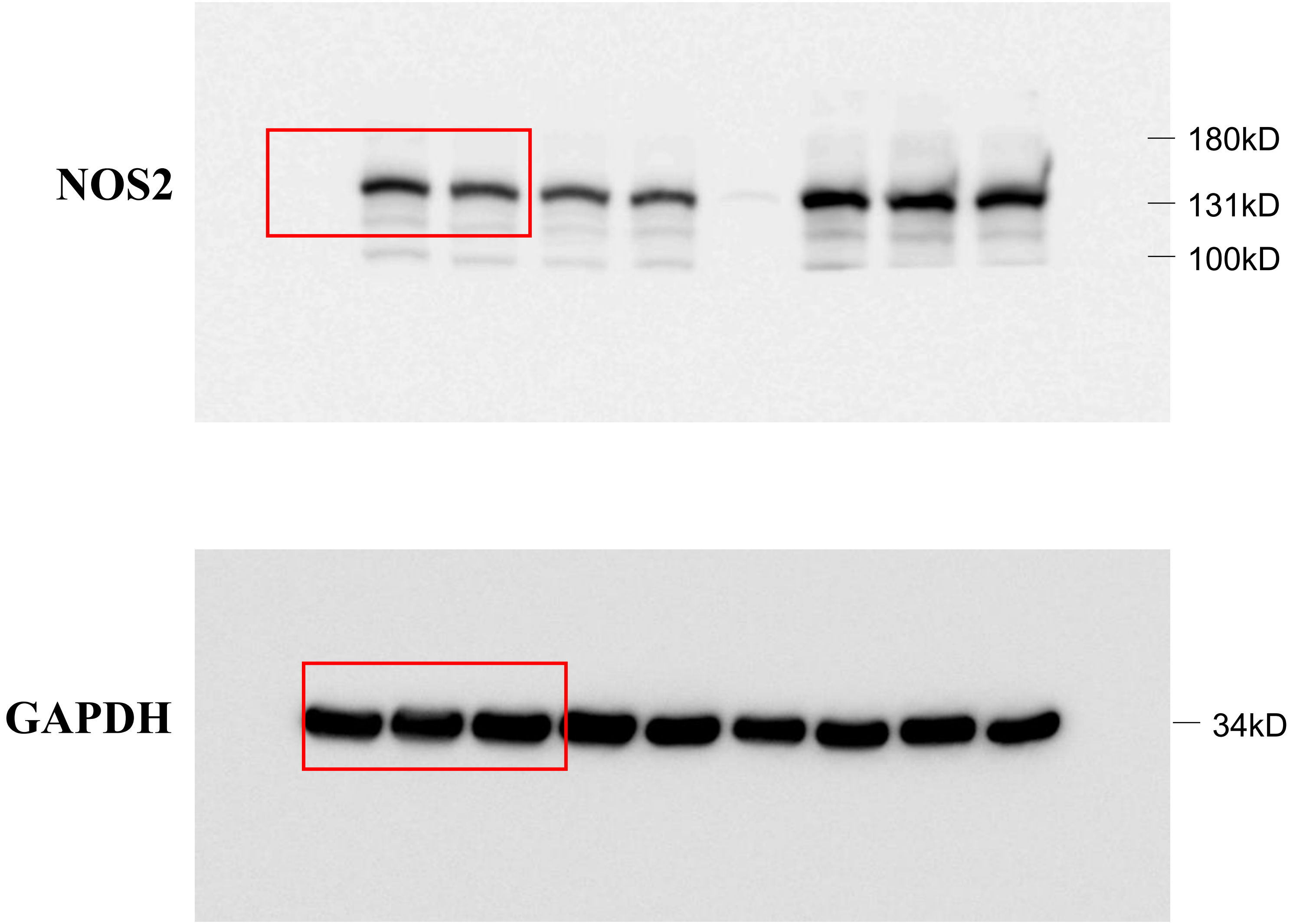

SFigure 4C

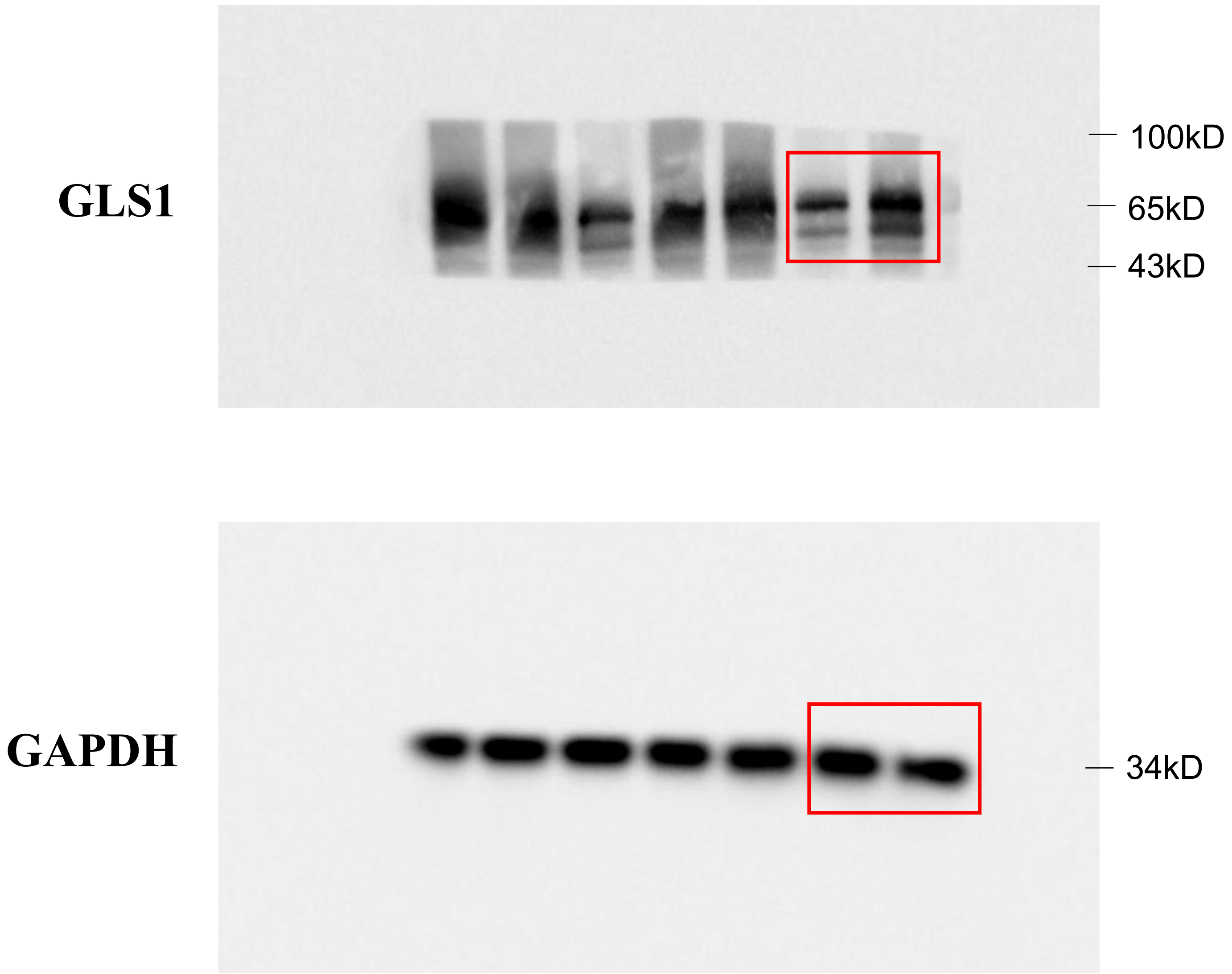

SFigure 4E

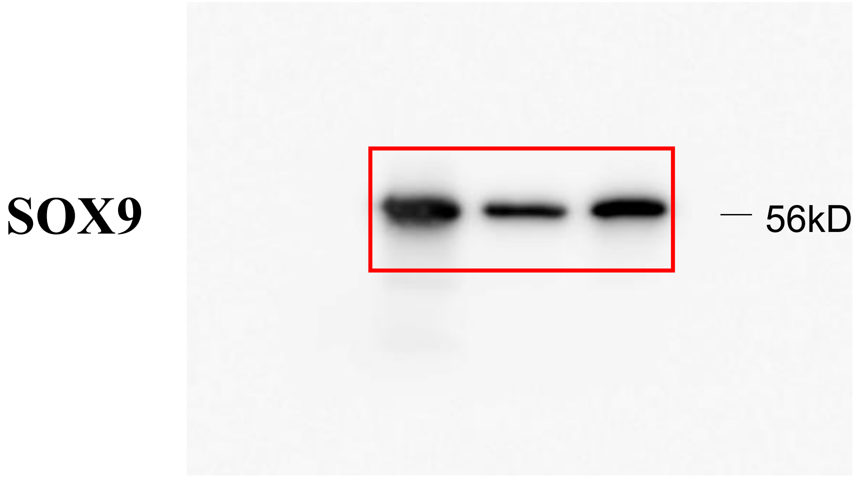
SOX9
56kD

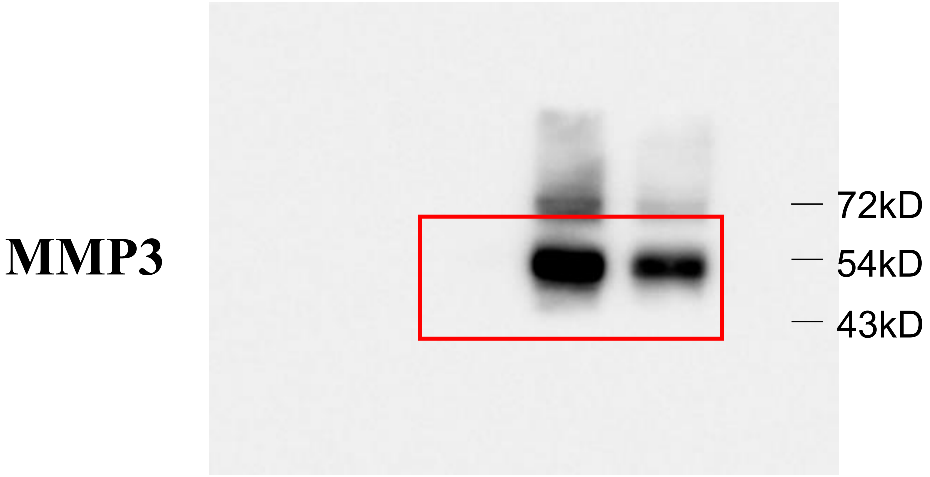
MMP3
72kD
54kD
43kD

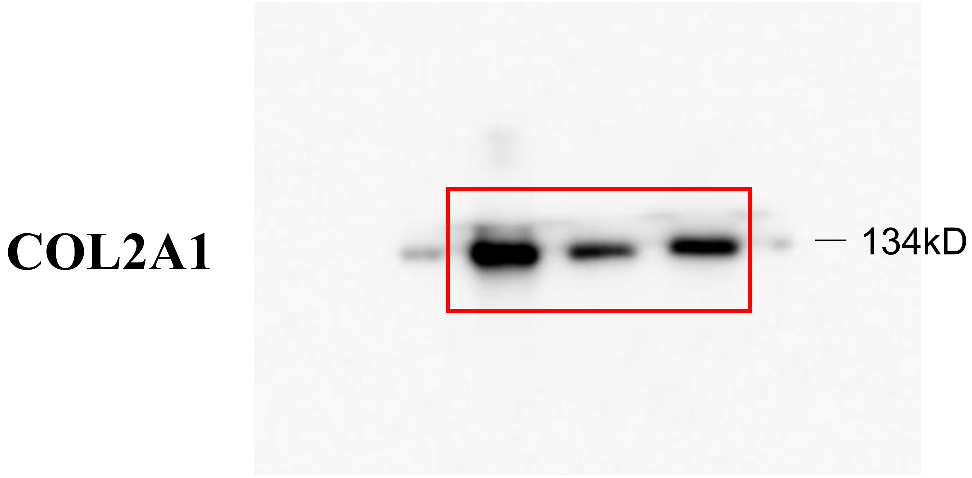
COL2A1
134kD

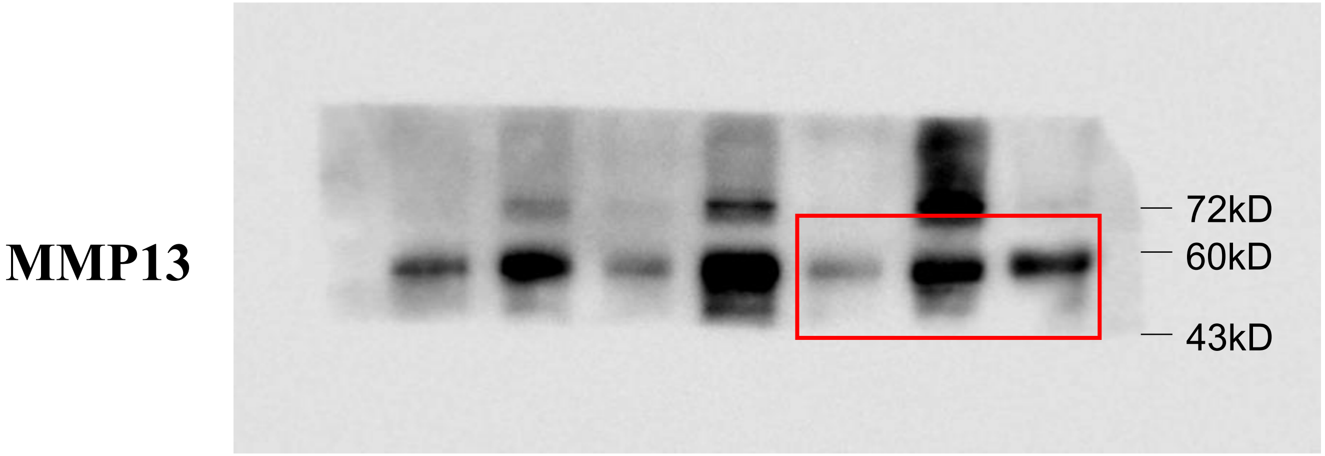
MMP13
72kD
60kD
43kD

SFigure 4E

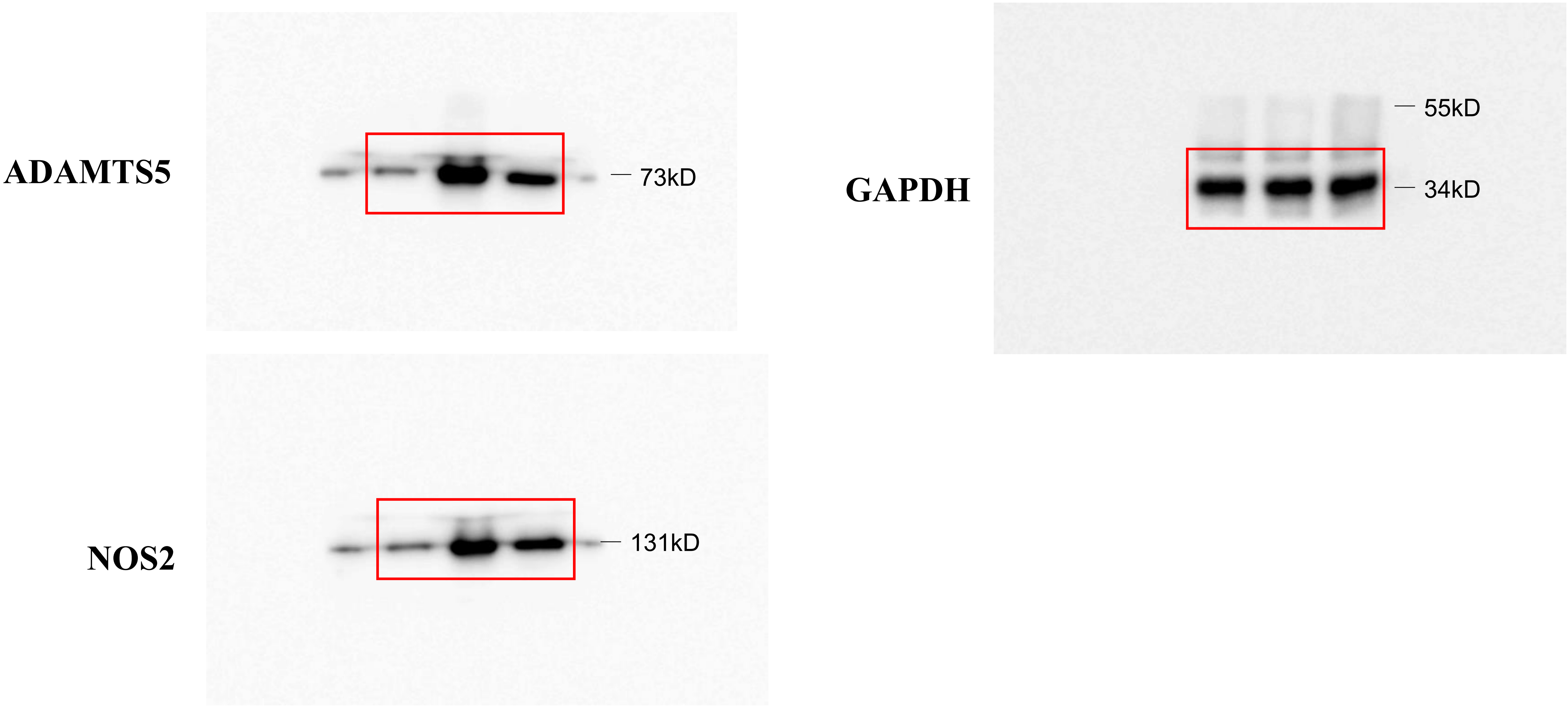

SFigure 8B

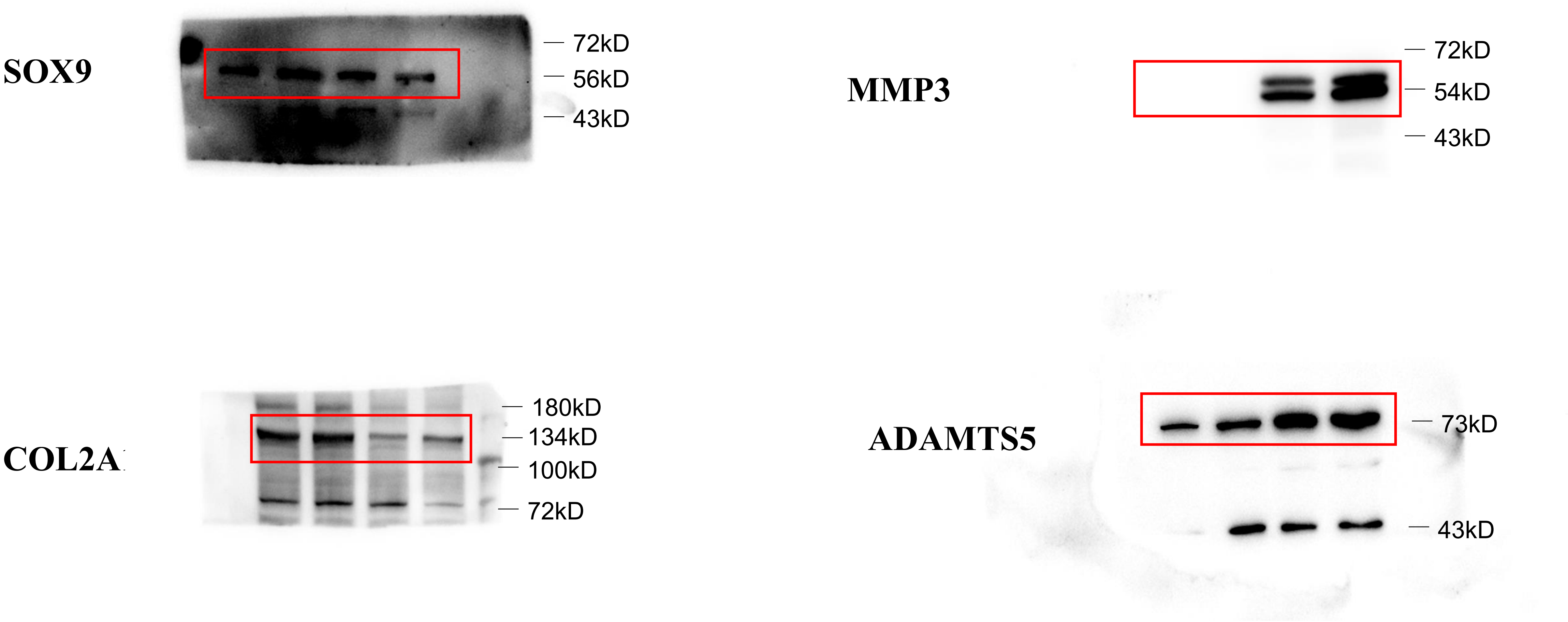

SOX9
72kD
56kD
43kD
MMP3
72kD
54kD
43kD
COL2A
180kD
134kD
100kD
72kD
ADAMTS5
73kD
43kD

SFigure 8B

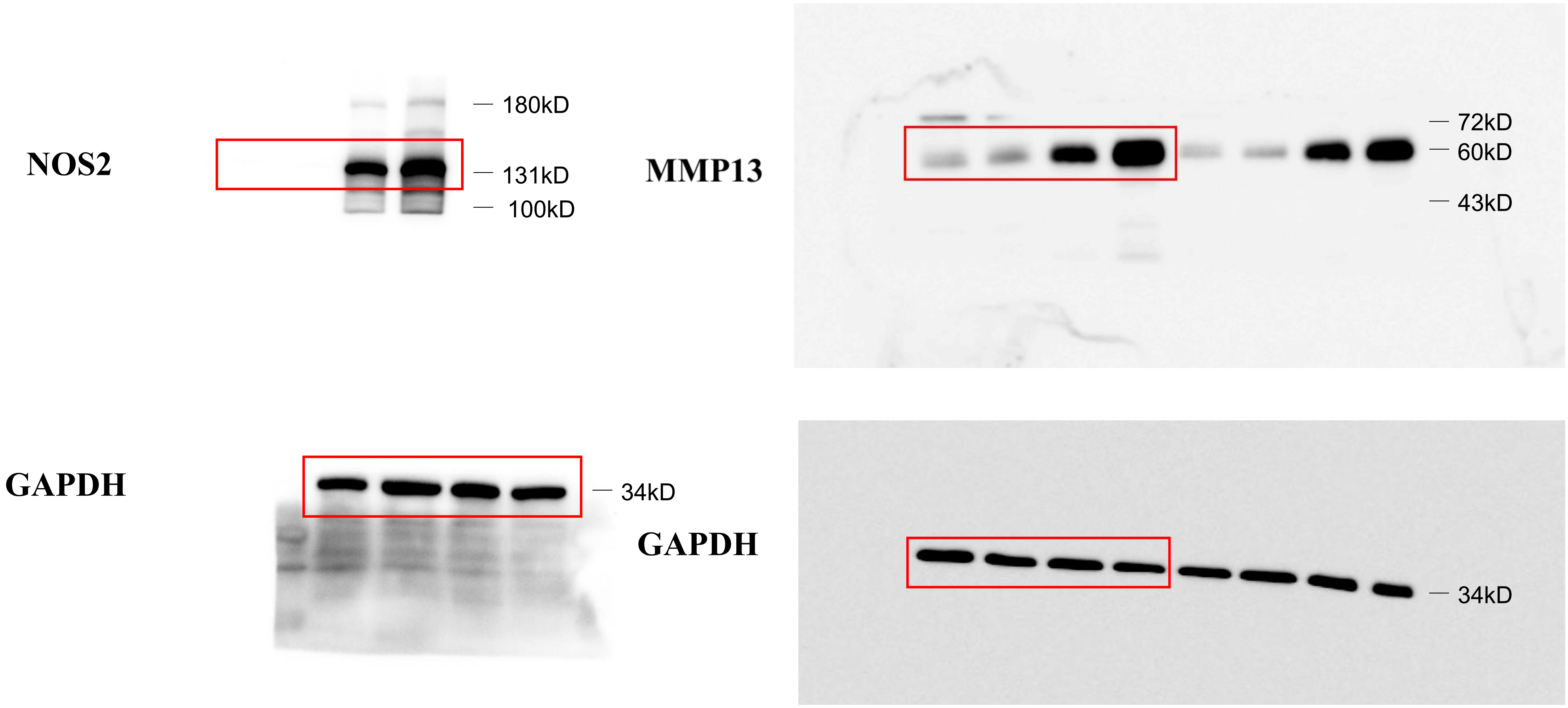
NOS2
180kD
131kD
100kD
MMP13
72kD
60kD
43kD
GAPDH
34kD
GAPDH
34kD

SFigure 8D

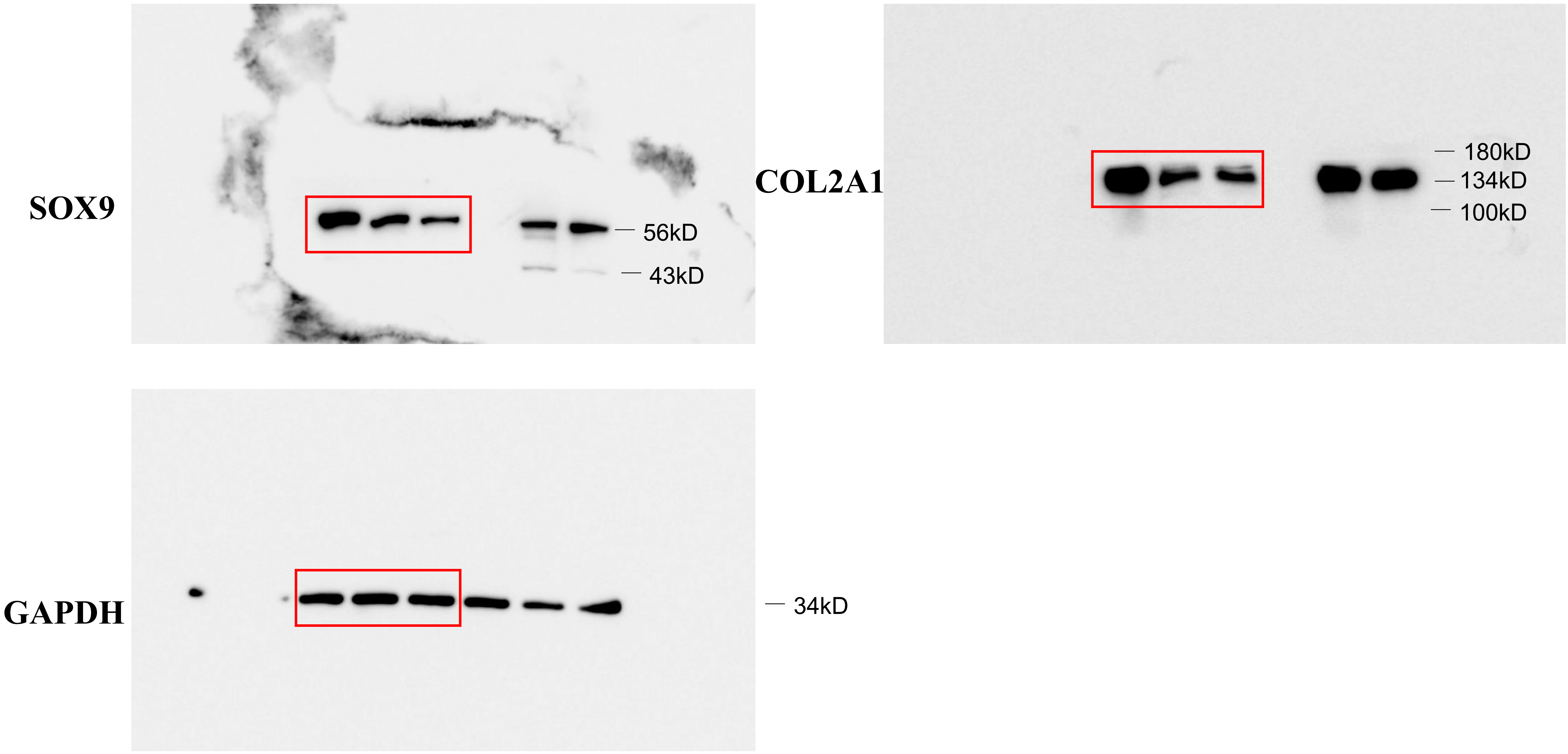

SFigure 8D

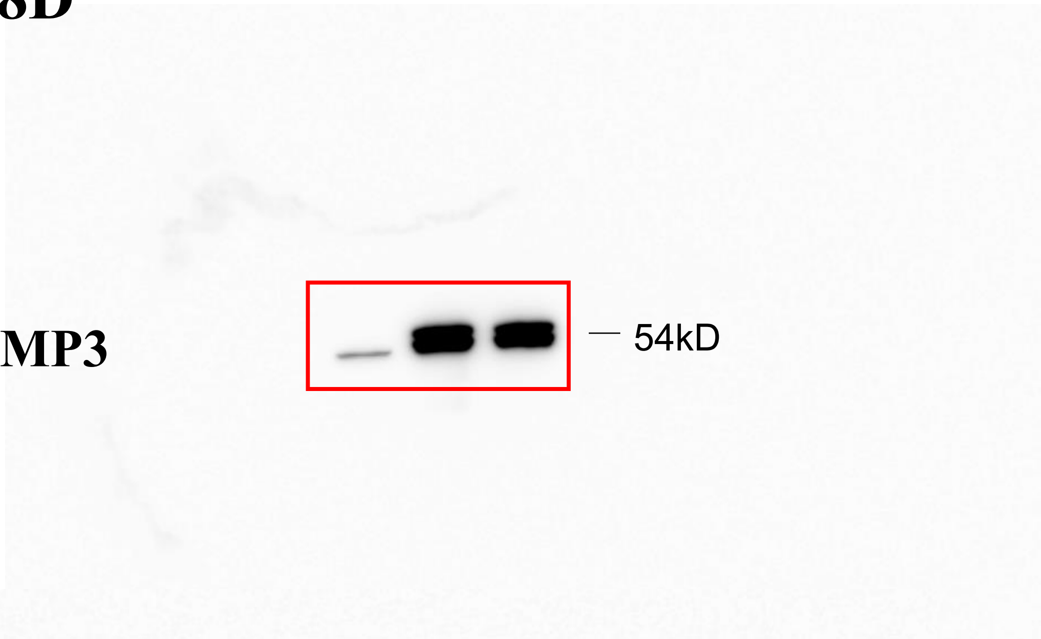

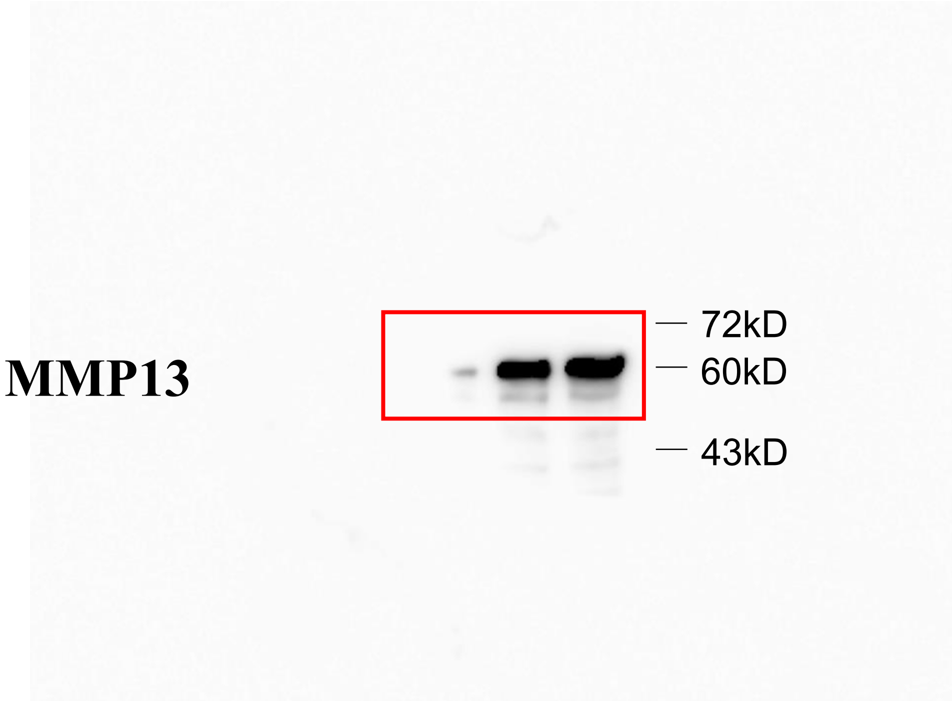

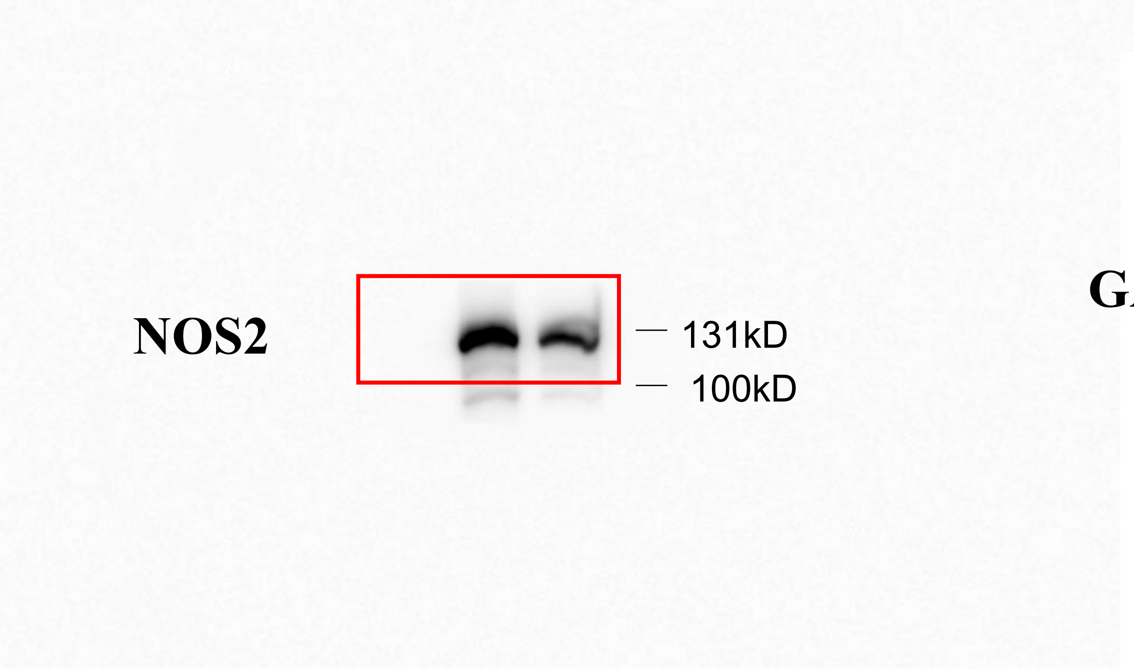

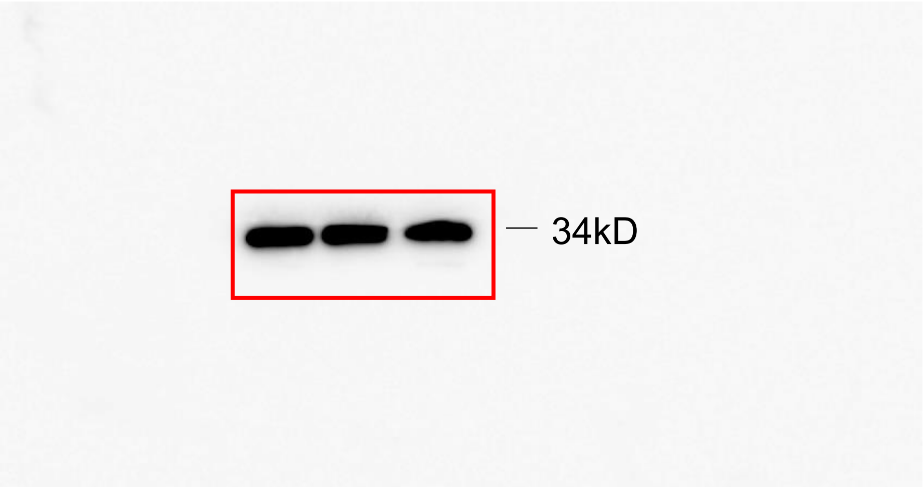

SFigure 9A

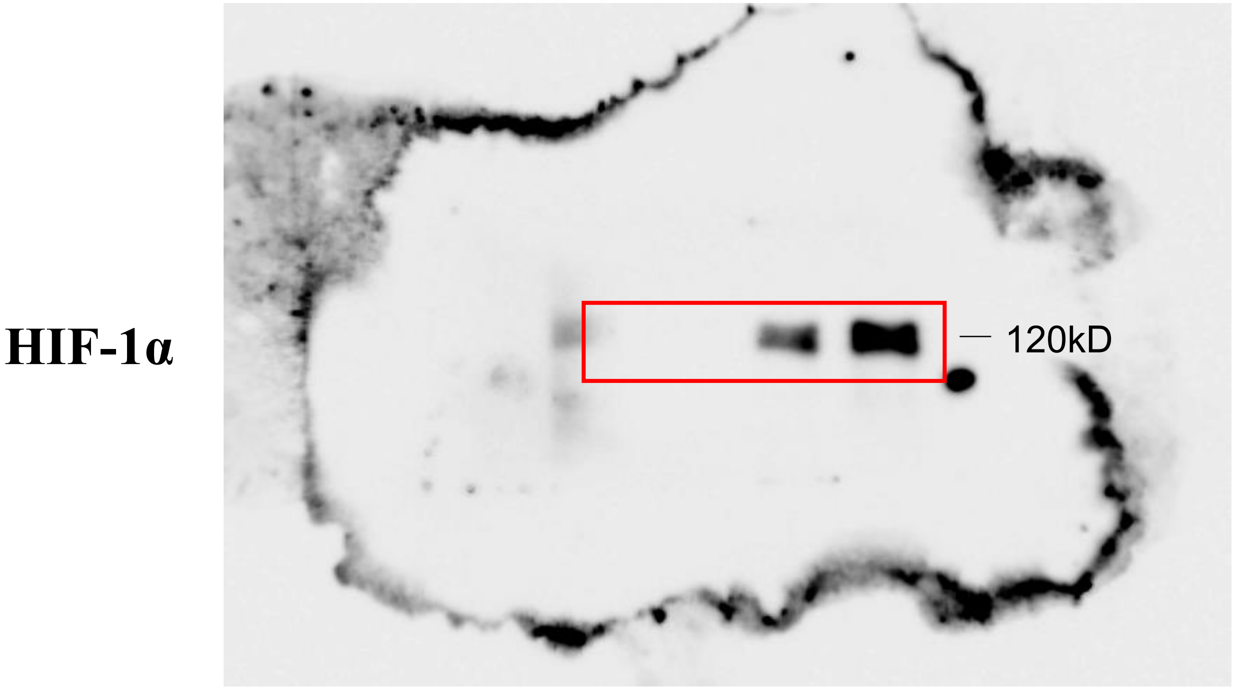

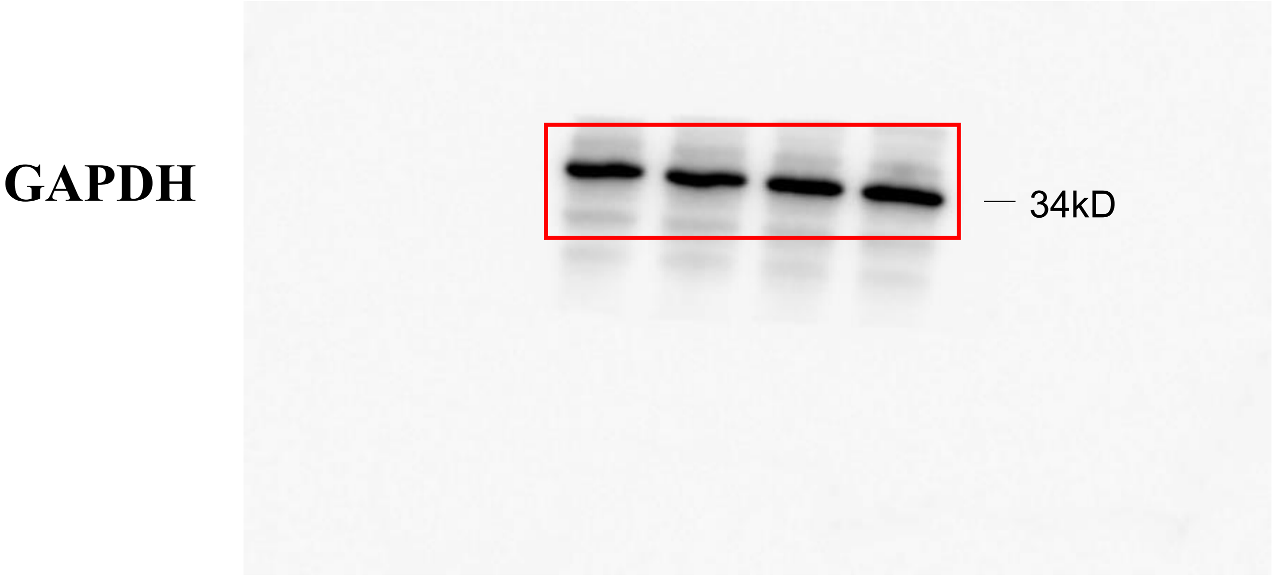

SFigure 9B

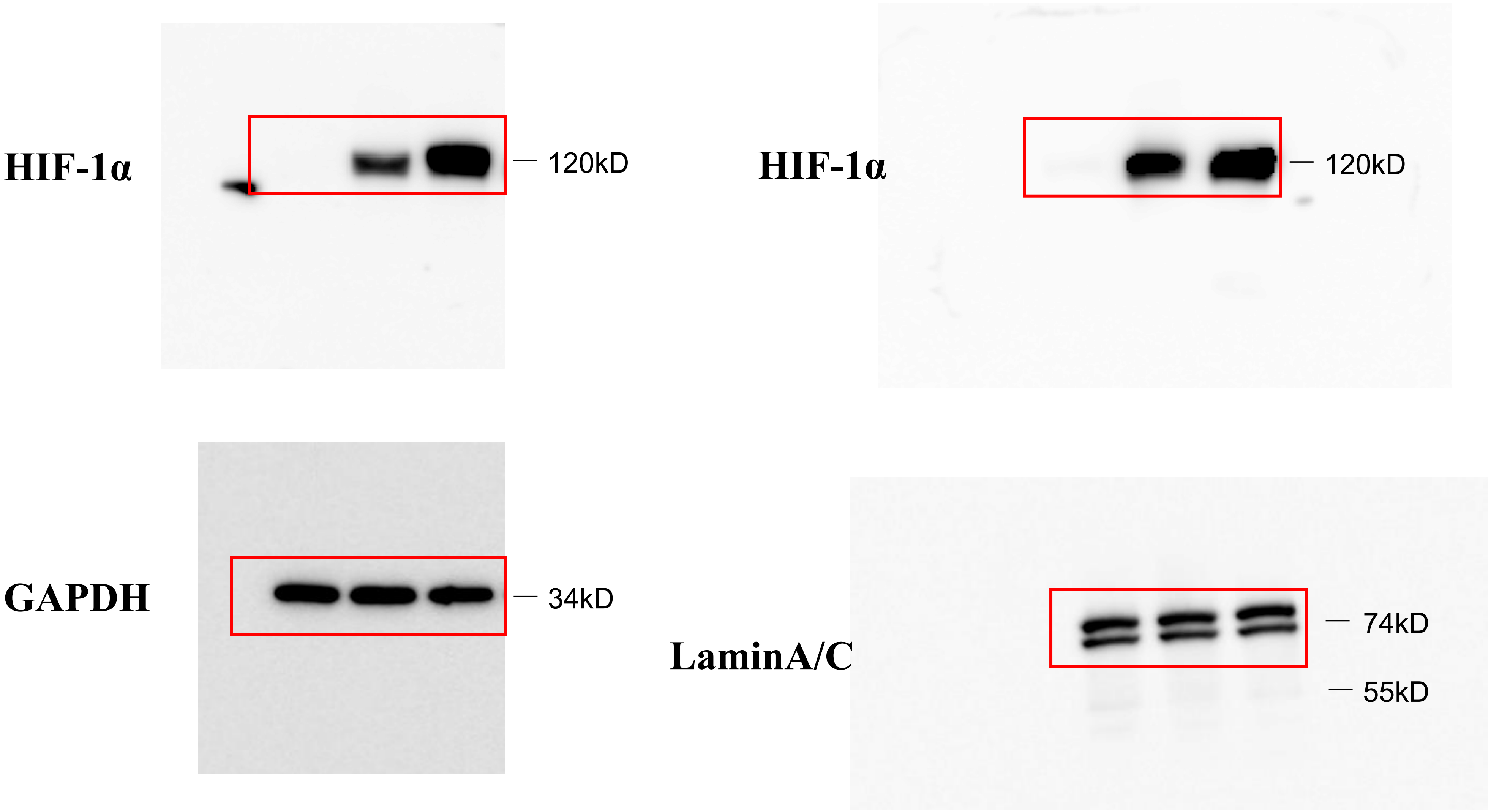

SFigure 9D

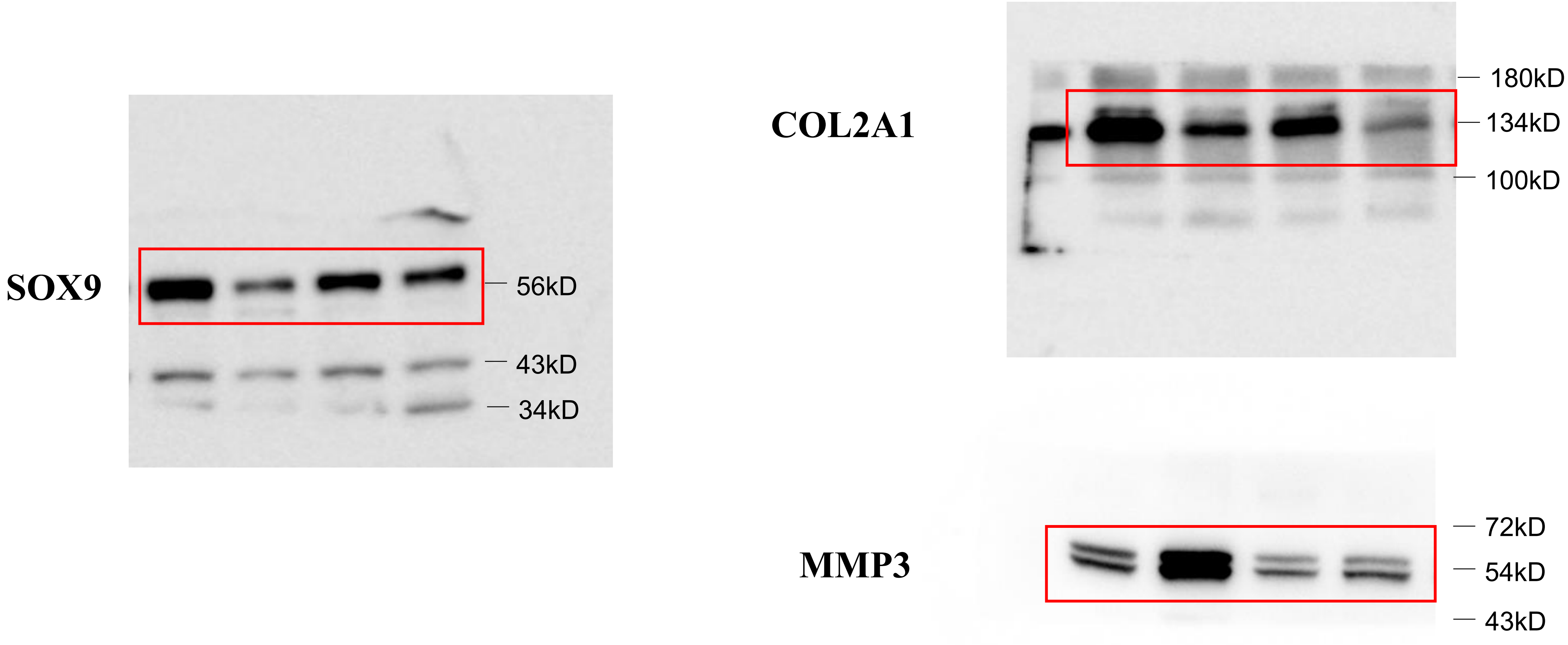
SOX9
56kD
43kD
34kD
COL2A1
180kD
134kD
100kD
MMP3
72kD
54kD
43kD

SFigure 9D

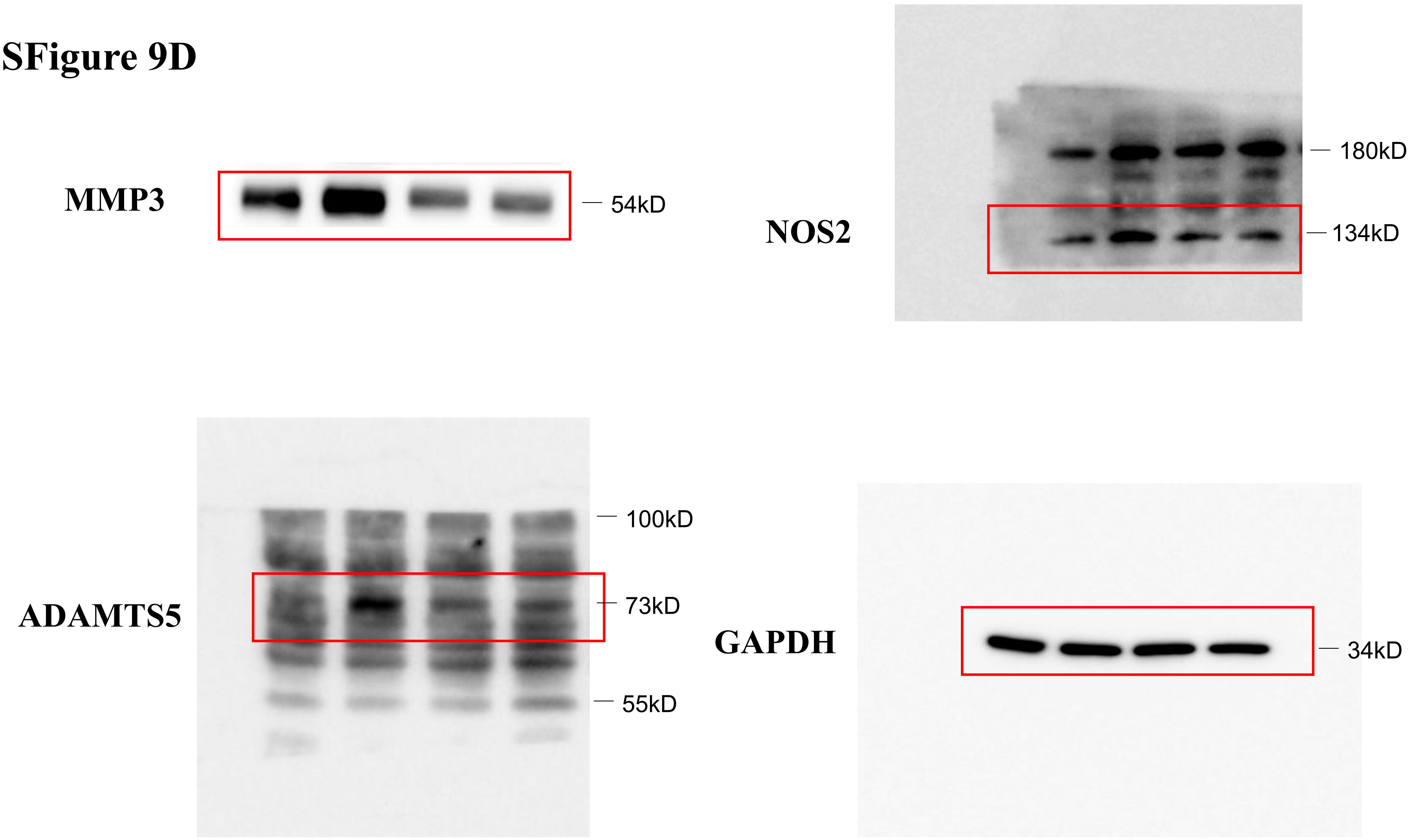
MMP3
54kD
NOS2
180kD
134kD
ADAMTS5
100kD
73kD
55kD
GAPDH
34kD

SFigure 12B

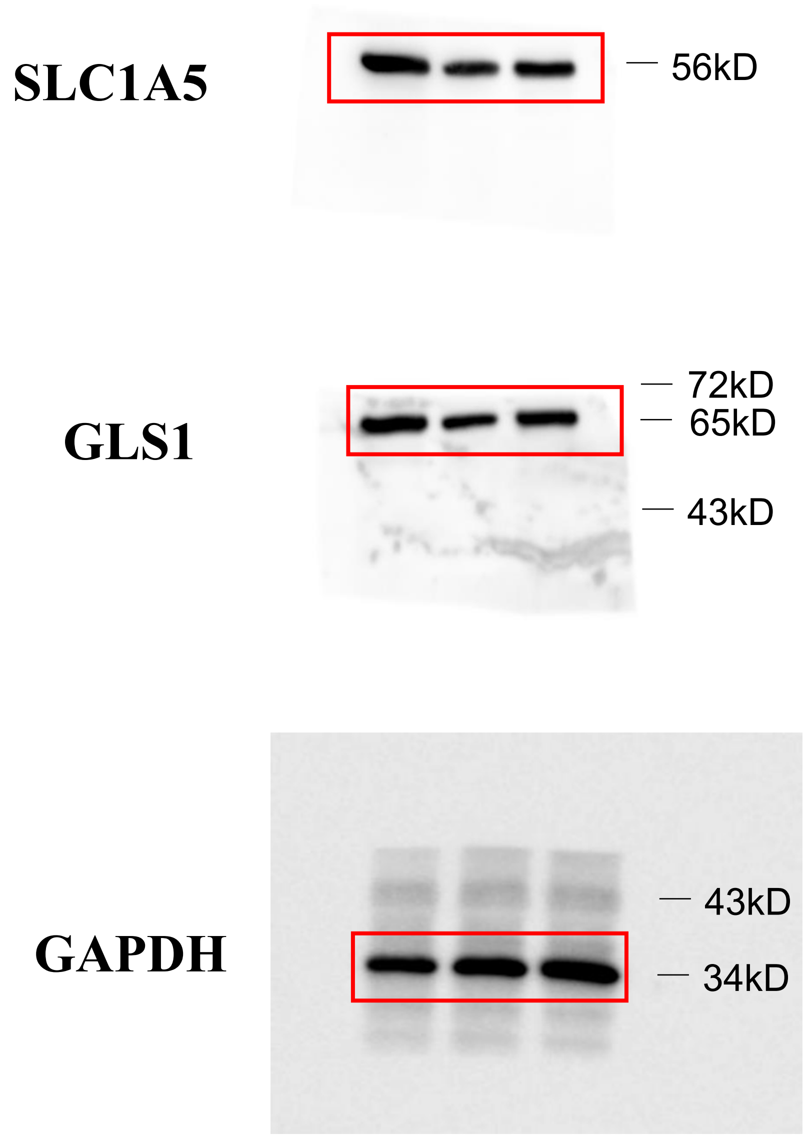

SFigure 12G

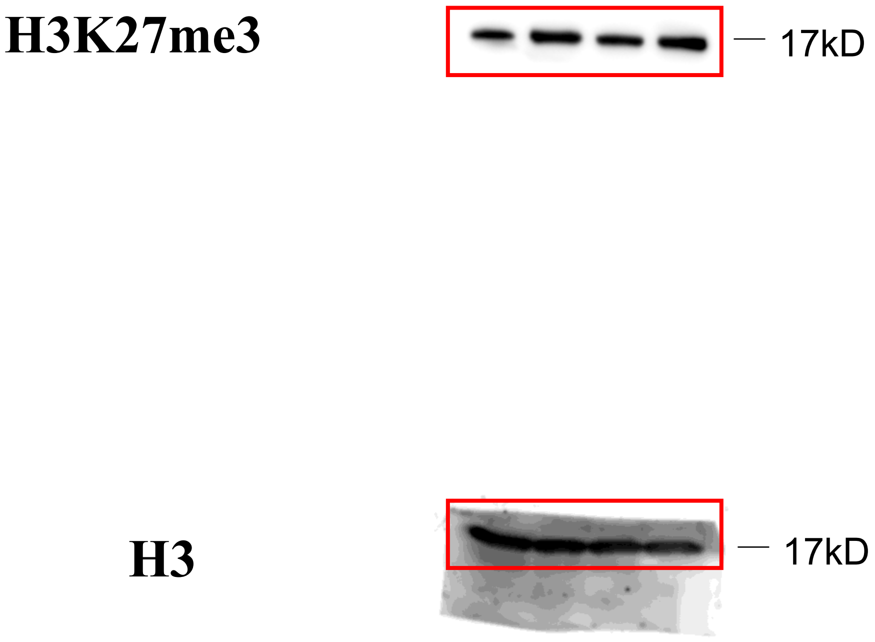

SFigure 12G

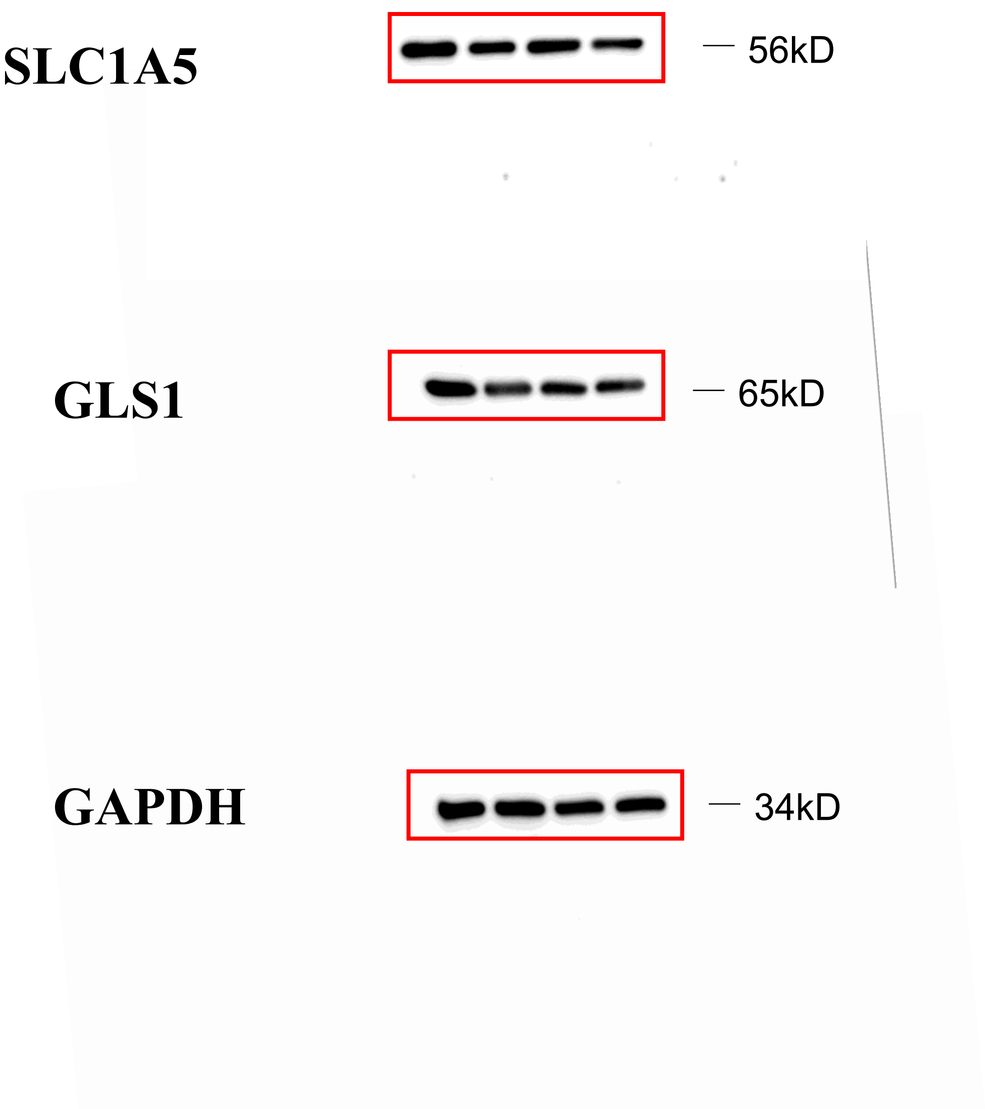

SFigure 13B

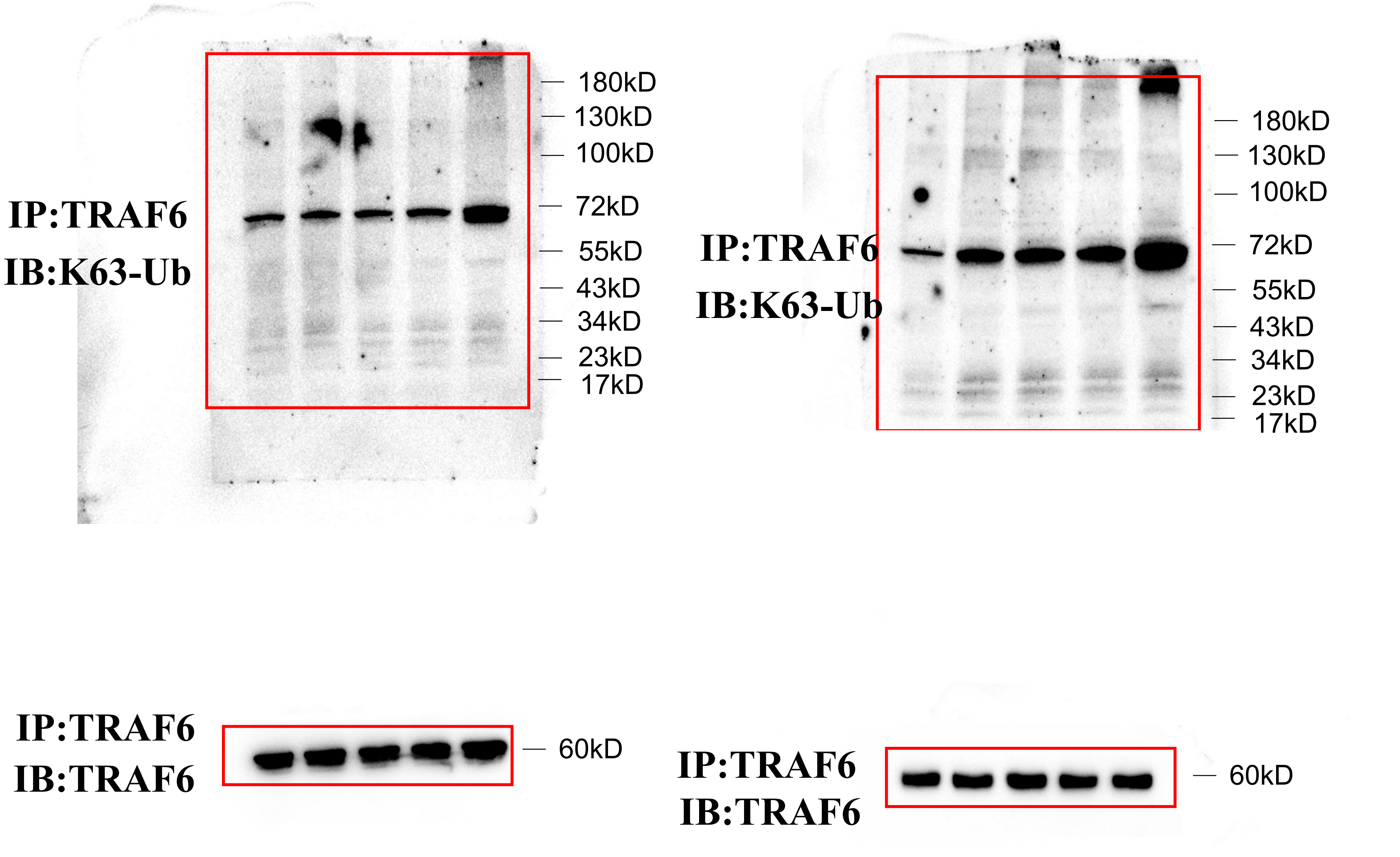
IP:TRAF6
IB:K63-Ub
180kD
130kD
100kD
72kD
55kD
43kD
34kD
23kD
17kD
IP:TRAF6
IB:K63-Ub
180kD
130kD
100kD
72kD
55kD
43kD
34kD
23kD
17kD
IP:TRAF6
IB:TRAF6
60kD
IP:TRAF6
IB:TRAF6
60kD

SFigure 13C

IP:HA
IB:Flag

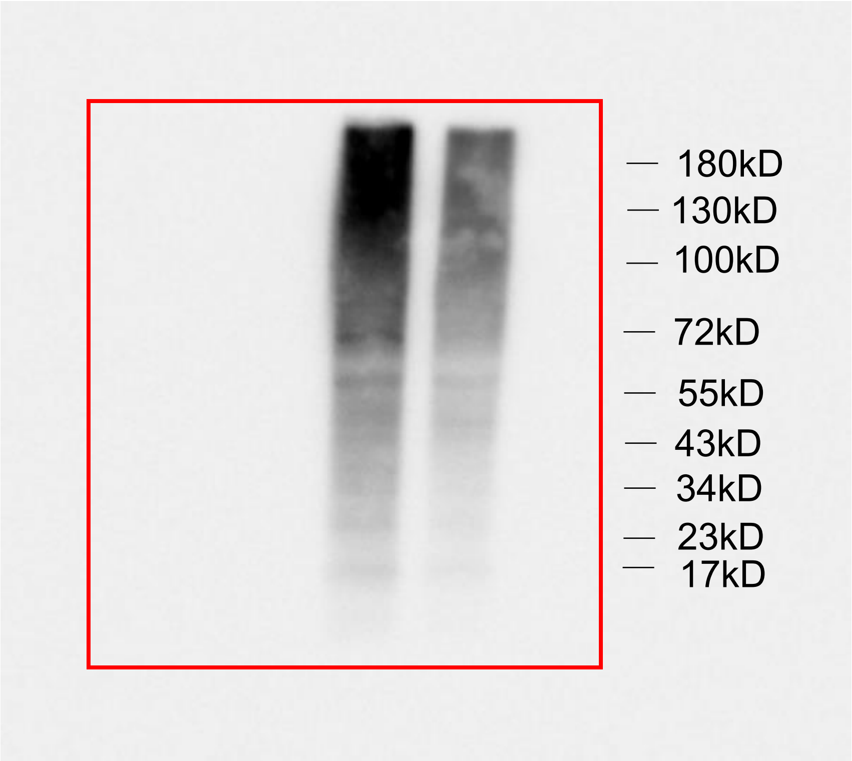

IP:HA
IB:HA

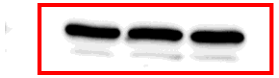

SFigure 13D

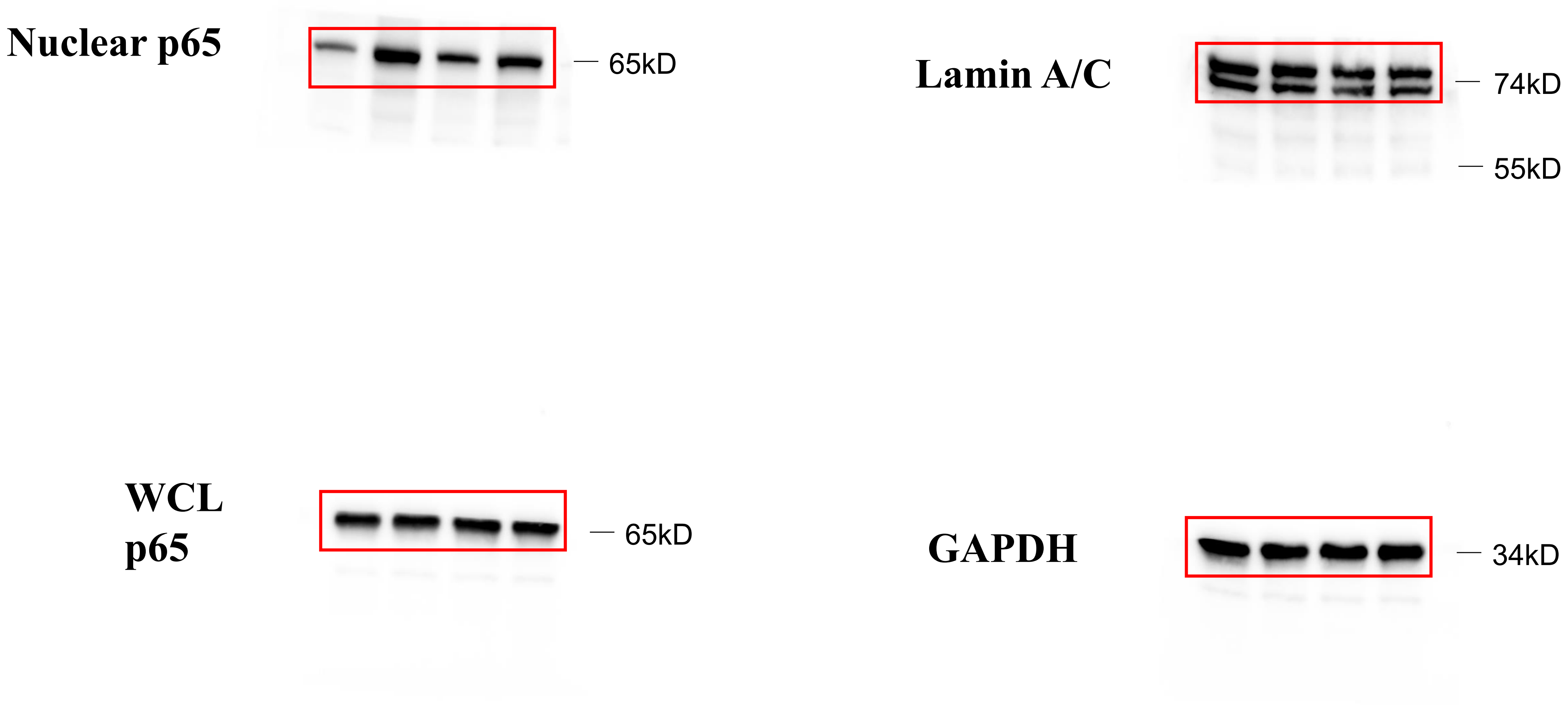

SFigure 13D

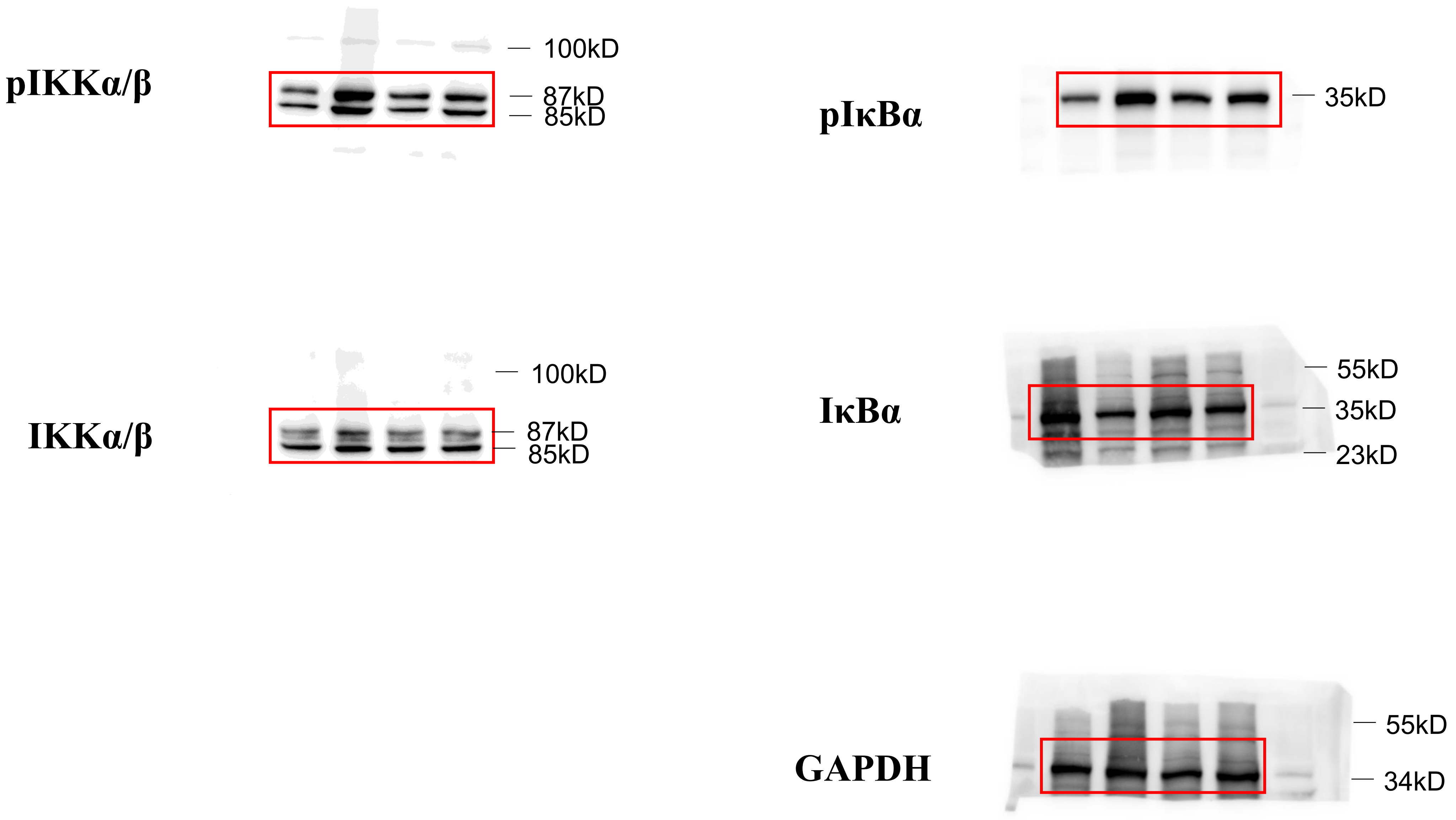
pIKKα/β
100kD
87kD
85kD
IKKα/β
100kD
87kD
85kD
pIκBα
35kD
IκBα
55kD
35kD
23kD
GAPDH
55kD
34kD

SFigure 13E

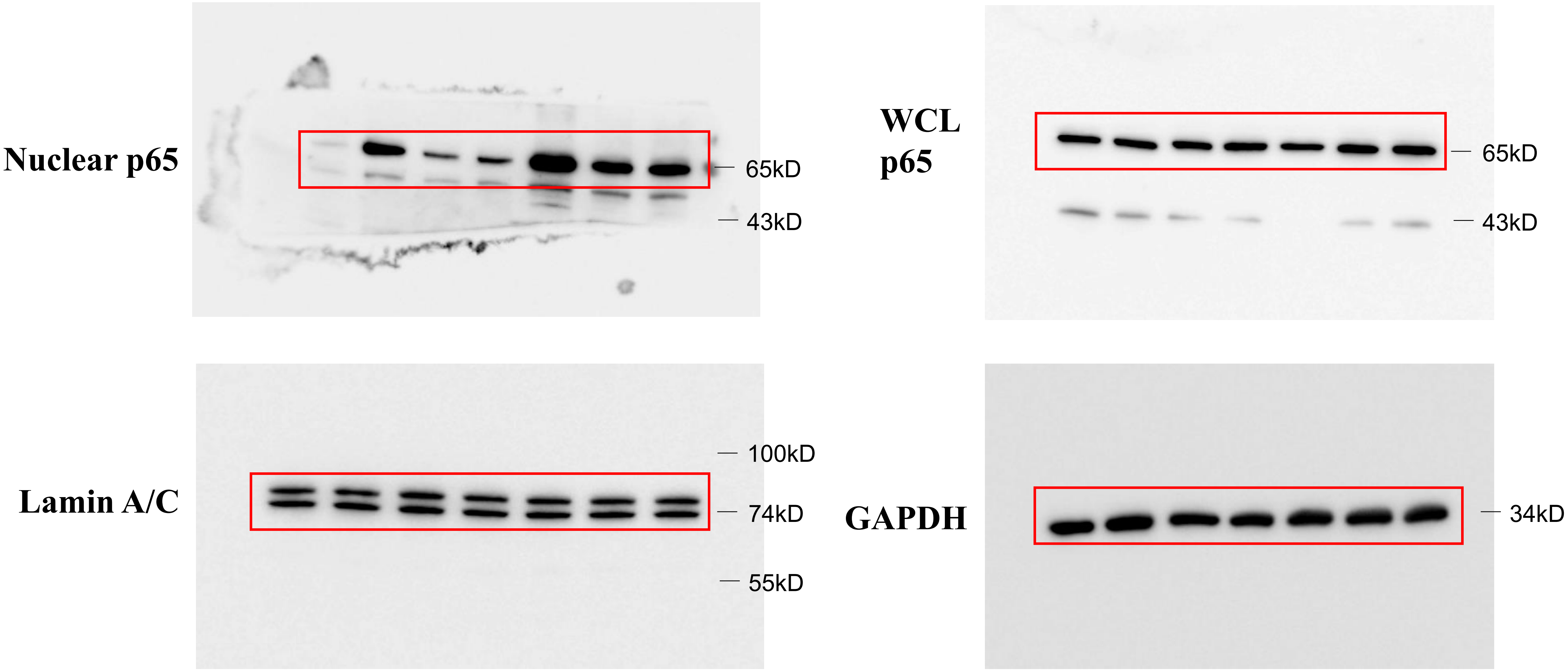
Nuclear p65
65kD
43kD
WCL
p65
65kD
43kD
Lamin A/C
100kD
74kD
55kD
GAPDH
34kD

SFigure 13E

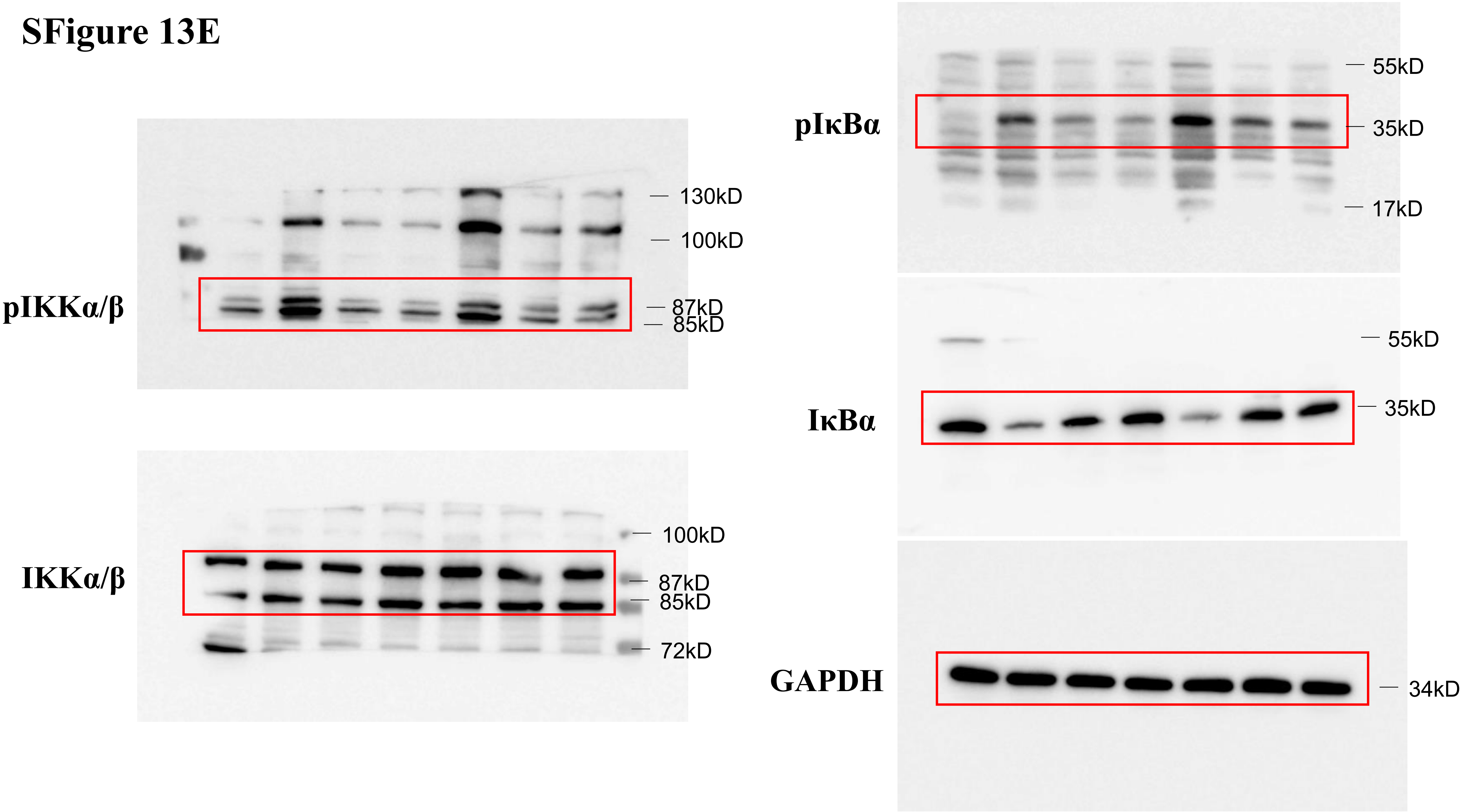
pIKKα/β
130kD
100kD
87kD
85kD
IKKα/β
100kD
87kD
85kD
72kD
pIκBα
55kD
35kD
17kD
IκBα
55kD
35kD
GAPDH
34kD

SFigure 13G

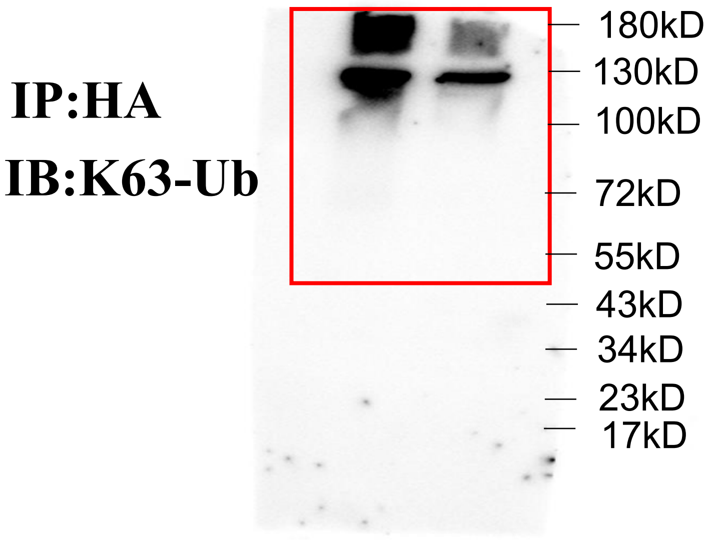
IP:HA
IB:K63-Ub
180kD
130kD
100kD
72kD
55kD
43kD
34kD
23kD
17kD

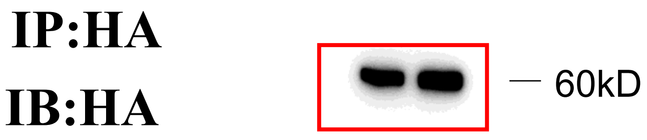
IP:HA
IB:HA
60kD
